# Stereochemical 2D NMR Assignment of Thietane, Synthesis and Antiplatelet Activity of Novel Thietane-Containing 1-Isobutylpurine-2,6-dione Derivatives as Potential GPIIb/IIIa Inhibitors

**DOI:** 10.3390/molecules31193521

**Published:** 2026-10-02

**Authors:** Ferkat A. Khaliullin, Zhekshen K. Mamatov, Iuliia V. Shabalina, Yi Wang, Aleksandr N. Lobov, Elena E. Klen, Kudaiberdi G. Kozhobekov, Dmitry A. Kudlay, Aleksandr V. Samorodov

**Affiliations:** 1 Department of Pharmaceutical Chemistry, Bashkir State Medical University, Lenina Str., 3, Ufa 450008, Russia; fil-ko@mail.ru (I.V.S.); lobovan@anrb.ru (A.N.L.); klen_elena@yahoo.com (E.E.K.); avsamorodov@gmail.com (A.V.S.); 2 Department of Pharmaceutical Business Organization and Pharmacognosy, Osh State University, Alymbek Datka Str., 331, Osh 723500, Kyrgyzstan; mamatovzhk@gmail.com (Z.K.M.);; 3 School of Pharmacy, Hangzhou Normal University, Yuhangtan, 2318, Hangzhou 310030, China; yi.wang1122@hznu.edu.cn; 4 Department of Pharmacology, Sechenov First Moscow State Medical University ( Sechenov University), Moscow 119435, Russia; kudlay_d_a@staff.sechenov.ru; 5 Faculty of Bioengineering and Bioinformatics, Lomonosov Moscow State University, Leninskie Gory 1, Building 73, Moscow 119234, Russia

**Keywords:** 1-isobutylpurine-2,6-diones, thietanes, 2D NMR, conformational analysis, antiplatelet activity, GPIIb/IIIa inhibitors

## Abstract

1-Isobutylpurine-2,6-diones possess a broad spectrum of pharmacological activities, highlighting their relevance in the search for new biologically active agents. In this study, we report the synthesis of a series of novel thietane-containing 1-isobutylpurine-2,6-dione derivatives. A crucial aspect of this work is the detailed stereochemical and conformational analysis of the thietanyl substituent. For the first time, the unambiguous assignment of axial and equatorial protons within the thietane ring was achieved using 2D NMR spectroscopy, exemplified by a comprehensive study of the starting substance. Furthermore, the newly synthesized compounds were evaluated for their antiplatelet activity, revealing inhibition of platelet aggregation exceeding that of acetylsalicylic acid. Based on structural analogy to an established antiplatelet agent, we hypothesize that the observed biological effect is mediated via the inhibition of the glycoprotein IIb/IIIa (GPIIb/IIIa) receptor complex. These findings demonstrate that the incorporation of a thietane fragment into the 1-isobutylxanthine scaffold yields promising candidates for the development of novel GPIIb/IIIa-targeted antiplatelet therapeutics.

## 1. Introduction

**Figure 1:**
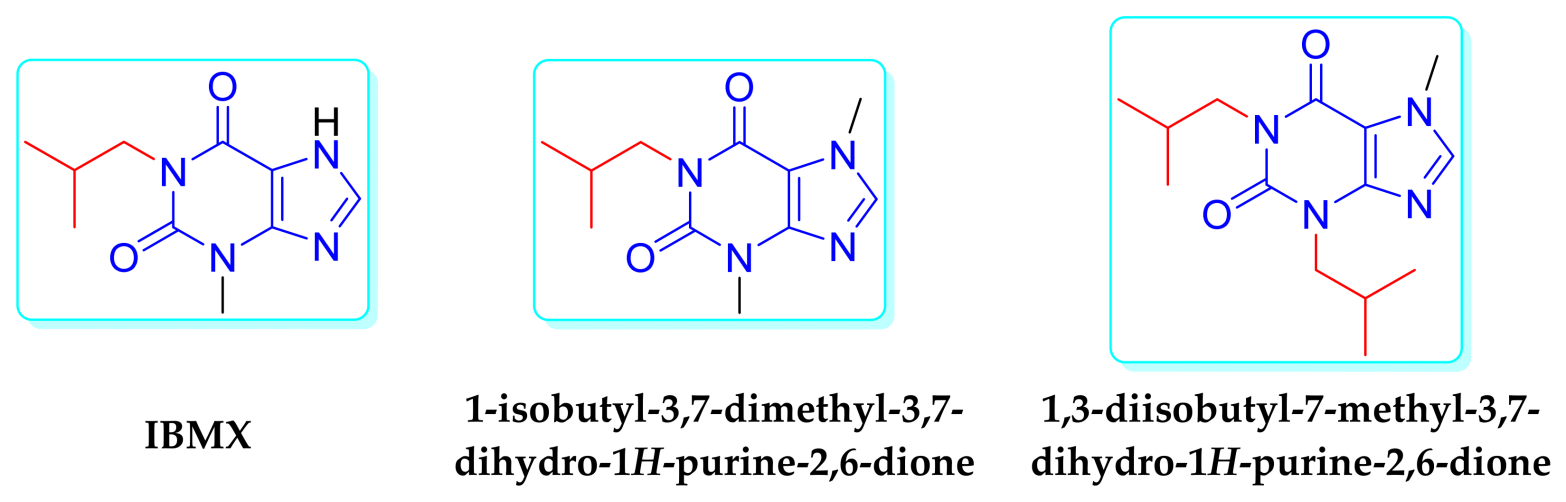
Reported 1-isobutylpurine-2,6-diones as prototypes of the target compounds.

1-Isobutyl-3-methyl-3,7-dihydro-1*H*-purine-2,6-dione (IBMX, Figure 1) is a well-known phosphodiesterase inhibitor [1] employed as an agent for elevating cAMP levels in studies of various intracellular mechanisms [2].

IBMX enhanced 5-hydroxytryptamine-induced relaxation of the circular smooth muscle of the human colon, which confirms the involvement of cAMP in the 5-HT_4_ receptor binding mechanism [3]; IBMX led to the phosphorylation of the cardiac-type α1 subunit of voltage-dependent calcium channels of the pancreatic *β*-cell [4]; and IBMX, via phosphorylation, affected adrenergic chronotropism in excised cardiac muscles from guinea pigs [5]. The addition of IBMX resulted in the phosphorylation and degradation of the Acheron protein, which drives metastatic processes in some cancers and protects some cancers from death [6].

IBMX is used as part of the maintenance medium for differentiation of fibroblasts into adipocytes for the bioactivity study of transported liposomal forms of insulin [7], and IBMX is also used as part of the adipogenic cocktails for differentiation of adipose stem cells to differentiate into mature adipocytes in the production of cultured fat [8].

The effects of 1-isobutylpurine-2,6-diones are not always associated with an increase in intracellular cAMP levels. 1-Isobutyl-3,7-dimethyl-3,7-dihydro-1*H*-purine-2,6-dione which exhibits no inhibitory activity against phosphodiesterases in contrast to IBMX [9], has been shown to activate the cystic fibrosis transmembrane conductance regulator chloride channel [10]; the review [11] indicated that such purine-2,6-diones may become potential therapeutic agents to cure cystic fibrosis, including dry eye syndrome [12]. Furthermore, 1-isobutyl-3,7-dimethyl-3,7-dihydro-1*H*-purine-2,6-dione has been proposed as an effective neuroleptic agent in contrast to the well-known psychostimulatory 3,7-dihydro-1*H*-purine-2,6-diones, caffeine or theophylline [13].

1,3-Diisobutyl-3,7-dihydro-1*H*-purine-2,6-dione displayed selectivity for A_1_-adenosine receptors [14,15], producing the bronchodilator effect [16]; the series of 1,3-diisobutyl-3,7-dihydro-1*H*-purine-2,6-diones are patented as A_2B_ adenosine receptor antagonists, useful for treating various disease states, including asthma and diarrhea [17].

Derivatives of 1-isobutylpurine-2,6-dione have been investigated as selective agonists for cannabinoid receptor GPR18, which constitutes a promising drug target in immunology and cancer [18].

The 7-thietanyl-substituted 1-alkyl-3,7-dihydro-1*H*-purine-2,6-diones that we synthesized, containing amine residues at position 8, exhibit antiplatelet activity [19,20]; however, their 1-isobutyl analogs remained unstudied.

The present work reports the synthesis of 8-amino-substituted 1-isobutyl-3-methyl-3,7-dihydro-1*H*-purine-2,6-diones containing a thietane ring at position 7 and evaluates their antiplatelet activity.

## 2. Results

### 2.1. Chemistry

**Scheme 1:**
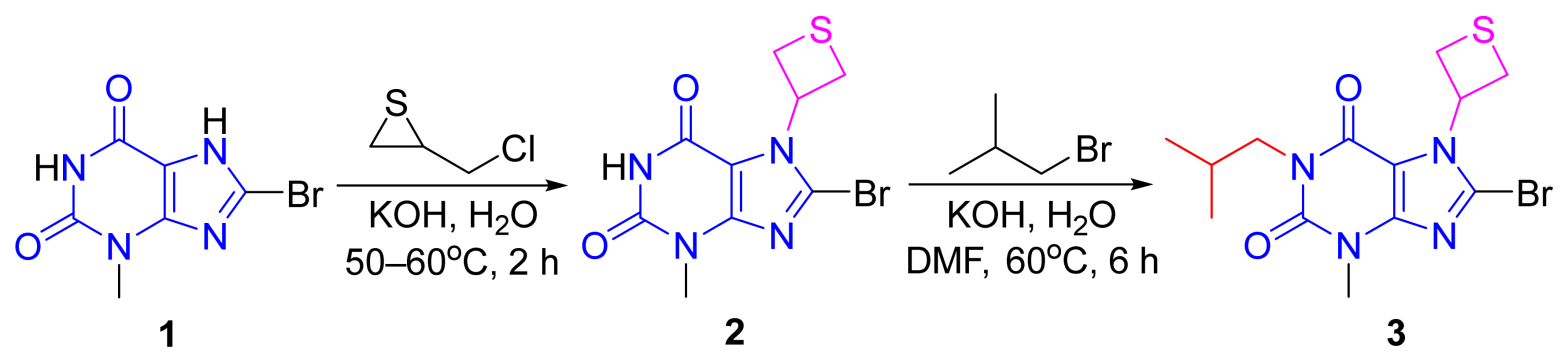
Synthesis of the key intermediate **3**.

The key intermediate, 8-bromo-1-isobutyl-3-methyl-7-(thietan-3-yl)-3,7-dihydro-1*H*-purine-2,6-dione (**3**) [21], was prepared by sequential introduction of a thietane ring into the *N*^7^-position of 8-bromo-3-methyl-3,7-dihydro-1*H*-purine-2,6-dione (**1**) by reaction with 2-(chloromethyl)thiirane in water and alkylation of the *N*^1^ position of the resulting 8-bromo-3-methyl-7-(thietan-3-yl)-3,7-dihydro-1*H*-purine-2,6-dione (**2**) with isobutyl bromide in DMF in the presence of potassium hydroxide (Scheme 1).

8-Bromo-1-isobutyl-3-methyl-7-(1-oxothietan-3-yl)-3,7-dihydro-1*H*-purine-2,6-dione (**4**) was synthesized by oxidation of 8-bromopurine-2,6-dione **3** with hydrogen peroxide in 83% yield, 8-bromo-1-isobutyl-3-methyl-7-(1,1-dioxothietan-3-yl)-3,7-dihydro-1*H*-purine-2,6-dione (**5**)—by oxidation of 8-bromopurine-2,6-dione **3** with potassium permanganate in 79% yield in acetic acid.

**Scheme 2:**
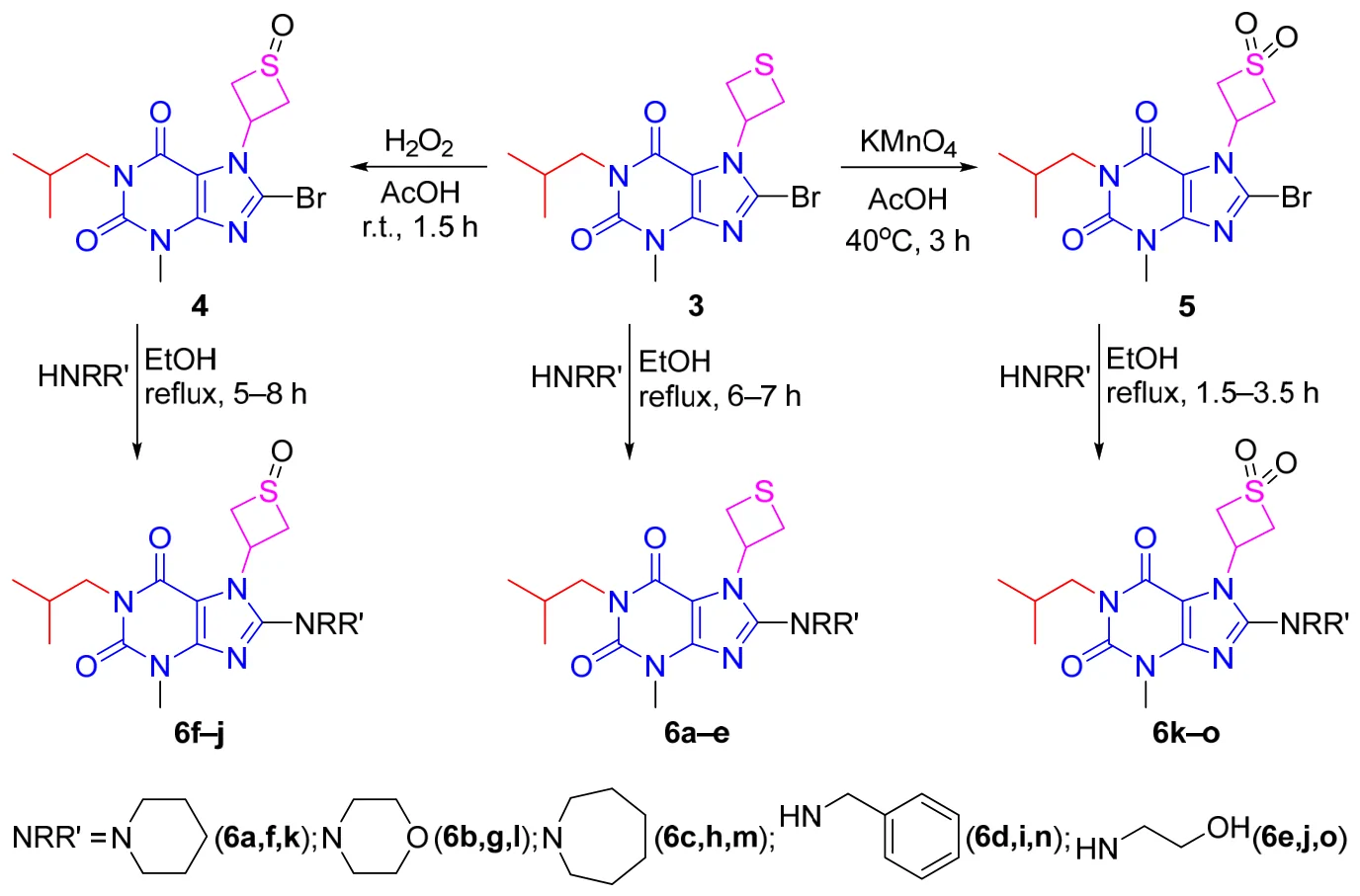
Synthesis of 8-amino-substituted 1-isobutyl-3-methyl-3,7-dihydro-1*H*-purine-2,6-diones containing thietane (**6a**–**e**), thietane oxide (**6f**–**j**) and thietane dioxide rings (**6k**–**o**).

Nucleophilic substitution reaction of bromine atom in compounds **3**–**5** with amines in ethanol resulted in the formation of 8-amino-substituted 1-isobutyl-3-methyl-7-(thietan-3-yl)-3,7-dihydro-1*H*-purine-2,6-diones (**6a**–**e**), 1-isobutyl-3-methyl-7-(1-oxothietan-3-yl)-3,7-dihydro-1*H*-purine-2,6-diones (**6f**–**j**) and 1-isobutyl-3-methyl-7-(1,1-dioxothietan-3-yl)-3,7-dihydro-1*H*-purine-2,6-diones (**6k**–**o**) in 35–83% yield (Scheme 2).

The structures of the synthesized compounds were confirmed by IR, NMR spectroscopy and mass spectrometry data. For full spectral data, see Supplementary Information.

### 2.2. Structural Investigation of the Thietanyl Substituent

**Figure 2:**
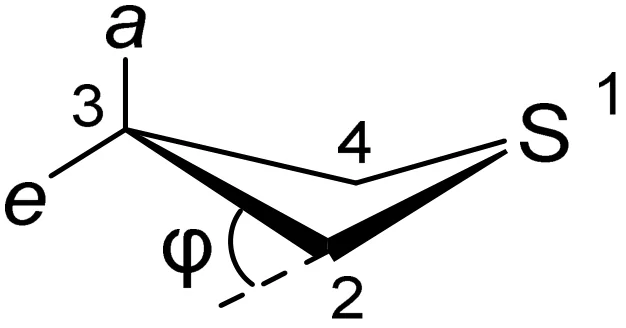
Geometry of the thietane ring.

The four-membered thietane ring adopts a folded conformation (with a puckering angle *φ* = 21°). Consequently, its 3-monosubstituted derivatives, such as the 7-(thietan-3-yl)purine-2,6-diones, can exist as two conformers featuring the substituent in either the axial (*a*) or equatorial (*e*) position (Figure 2). For steric reasons, the conformers with the substituent in the equatorial position are more stable.

A detailed investigation of the geometry of the thietanyl substituent was performed for compound **2**. The structure of the investigated compound was obtained by geometry optimization and vibrational frequency calculations using the Gaussian 16 program package [22] at the B3LYP [23,24,25]/6-311++G(d,p) [26,27,28] and M06-2X/6-311+G(d,p) [29] levels of theory. The choice of the B3LYP/6-311++G(d,p) level is justified by its successful application to related sulfur-containing heterocyclic systems, in particular, for the calculation of structural parameters and spin–spin coupling constants of thiane derivatives [30]. The use of the additional M06-2X functional is motivated by its ability to properly account for dispersion interactions, which is crucial for the reliable description of conformational equilibria and inversion barriers in strained four-membered rings, including the thietane ring [31,32]. According to the computational data for compound **2**, the C-S bond length is 1.842 Å, the C-C bond length is 1.537 Å, and the C-S-C bond angle is 78°, which is comparable to literature data [33].

**Figure 3:**
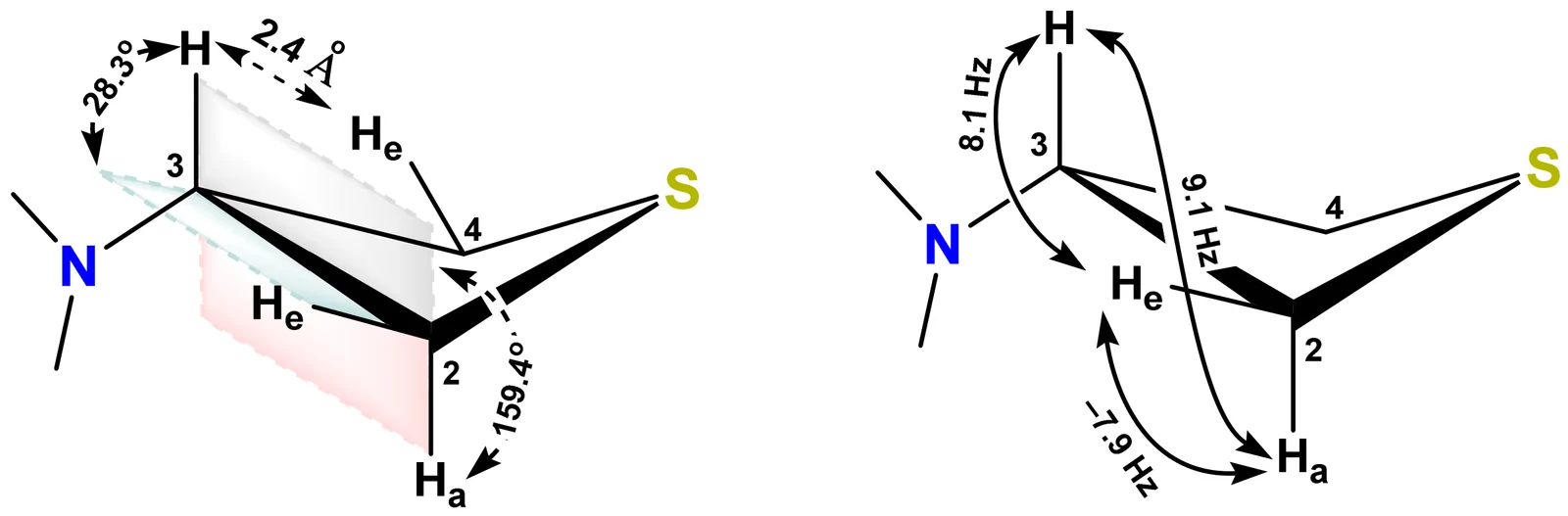
Calculated dihedral angles and ^2^*J_HH_* and ^3^*J_HH_* coupling constants for compound **2**.

The assignment of the methylene proton signals at *C*^2,4^ to the pseudoaxial and pseudoequatorial positions was performed based on the analysis of signal multiplet splitting and NOESY data. In the ^1^H NMR spectrum, the proton at *C*^3^ appears as a quintet at 5.82 ppm. The axial (*H_a_*) and equatorial (*H_e_*) hydrogen atoms at the *C*^2,4^ positions are magnetically non-equivalent and appear as a doublet of doublets at 4.22 ppm and as a triplet at 3.32 ppm, with a chemical-shift difference of approximately 0.9 ppm. To achieve reliable signal assignment in the ^1^H NMR spectrum and to confirm the spatial structure of the thietane ring, quantum chemical calculations of spin–spin coupling constants were performed (Table 1). The *J_HH_* coupling constants were calculated using the GIAO method at the mPW1PW91/6-311+G(2d,p) level of theory [34] with the polarizable continuum model (PCM) for DMSO. For comparison with the experimental values, total *J_HH_* constants were used, including all contributions: Fermi contact, spin–dipolar, paramagnetic, and diamagnetic spin interactions. The experimental values of the geminal ^2^*J_HH_* and vicinal ^3^*J_HH_* coupling constants are close to each other and amount to 8.6, 9.7, and 8.6 Hz, which is in agreement with the calculated values (Figure 3). From the Karplus curve, which expresses the relationship between vicinal coupling constants and dihedral angles, these vicinal coupling constant values correspond to angles of approximately 30° (dihedral angle *H*-*C*^3^-*C*^2,4^-*H_e_*) and 160° (dihedral angle *H*-*C*^3^-*C*^2,4^-*H_a_*).

**Figure 4:**
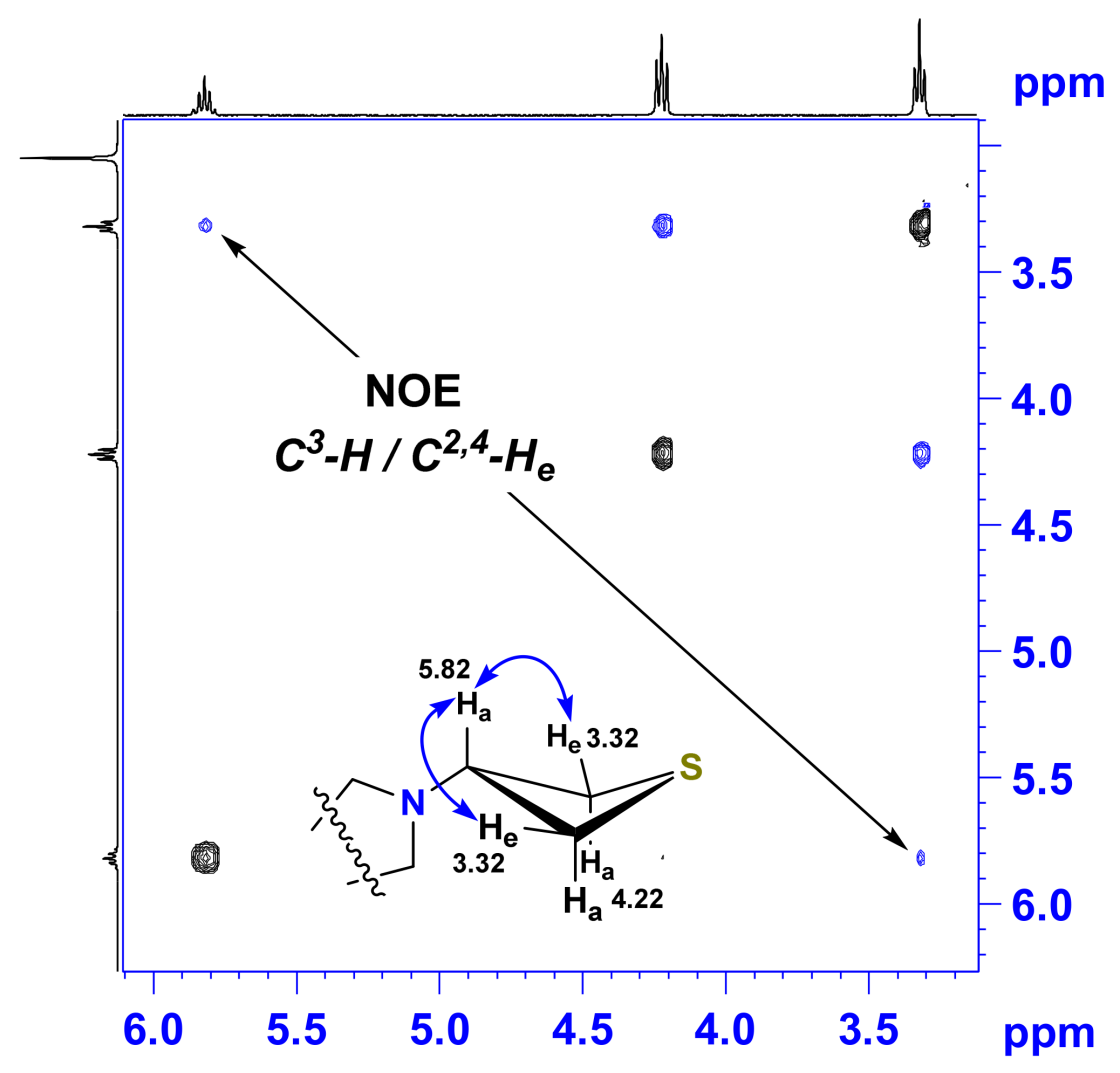
{^1^H,^1^H} NOESY spectrum (500 MHz, DMSO-*d*_6_) and scheme of NOE correlations for the thietane ring protons of compound **2**.

Furthermore, in the NOESY spectrum (Figure 4), the presence of a NOE correlation (nuclear Overhauser effect) between the signals of the spatially close *C*^3^-*H* proton and the *S*(*CH*)_2_ protons that exhibit the upfield triplet signal (*δ_H_* 3.32 ppm) unambiguously indicates the pseudoequatorial position of the latter.

In heteronuclear correlation experiments, cross-peak pairs characterize spin–spin coupling between protons and carbon or nitrogen nuclei through multiple bonds. Moreover, vicinal ^3^*J* constants exhibit a dependence on dihedral angles similar to the Karplus curve and can be used to discriminate between axial and equatorial protons. To further refine the spatial structure of the thietane ring, the calculated ^3^*J_CH_* and ^3^*J_NH_* values were compared with experimental data obtained from heteronuclear correlation spectra. The *J_CH_* and *J_NH_* coupling constants were calculated using the approach based on the recommendations of P. R. Rablen [35,36]. According to this methodology, the Fermi contact contribution to the coupling constants was calculated at the B3LYP/6-31G(d,p) level of theory with an extended basis set for hydrogen atoms (u+1s) (Table 1).

To determine the long-range *^n^J_CH_* coupling constants, the {^1^H,^13^C} LR-HSQMBC experiment, developed by Williamson and co-workers and optimized for observing a full range of weak long-range *^n^J_CH_* correlations, was used [37,38,39]. To determine the *^n^J_NH_* coupling constants, a gradient-selected {^1^H,^15^N} HMBC experiment [40], optimized for long-range coupling constants, without decoupling during acquisition, was employed.

**Figure 5:**
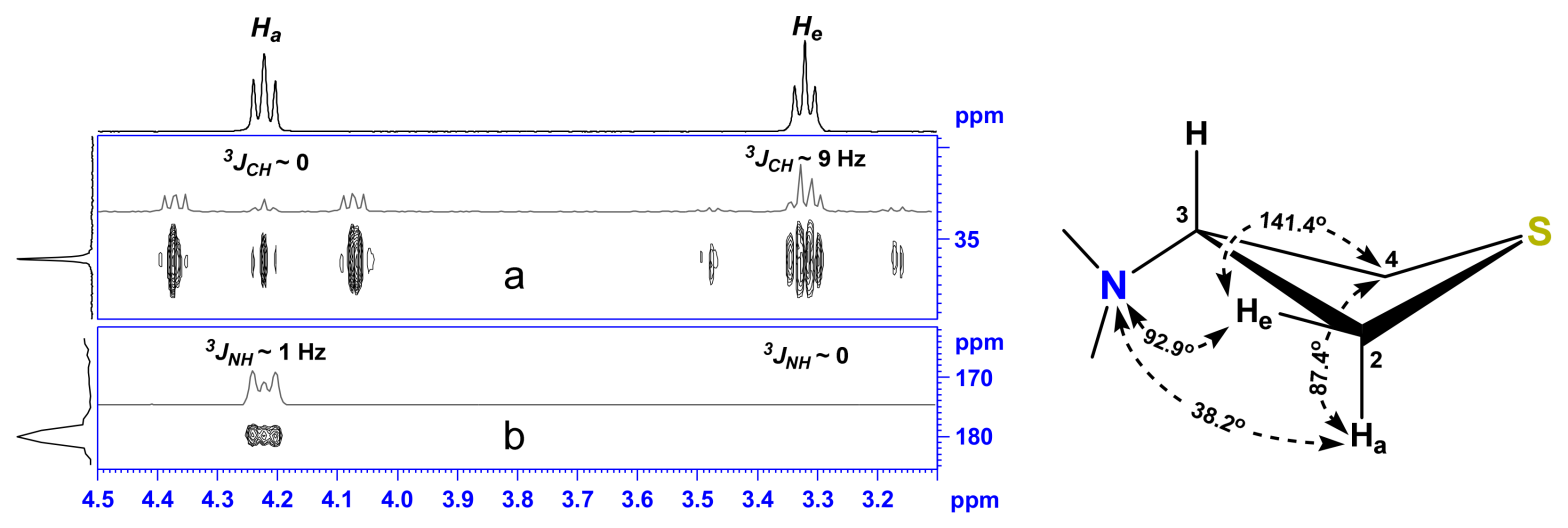
Selected regions of the {^1^H,^13^C} LR-HSQMBC (a) and {^1^H,^15^N} HMBC (b) spectra showing the methylene proton signals of compound **2**, with the calculated dihedral angles of the thietane ring.

In the {^1^H,^13^C} LR-HSQMBC spectrum (Figure 5), the absence of a cross-peak between the carbon signal of the *C*^2,4^ atoms and the downfield *S*(*CH*)_2_ proton signal indicates a minimal ^3^*J_CH_* value (dihedral angle close to 90°), which corresponds to an axial orientation. In the {^1^H,^15^N} HMBC spectrum, no cross-peak is observed between the signals of the *N*^7^-atom of the purine-2,6-dione moiety and the upfield *S*(*CH*)_2_ proton signal (calculated dihedral angle *N*^7^-*C*^3′^-*C*^2′,4′^-*H* of 92.9°), indicating their equatorial arrangement.

The absence of cross-peaks in the aforementioned experiments indicates minimal values of the corresponding coupling constants, enabling unambiguous determination of the spatial orientation of protons in the thietane ring. The comparison of experimental and calculated coupling constants (*J_HH_*, *J_CH_*, and *J_NH_*) presented in Table 1 shows good agreement and confirms the correctness of the proposed assignment of the axial and equatorial proton signals in the thietane ring.

### 2.3. Biological Study

In this work, the antiplatelet aggregation properties of 8-amino-substituted 1-isobutyl-3-methyl-3,7-dihydro-1*H*-purine-2,6-diones containing a thietane ring under study were investigated (Table 2).

As a result of biological activity study of the synthesized compounds, it was found that the expression of antiplatelet activity of compounds **6d**,**g**,**h** is comparable to that of acetylsalicylic acid. Compounds **6b**,**c**,**f** exhibited antiplatelet activity exceeding the activity of acetylsalicylic acid (MA 13.7 vs. 16.2–19.6 at *p* < 0.05). Compound **6b** demonstrated the highest antiplatelet activity, for which the maximum amplitude value in the ADP-induced platelet aggregation test exceeded the effect of acetylsalicylic acid by a factor of 1.4. 8-Morpholinyl-, 8-azepanyl-substituted 1-isobutylpurine-2,6-diones containing thietane or thietane oxide rings are active.

## 3. Discussion

The thietane ring in the key compound **3** is oxidized in good yields to thietane oxide (compound **4**) and thietane dioxide rings (compound **5**). In all thietane-containing 8-bromopurine-2,6-diones **3-5**, the bromine atom is readily replaced by amines to form 8-amino-substituted 1-isobutyl-3-methylpurine-2,6-diones containing thietane, thietane oxide, and thietane dioxide rings. Thus, the developed strategy allows the preparation of 1-isobutyl-3-methylpurine-2,6-diones by modifying both the thietane fragment and the amine residue at the *C*^8^ position.

**Figure 6:**
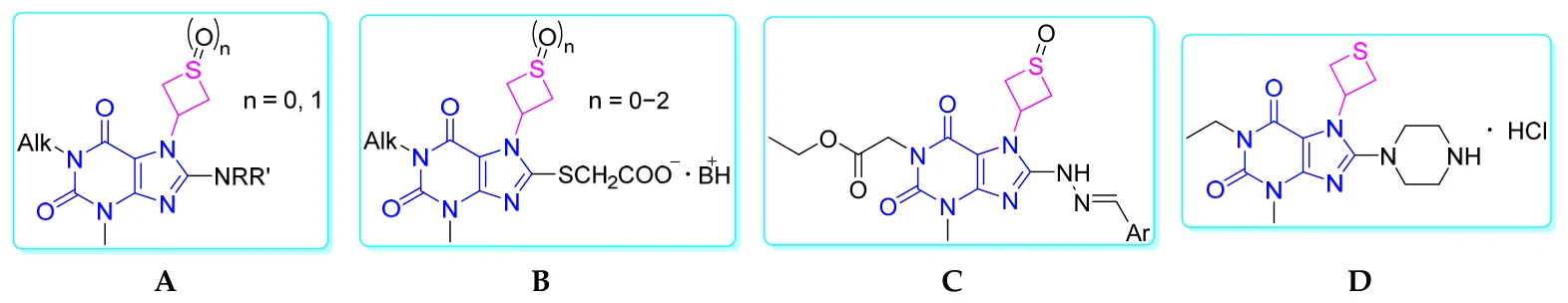
1,8-Disubstituted 3-methyl-3,7-dihydro-1*H*-purine-2,6-diones containing thietane rings described in the literature.

The literature described the antiaggregatory activity of 8-amino-substituted 1-alkyl-3-methyl-3,7-dihydro-1*H*-purine-2,6-diones containing a thietane ring (Figure 6A) against erythrocytes [19,20], as determined by viscometry [41]. The antiaggregatory activity of thietane-containing 2-[(1-alkyl-3-methyl-2,6-dioxo-2,3,6,7-tetrahydro-1*H*-purin-8-yl)sulfanyl]acetic acids and their salts (Figure 6B) against platelets is known [42,43]. Among them, compounds exhibiting activity at the level of acetylsalicylic acid were found [21]. Ethyl 2-[8-arylmethylidenehydrazinyl-3-methyl-2,6-dioxo-7-(1-oxothietan-3-yl)-2,3,6,7-tetrahydro-1*H*-purin-1-yl]acetates (Figure 6C) also exhibited activity at the level of acetylsalicylic acid [44].

The closest in structure to the synthesized compounds are 8-amino-substituted 1-alkyl-3-methyl-3,7-dihydro-1*H*-purine-2,6-diones containing thietane rings (Figure 6A). Recently, it was shown that a representative of this series, 1-ethyl-3-methyl-8-(piperazin-1-yl)-7-(thietan-3-yl)-3,7-dihydro-1*H*-purine-2,6-dione hydrochloride (Figure 6D), is undergoing Phase III clinical trials as antiplatelet agent that targets Glycoprotein IIb/IIIa inhibitors [45,46]. Therefore, the search for antiplatelet aggregation agents—glycoprotein IIb/IIIa inhibitors among 8-amino-substituted 1-isobutyl-3-methyl-3,7-dihydro-1*H*-purine-2,6-diones containing thietane ring is promising.

## 4. Materials and Methods

### 4.1. Materials and Instrumentations

IR spectra were recorded from KBr pellets on an InfraLUM FT-02 FTIR spectrometer (LUMEX, St. Petersburg, Russia). Instrument control, data acquisition, and spectral processing were performed using SpLUM software (version 1.02; LUMEX, Russia).

NMR spectra were recorded at 298 K on a Bruker Avance *III* pulsed spectrometer (Bruker BioSpin, Rheinstetten, Germany) at operating frequencies of 500.13 MHz (^1^H), 125.76 MHz (^13^C), and 50.68 MHz (^15^N) using a 5 mm broadband probe with Z-gradient (PABBO). Chemical shifts (ppm) are referenced to the solvent signal as internal standard (CDCl_3_: ^1^H NMR 7.26 ppm, ^13^C NMR 77.16 ppm, and DMSO-*d*_6_: ^1^H NMR 2.50 ppm, ^13^C NMR 39.52 ppm). ^15^N chemical shifts are referenced to external liquid ammonia. *J* values are given in Hz. ^13^C NMR spectra were interpreted using DEPT-90 and DEPT-135 experiments. Data acquisition was performed using Bruker TopSpin software (version 3.0.0; Bruker BioSpin, Rheinstetten, Germany).

High-resolution mass spectra (HRMS) were obtained using two different instruments depending on the compound. HRMS data for compounds **5**, **6a**, **6b**, and **6g** were acquired on an Agilent 6530C Accurate-Mass Q-TOF LC/MS system (Agilent Technologies, Santa Clara, CA, USA) equipped with an Agilent Dual AJS ESI source. Data acquisition was controlled by Agilent MassHunter Acquisition software (version v10.0; Agilent Technologies, Santa Clara, CA, USA). The instrument was operated in positive ion mode with the following parameters: gas temperature 300 °C, nebulizer pressure 7 psi, sheath gas temperature 325 °C, sheath gas flow 7 L/min, drying gas flow 4.0 L/min, capillary voltage 3500 V, nozzle voltage 2000 V. Mass spectra were acquired over the mass range (3000 *m*/*z*) in extended dynamic range (2 GHz) mode.

For all other compounds, HRMS data were acquired on an Orbitrap Elite mass spectrometer (Thermo Fisher Scientific, Waltham, MA, USA) equipped with a HESI ion source. Data acquisition was performed using XCalibur software (version 2.2 SP1.48; Thermo Fisher Scientific, Waltham, MA, USA). The instrument was operated in positive ion mode with the following parameters: spray voltage of 4 kV, capillary temperature of 275 °C, source heater temperature of 300 °C, resolution of 240000 (at *m*/*z* 400), and mass range of 110–2000 *m*/*z*. Theoretical *m*/*z* values were calculated using ChemDraw software (version 23.1.1.3 (64-bit); ChemOffice Suite, Revvity Signals Inc., Waltham, MA, USA).

NMR and mass spectra were processed and analyzed using Mnova software (version 12.0.0-20080; Mestrelab Research S.L., Santiago de Compostela, Spain).

Elemental analysis was performed on a Hekatech Euro3000 analyzer (Hekatech, Wegberg, Germany). Elemental analysis (C, H, N, and S) results were within ±0.3% of the calculated values. Melting points were determined on a Stuart SMP30 apparatus (Stuart, Stone, UK). The progress of reactions and the homogeneity of synthesized compounds were monitored by TLC on Sorbfil plates using BuOH–AcOH–H_2_O (4:2:1, *v*/*v*/*v*) for **4**, **6a**–**j**, and **6n**; and CHCl_3_–dioxane (9:1, *v*/*v*) for **5**, **6k**–**m**, and **6o**. Spots were visualized under UV light or in an I_2_ vapor chamber.

### 4.2. Synthesis and Characterization

All reactions were performed under an air atmosphere. Reagents were used as purchased without further purification unless otherwise stated. 8-Bromo-3-methyl-3,7-dihydro-1*H*-purine-2,6-dione (**1**) (Cat. No. DY34248; CAS 93703-24-3; purity ≥ 97%), and isobutyl bromide (Cat. No. DY1908; CAS 78-77-3; purity ≥ 99%) were purchased from DYC (Hangzhou, China). 2-(Chloromethyl)thiirane (CAS 3221-15-6) was purchased from Chem-Gold (Cat. No. 3221156; purity 95%). Potassium hydroxide (CAS 1310-58-3), potassium permanganate (CAS 7722-64-7), sodium thiosulfate (CAS 10102-17-7), hydrogen peroxide (30% aqueous solution, CAS 7722-84-1), piperidine (CAS 110-89-4), morpholine (CAS 110-91-8), azepane (CAS 111-49-9), benzylamine (CAS 100-46-9), ethanolamine (CAS 141-43-5), and all solvents (DMF, AcOH, CHCl_3_, EtOH, *i*-PrOH, MeOH, hexane, benzene, and AcCN) were purchased from commercial suppliers and used as received.

*8-Bromo-3-methyl-7-(thietan-3-yl)-3,7-dihydro-1H-purine-2,6-dione* (**2**). To a solution of compound **1** (24.50 g, 0.1 mol) and potassium hydroxide (6.73 g, 0.12 mol) in H_2_O (350 mL) at 50–60 °C, 2-(chloromethyl)thiirane (13.03 g, 0.12 mol) was added and stirred at 50–60 °C for 1 h. Then, to the reaction mixture, potassium hydroxide (3.37 g, 0.06 mol) and 2-(chloromethyl)thiirane (6.52 g, 0.06 mol) were added, followed by stirring at 50–60 °C for 1 h. The resulting precipitate was filtered off, rinsed with H_2_O and ether and dried. To the dry precipitate, 400 mL of chloroform was added, followed by boiling for 10 min, filtering, and cooling of the filtrate. The resulting precipitate was filtered off, rinsed with CHCl_3_ and dried. Yield 11.55 g (36%), white crystalline powder; mp 233–234 °C (*i-BuOH*); IR (KBr, *ν_max_*, cm^–1^) 1670–1725 (C=C, C=N, C=O), 2780–3250 (N-H); ^1^H NMR (500 MHz, DMSO-*d*_6_) *δ_H_* 3.05 (s, 3H, 3-CH_3_), 3.30–3.34 (m, 2H, S(CH)_2_), 4.20–4.24 (m, 2H, S(CH)_2_), 5.78–5.86 (m, 1H, 7-CH), 11.39 (s, 1H, 1-NH) ppm; ^13^C NMR (125 MHz, DMSO-*d*_6_) *δ_C_* 28.6 (3-CH_3_), 34.7 (S(CH_2_)_2_), 52.6 (7-CH), 109.0 (C^5^), 127.3 (C^8^), 150.2 (C^4^), 150.3 (C^2^), 153.8 (C^6^) ppm; {^1^H,^15^N} HSQC (50 MHz, DMSO-*d*_6_) *δ_N_* 153.6 (N^1^) ppm; {^1^H,^15^N} HMBC (50 MHz, DMSO-*d*_6_) *δ_N_* 113.5 (N^3^), 153.8 (N^1^), 179.9 (N^7^) ppm; HRMS (ESI) *m*/*z* found 316.9721, 318.9697, calcd for C_9_H_10_BrN_4_O_2_S^+^ [M + H]^+^ 316.9703, 318.9682; Elemental analysis: Calcd for C_9_H_9_BrN_4_O_2_S (%): C, 34.08; H, 2.86; N, 17.67; S, 10.11. Found (%): C, 34.02; H, 2.99; N, 17.50; S, 10.05.

*8-Bromo-1-isobutyl-3-methyl-7-(thietan-3-yl)-3,7-dihydro-1H-purine-2,6-dione* (**3**) was obtained according to our previously described procedure [21].

*8-Bromo-1-isobutyl-3-methyl-7-(1-oxothietan-3-yl)-3,7-dihydro-1H-purine-2,6-dione* (**4**). To a solution of compound **3** (1.87 g, 5 mmol) in AcOH (38 mL), 30% hydrogen peroxide solution (1.13 g, 10 mmol) was added. The reaction mixture was kept at r.t. for 1.5 h, then was neutralized with conc. ammonia solution at a temperature not exceeding 15 °C to pH 7. The resulting precipitate was filtered off, rinsed with H_2_O, and dried. Yield 1.62 g (83%), white crystalline powder; mp 191.5–192.7 °C (*EtOH*); IR (KBr, *ν_max_*, cm^–1^) 1049 (S=O), 1607, 1654, 1706, 1719 (C=C, C=N, C=O); ^1^H NMR (500 MHz, CDCl_3_) *δ_H_* 0.91 (d, 6H, (CH_3_)_2_), 2.10–2.18 (m, 1H, CH), 3.43–3.47 (m, 2H, S(CH)_2_), 3.54 (s, 3H, 3-CH_3_), 3.86 (d, 2H, 1-CH_2_), 4.30–4.34 (m, 2H, S(CH)_2_), 6.46–6.53 (m, 1H, 7-CH) ppm; ^13^C NMR (125 MHz, CDCl_3_) *δ_C_* 20.1 ((CH_3_)_2_), 27.2 (CH), 30.1 (3-CH_3_), 48.8 (1-CH_2_), 52.7 (7-CH), 55.3 (S(CH_2_)_2_), 108.7 (C^5^), 128.1 (C^8^), 149.9 (C^4^), 150.9 (C^2^), 154.3 (C^6^) ppm; HRMS (ESI) *m*/*z* found 389.0306, 391.0278, calcd for C_13_H_18_BrN_4_O_3_S^+^ [M + H]^+^ 389.0278, 391.0258; Elemental analysis: Calcd for C_13_H_17_BrN_4_O_3_S (%): C, 40.11; H, 4.40; N, 14.39; S, 8.24. Found (%): C, 40.25; H, 4.65; N, 14.22; S, 8.18.

*8-Bromo-1-isobutyl-3-methyl-7-(1,1-dioxothietan-3-yl)-3,7-dihydro-1H-purine-2,6-dione* (**5**). To a solution of compound **3** (5.60 g, 15 mmol) in AcOH (75 mL), a solution of potassium permanganate (4.74 g, 30 mmol) in H_2_O (75 mL) was added with stirring at r.t. for 1 h. The reaction mixture was stirred at 40 °C for 2 h, then decolorized with a solution of sodium thiosulfate (7.11 g, 45 mmol) in H_2_O (40 mL). The resulting precipitate was filtered off, rinsed with H_2_O, and dried. Yield 4.80 g (79%), white crystalline powder; mp 250.6–252.0 °C (*EtOH*); IR (KBr, *ν_max_*, cm^–1^) 1148, 1323 (SO_2_), 1593, 1618, 1656, 1702 (C=C, C=N, C=O); ^1^H NMR (500 MHz, CDCl_3_) *δ_H_* 0.90 (d, 6H, (CH_3_)_2_), 2.11–2.18 (m, 1H, CH), 3.33 (s, 3H, 3-CH_3_), 3.80 (d, 2H, 1-CH_2_), 4.35–4.39 (m, 2H, S(CH)_2_), 5.20–5.24 (m, 2H, S(CH)_2_), 5.58–5.62 (m, 1H, 7-CH) ppm; ^13^C NMR (125 MHz, CDCl_3_) *δ_C_* 20.0 ((CH_3_)_2_), 27.1 (CH), 30.0 (3-CH_3_), 48.6 (1-CH_2_), 40.4 (7-CH), 70.1 (S(CH_2_)_2_), 108.5 (C^5^), 129.1 (C^8^), 149.7 (C^4^), 150.9 (C^2^), 154.3 (C^6^) ppm; HRMS (ESI) *m*/*z* found 405.0229, 407.0216, calcd for C_13_H_18_BrN_4_O_4_S^+^ [M + H]^+^ 405.0227, 407.0207; Elemental analysis: Calcd for C_13_H_17_BrN_4_O_4_S (%): C, 38.53; H, 4.23; N, 13.82; S, 7.91. Found (%): C, 38.61, H 4.49, N 13.65, S 7.78.

**General Procedure for the Synthesis of 8-Amino-substituted 1-isobutyl-3-methyl-7-(thietan-3-yl)-3,7-dihydro-1*H*-purine-2,6-diones (6a–e).** To a solution of compound **3** (1.12 g, 3 mmol) in EtOH (25 mL), the corresponding amine (9 mmol in the case of the synthesis of compound **6d**—12 mmol of benzylamine) was added, and the reaction mixture was boiled for 6–7 h. Then, it was cooled, and the resulting precipitate was filtered off, rinsed with H_2_O, and dried.

*1-Isobutyl-3-methyl-8-(piperidin-1-yl)-7-(thietan-3-yl)-3,7-dihydro-1H-purine-2,6-dione* (**6a**) white crystalline powder; yield 83%; mp 152.6–153.6 °C (*i-PrOH*); IR (KBr, *ν_max_*, cm^–1^) 1611, 1651, 1662, 1691 (C=C, C=N, C=O); ^1^H NMR (500 MHz, CDCl_3_) *δ_H_* 0.91 (d, *J* = 6.7 Hz, 6H, (CH_3_)_2_), 1.63–1.74 (m, 6H, (CH_2_)_3_), 2.15–2.22 (m, 1H, CH), 3.12 (t, *J* = 5.05 Hz, 4H, 8-N(CH_2_)_2_), 3.22–3.25 (m, 2H, S(CH)_2_), 3.49 (s, 3H, 3-CH_3_), 3.90 (d, *J* = 7.4 Hz, 2H, 1-CH_2_), 4.31–4.35 (m, 2H, S(CH)_2_), 5.40–5.48 (m, 1H, 7-CH) ppm; ^13^C NMR (125 MHz, CDCl_3_) *δ_C_* 20.1 ((CH_3_)_2_), 24.0 ((CH_2_)_2_CH_2_), 25.4 (CH_2_(CH_2_)_2_), 27.2 (CH), 29.8 (3-CH_3_), 35.8 (S(CH_2_)_2_), 48.2 (1-CH_2_), 51.7 (7-CH), 52.6 (8-N(CH_2_)_2_), 105.1 (C^5^), 148.9 (C^4^), 151.8 (C^2^), 154.8 (C^6^), 157.0 (C^8^) ppm; HRMS (ESI) *m*/*z* found 378.1975, calcd for C_18_H_28_N_5_O_2_S^+^ [M + H]^+^ 378.1959; Elemental analysis: Calcd for C_18_H_27_N_5_O_2_S (%): C, 57.27; H, 7.21; N, 18.55; S, 8.49. Found (%): C, 57.11; H, 7.46; N, 18.48; S, 8.33.

*1-Isobutyl-3-methyl-8-(morpholin-4-yl)-7-(thietan-3-yl)-3,7-dihydro-1H-purine-2,6-dione* (**6b**) white crystalline powder; yield 64%; mp 160.5–161.2 °C (*EtOH*); IR (KBr, *ν_max_*, cm^–1^) 1609, 1648, 1656, 1698 (C=C, C=N, C=O); ^1^H NMR (500 MHz, CDCl_3_) *δ_H_* 0.90 (d, *J* = 5.9 Hz, 6H, (CH_3_)_2_), 2.12–2.21 (m, 1H, CH), 3.11–3.26 (m, 6H, 8-N(CH_2_)_2_, S(CH)_2_), 3.48 (s, 3H, 3-CH_3_), 3.78–3.92 (m, 6H, O(CH_2_)_2_, 1-CH_2_), 4.29–4.37 (m, 2H, S(CH)_2_), 5.44–5.54 (m, 1H, 7-CH) ppm; ^13^C NMR (125 MHz, CDCl_3_) *δ_C_* 20.0 ((CH_3_)_2_), 27.2 (CH), 29.8 (3-CH_3_), 35.7 (S(CH_2_)_2_), 48.3 (1-CH_2_), 51.3 (7-CH), 51.6 (8-N(CH_2_)_2_), 66.2 (O(CH_2_)_2_), 105.5 (C^5^), 148.6 (C^4^), 151.7 (C^2^), 154.9 (C^6^), 155.5 (C^8^) ppm; HRMS (ESI) *m*/*z* found 380.1757, calcd for C_17_H_26_N_5_O_3_S^+^ [M + H]^+^ 380.1751; Elemental analysis: Calcd for C_17_H_25_N_5_O_3_S (%): C, 53.81; H, 6.64; N, 18.46; S, 8.45. Found (%): C, 53.75; H, 6.89; N, 18.29; S, 8.19.

*8-(Azepan-1-yl)-1-isobutyl-3-methyl-7-(thietan-3-yl)-3,7-dihydro-1H-purine-2,6-dione* (**6c**) white crystalline powder; yield 74%; mp 116.0–117.5 °C (hexane); IR (KBr, *ν_max_*, cm^–1^) 1616, 1645, 1652, 1698 (C=C, C=N, C=O); ^1^H NMR (500 MHz, CDCl_3_) *δ_H_* 0.89 (d, *J* = 6.7 Hz, 6H, (CH_3_)_2_), 1.64–1.69 (m, 4H, (CH_2_)_2_), 1.80–1.86 (m, 4H, 2CH_2_), 2.12–2.21 (m, 1H, CH), 3.16–3.19 (m, 2H, S(CH)_2_), 3.40 (t, *J* = 5.9 Hz, 4H, 8-N(CH_2_)_2_), 3.51 (s, 3H, 3-CH_3_), 3.86 (d, *J* = 7.5 Hz, 2H, 1-CH_2_), 4.30–4.34 (m, 2H, S(CH)_2_), 5.32–5.40 (m, 1H, 7-CH) ppm; ^13^C NMR (125 MHz, CDCl_3_) *δ_C_* 20.0 ((CH_3_)_2_), 27.2 (CH), 27.3 ((CH_2_)_2_), 28.6 (2CH_2_), 29.7 (3-CH_3_), 36.0 (S(CH_2_)_2_), 48.1 (1-CH_2_), 52.4 (7-CH), 53.6 (8-N(CH_2_)_2_), 104.4 (C^5^), 149.4 (C^4^), 151.8 (C^2^), 154.7 (C^6^), 158.4 (C^8^) ppm; HRMS (ESI) *m*/*z* found 392.2147, calcd for C_19_H_30_N_5_O_2_S^+^ [M + H]^+^ 392.2115; Elemental analysis: Calcd for C_19_H_29_N_5_O_2_S (%): C, 58.29; H, 7.47; N, 17.89; S, 8.19. Found (%): C, 58.53; H, 7.52; N, 17.72; S, 8.03.

*8-(Benzylamino)-1-isobutyl-3-methyl-7-(thietan-3-yl)-3,7-dihydro-1H-purine-2,6-dione* (**6d**) white crystalline powder; yield 68%; mp 156.2–157.6 °C (*i-PrOH*); IR (KBr, *ν_max_*, cm^–1^) 1615, 1647, 1698 (C=C, C=N, C=O), 3230–3380 (N-H); ^1^H NMR (500 MHz, CDCl_3_) *δ_H_* 0.91 (d, *J* = 6.7 Hz, 6H, (CH_3_)_2_), 2.12–2.18 (m, 1H, CH), 3.51 (s, 3H, 3-CH_3_), 3.60–3.64 (m, 2H, S(CH)_2_), 3.81–3.87 (m, 4H, 1-CH_2_, S(CH)_2_), 4.75 (d, *J* = 5.5 Hz, 2H, 8-NCH_2_), 5.94–6.00 (m, 1H, 7-CH), 6.06 (br.s, 1H, 8-NH), 7.28–7.45 (m, 5H, C_6_H_5_) ppm; ^13^C NMR (125 MHz, CDCl_3_) *δ_C_* 20.2 ((CH_3_)_2_), 27.3 (CH), 29.7 (3-CH_3_), 33.2 (S(CH_2_)_2_), 47.8 (1-CH_2_), 48.0 (8-NCH_2_), 50.7 (7-CH), 127.8, 127.9, 128.9, 138.1 (C_6_H_5_), 102.6 (C^5^), 149.0 (C^4^), 151.7 (C^2^), 152.5 (C^6^), 154.2 (C^8^) ppm; HRMS (ESI) *m*/*z* found 400.1831, calcd for C_20_H_26_N_5_O_2_S^+^ [M + H]^+^ 400.1802; Elemental analysis: Calcd for C_20_H_25_N_5_O_2_S (%): C, 60.13; H, 6.31; N, 17.53; S, 8.02. Found (%): C, 60.07; H, 6.56; N, 17.36; S, 7.86.

*8-[(2-Hydroxyethyl)amino]-1-isobutyl-3-methyl-7-(thietan-3-yl)-3,7-dihydro-1H-purine-2,6-dione* (**6e**) white crystalline powder; yield 65%; mp 157.2–159.6 °C (benzene); IR (KBr, *ν_max_*, cm^–1^) 1609, 1638, 1646, 1696 (C=C, C=N, C=O), 3250–3480 (N-H, O-H); ^1^H NMR (500 MHz, DMSO-*d*_6_) *δ_H_* 0.83 (d, *J* = 6.6 Hz, 6H, (CH_3_)_2_), 2.00–2.08 (m, 1H, CH), 3.15–3.18 (m, 2H, S(CH)_2_), 3.30–3.38 (m, 5H, 3-CH_3_, 8-NCH_2_), 3.52–3.56 (m, 2H, OCH_2_), 3.71 (d, *J* = 7.3 Hz, 2H, 1-CH_2_), 4.12–4.16 (m, 2H, S(CH)_2_), 4.73 (t, *J* = 5.4 Hz, 1H, OH), 5.84–5.91 (m, 1H, 7-CH), 7.34 (br.s, 1H, 8-NH) ppm; ^13^C NMR (125 MHz, DMSO-*d*_6_) *δ_C_* 20.5 ((CH_3_)_2_), 27.3 (CH), 29.8 (3-CH_3_), 36.4 (S(CH_2_)_2_), 45.8 (8-NCH_2_), 47.7 (1-CH_2_), 49.3 (7-CH), 60.0 (OCH_2_), 102.4 (C^5^), 150.4 (C^2^), 151.4 (C^6^), 153.4 (C^8^) ppm; HRMS (ESI) *m*/*z* found 354.1619, calcd for C_15_H_24_N_5_O_3_S^+^ [M + H]^+^ 354.1595; Elemental analysis: Calcd for C_15_H_23_N_5_O_3_S (%): C, 50.97; H, 6.56; N, 19.82; S, 9.07. Found (%): C, 51.21; H, 6.61; N, 19.65; S, 8.91.

**General Procedure for the Synthesis of 8-Amino-substituted 1-isobutyl-3-methyl-7-(1-oxothietan-3-yl)-3,7-dihydro-1*H*-purine-2,6-diones (6f–j).** To a solution of compound **4** (1.17 g, 3 mmol) in EtOH (40 mL), the corresponding amine (9 mmol) was added, and the reaction mixture was boiled for 5–8 h. Then, it was cooled, and the resulting precipitate was filtered off, rinsed with H_2_O, and dried.

*1-Isobutyl-3-methyl-8-(piperidin-1-yl)-7-(1-oxothietan-3-yl)-3,7-dihydro-1H-purine-2,6-dione* (**6f**) white crystalline powder; yield 56%; mp 221.3–222.4 °C (*MeOH*); IR (KBr, *ν_max_*, cm^–1^) 1063 (S=O), 1612, 1652, 1660, 1688, 1699 (C=C, C=N, C=O); ^1^H NMR (500 MHz, CDCl_3_) *δ_H_* 0.89 (d, *J* = 5.7 Hz, 6H, (CH_3_)_2_), 1.58–1.77 (m, 6H, (CH_2_)_3_), 2.08–2.18 (m, 1H, CH), 3.12 (br.s, 4H, 8-N(CH_2_)_2_), 3.34–3.42 (m, 2H, S(CH)_2_), 3.50 (s, 3H, 3-CH_3_), 3.82 (d, *J* = 6.5 Hz, 2H, 1-CH_2_), 4.21–4.29 (m, 2H, S(CH)_2_), 6.02–6.09 (m, 1H, 7-CH) ppm; ^13^C NMR (125 MHz, CDCl_3_) *δ_C_* 20.1 ((CH_3_)_2_), 23.9 ((CH_2_)_2_CH_2_), 25.3 ((CH_2_)_2_CH_2_), 27.2 (CH), 29.8 (3-CH_3_), 48.3 (1-CH_2_), 50.5 (7-CH), 52.6 (8-N(CH_2_)_2_), 55.9 (S(CH_2_)_2_), 104.3 (C^5^), 149.7 (C^4^), 151.6 (C^2^), 154.9 (C^6^), 157.6 (C^8^) ppm; HRMS (ESI) *m*/*z* found 394.1927, calcd for C_18_H_28_N_5_O_3_S^+^ [M + H]^+^ 394.1908; Elemental analysis: Calcd for C_18_H_27_N_5_O_3_S (%): C, 54.94; H, 6.92; N, 17.80; S, 8.15. Found (%): C, 54.78; H, 7.17; N, 17.63; S, 8.09.

*1-Isobutyl-3-methyl-8-(morpholin-4-yl)-7-(1-oxothietan-3-yl)-3,7-dihydro-1H-purine-2,6-dione* (**6g**) white crystalline powder; yield 70%; mp 213.2–214.5 °C (*MeOH*); IR (KBr, *ν_max_*, cm^–1^) 1063 (S=O), 1614, 1643, 1668, 1692, 1699 (C=C, C=N, C=O); ^1^H NMR (500 MHz, CDCl_3_) *δ_H_* 0.90 (d, *J* = 6.4 Hz, 6H, (CH_3_)_2_), 2.09–2.17 (m, 1H, CH), 3.18 (br.s, 4H, 8-N(CH_2_)_2_), 3.33–3.40 (m, 2H, S(CH)_2_), 3.50 (s, 3H, 3-CH_3_), 3.79–3.89 (m, 6H, O(CH_2_)_2_, 1-CH_2_), 4.23–4.30 (m, 2H, S(CH)_2_), 6.09–6.16 (m, 1H, 7-CH) ppm; ^13^C NMR (125 MHz, CDCl_3_) *δ_C_* 20.1 ((CH_3_)_2_), 27.2 (CH), 29.9 (3-CH_3_), 48.4 (1-CH_2_), 50.3 (7-CH), 51.6 (8-N(CH_2_)_2_), 55.8 (S(CH_2_)_2_), 66.1 (O(CH_2_)_2_), 104.7 (C^5^), 149.4 (C^4^), 151.5 (C^2^), 155.0 (C^6^), 156.2 (C^8^) ppm; HRMS (ESI) *m*/*z* found 396.1703, calcd for C_17_H_26_N_5_O_4_S^+^ [M + H]^+^ 396.1701; Elemental analysis: Calcd for C_17_H_25_N_5_O_4_S (%) C, 51.63; H, 6.37; N, 17.71; S, 8.11. Found (%) C, 51.48; H, 6.51; N, 17.55; S, 8.01.

*8-(Azepan-1-yl)-1-isobutyl-3-methyl-7-(1-oxothietan-3-yl)-3,7-dihydro-1H-purine-2,6-dione* (**6h**) white crystalline powder; yield 64%; mp 212.8–213.8 °C (*i-PrOH*); IR (KBr, *ν_max_*, cm^–1^) 1063 (S=O), 1614, 1649, 1662, 1695 (C=C, C=N, C=O); ^1^H NMR (500 MHz, CDCl_3_) *δ_H_* 0.89 (d, *J* = 6.6 Hz, 6H, (CH_3_)_2_), 1.66 (br.s, 4H, (CH_2_)_2_), 1.84 (br.s, 4H, 2CH_2_), 2.09–2.15 (m, 1H, CH), 3.32–3.37 (m, 2H, S(CH)_2_), 3.45 (t, *J* = 5.8 Hz, 4H, 8-N(CH_2_)_2_), 3.48 (s, 3H, 3-CH_3_), 3.80 (d, *J* = 7.4 Hz, 2H, 1-CH_2_), 4.23–4.28 (m, 2H, S(CH)_2_), 5.97–6.04 (m, 1H, 7-CH) ppm; ^13^C NMR (125 MHz, CDCl_3_) *δ_C_* 20.1 ((CH_3_)_2_), 27.2 (CH), 27.4 ((CH_2_)_2_), 28.5 (2-CH_2_), 29.8 (3-CH_3_), 48.2 (1-CH_2_), 51.3 (7-CH), 53.4 (8-N(CH_2_)_2_), 56.1 (S(CH_2_)_2_), 103.7 (C^5^), 150.2 (C^4^), 151.6 (C^2^), 154.7 (C^6^), 159.0 (C^8^) ppm; HRMS (ESI) *m*/*z* found 408.2082, calcd for C_19_H_30_N_5_O_3_S^+^ [M + H]^+^ 408.2064; Elemental analysis: Calcd for C_19_H_29_N_5_O_3_S (%): C, 56.00; H, 7.17; N, 17.19; S, 7.87. Found (%): C, 55.84; H, 7.32; N, 17.02; S, 7.61.

*8-(Benzylamino)-1-isobutyl-3-methyl-7-(1-oxothietan-3-yl)-3,7-dihydro-1H-purine-2,6-dione* (**6i**) white crystalline powder; yield 35%; mp 239.2–240.4 °C (*MeOH*); IR (KBr, *ν_max_*, cm^–1^) 1045 (S=O), 1620, 1648, 1695 (C=C, C=N, C=O), 3250–3360 (N-H); ^1^H NMR (500 MHz, CDCl_3_) *δ_H_* 0.92 (d, *J* = 6.8 Hz, 6H, (CH_3_)_2_), 2.12–2.20 (m, 1H, CH), 3.16–3.21 (m, 2H, S(CH)_2_), 3.52 (s, 3H, 3-CH_3_), 3.83 (d, *J* = 7.5 Hz, 2H, 1-CH_2_), 4.23–4.28 (m, 2H, S(CH)_2_), 4.55 (d, *J* = 5.2 Hz, 2H, 8-NCH_2_), 6.31–6.38 (m, 1H, 7-CH), 7.17 (t, *J* = 5.2 Hz, 1H, 8-NH), 7.28–7.39 (m, 5H, C_6_H_5_) ppm; ^13^C NMR (125 MHz, CDCl_3_) *δ_C_* 20.1 ((CH_3_)_2_), 27.3 (CH), 29.8 (3-CH_3_), 47.6 (1-CH_2_), 48.2 (8-NCH_2_), 48.4 (7-CH), 56.6 (S(CH_2_)_2_), 102.6 (C^5^), 127.8, 128.6, 128.6, 138.2 (C_6_H_5_), 151.2 (C^4^), 151.7 (C^2^), 153.1 (C^6^), 153.9 (C^8^) ppm; HRMS (ESI) *m*/*z* found 416.1776, calcd for C_20_H_26_N_5_O_3_S^+^ [M + H]^+^ 416.1751; Elemental analysis: Calcd for C_20_H_25_N_5_O_3_S (%): C, 57.81; H, 6.06; N, 16.86; S, 7.72. Found (%): C, 57.65; H, 6.21; N, 16.69; S, 7.66.

*8-[(2-Hydroxyethyl)amino]-1-isobutyl-3-methyl-7-(1-oxothietan-3-yl)-3,7-dihydro-1H-purine-2,6-dione* (**6j**) white crystalline powder; yield 53%; mp 249.3–250.3 °C (*i-PrOH*); IR (KBr, *ν_max_*, cm^–1^) 1074 (S=O), 1617, 1655, 1695 (C=C, C=N, C=O), 3180–3400 (N-H, O-H); ^1^H NMR (500 MHz, DMSO-*d*_6_) *δ_H_* 0.81 (d, *J* = 6.5 Hz, 6H, (CH_3_)_2_), 1.98–2.06 (m, 1H, CH), 3.26–3.37 (m, 7H, S(CH)_2_, 3-CH_3_, 8-NCH_2_), 3.52–3.56 (m, 2H, OCH_2_), 3.67 (d, *J* = 7.0 Hz, 2H, 1-CH_2_), 4.02–4.08 (m, 2H, S(CH)_2_), 4.75 (t, *J* = 5.2 Hz, 1H, OH), 6.20–6.27 (m, 1H, 7-CH), 7.47 (br.s, 1H, 8-NH) ppm; ^13^C NMR (125 MHz, DMSO-*d*_6_) *δ_C_* 20.4 ((CH_3_)_2_), 27.2 (CH), 29.9 (3-CH_3_), 45.9 (8-NCH_2_), 47.8 (1-CH_2_), 48.4 (7-CH), 56.2 (S(CH_2_)_2_), 60.0 (OCH_2_), 101.8 (C^5^), 151.0 (C^4^), 151.3 (C^2^), 153.4 (C^6^), 153.7 (C^8^) ppm; HRMS (ESI) *m*/*z* found 370.1562, calcd for C_15_H_24_N_5_O_4_S^+^ [M + H]^+^ 370.1544; Elemental analysis: Calcd for C_15_H_23_N_5_O_4_S (%): C, 48.77; H, 6.28; N, 18.96; S, 8.68. Found (%): C, 48.61; H, 6.43; N, 18.79; S, 8.52.

**General Procedure for the Synthesis of 8-Amino-substituted 1-isobutyl-3-methyl-7-(1,1-dioxothietan-3-yl)-3,7-dihydro-1*H*-purine-2,6-diones (6k–o).** To a solution of compound **5** (1.22 g, 3 mmol) in EtOH (40 mL), the corresponding amine (9 mmol) was added, and the reaction mixture was boiled for 1.5–3.5 h. Then, it was cooled, and the resulting precipitate was filtered off, rinsed with water, and dried.

*1-Isobutyl-3-methyl-8-(piperidin-1-yl)-7-(1,1-dioxothietan-3-yl)-3,7-dihydro-1H-purine-2,6-dione* (**6k**) white crystalline powder; yield 82%; mp 223.4–224.3 °C (*i-PrOH*); IR (KBr, *ν_max_*, cm^–1^) 1149, 1317 (SO_2_), 1616, 1649, 1656, 1666, 1695 (C=C, C=N, C=O); ^1^H NMR (500 MHz, CDCl_3_) *δ_H_* 0.82 (d, *J* = 6.6 Hz, 6H, (CH_3_)_2_), 1.55–1.69 (m, 6H, (CH_2_)_3_), 2.04–2.12 (m, 1H, CH), 3.07 (t, *J* = 5.15 Hz, 4H, 8-N(CH_2_)_2_), 3.42 (s, 3H, 3-CH_3_), 3.79 (d, *J* = 7.5 Hz, 2H, 1-CH_2_), 4.19–4.23 (m, 2H, S(CH)_2_), 4.99–5.12 (m, 3H, S(CH)_2_, 7-CH) ppm; ^13^C NMR (125 MHz, CDCl_3_) *δ_C_* 20.0 ((CH_3_)_2_), 23.8 ((CH_2_)_2_CH_2_), 25.3 ((CH_2_)_2_CH_2_), 27.1 (CH), 29.8 (3-CH_3_), 38.6 (7-CH), 48.2 (1-CH_2_), 52.6 (8-N(CH_2_)_2_), 70.0 (S(CH_2_)_2_), 104.1 (C^5^), 149.5 (C^4^), 151.6 (C^2^), 154.9 (C^6^), 159.0 (C^8^) ppm; HRMS (ESI) *m*/*z* found 410.1874, calcd for C_18_H_28_N_5_O_4_S^+^ [M + H]^+^ 410.1857; Elemental analysis: Calcd for C_18_H_27_N_5_O_4_S (%): C, 52.79; H, 6.65; N, 17.10; S, 7.83. Found (%): C, 52.73; H, 6.98; N, 17.03; S, 7.57.

*1-Isobutyl-3-methyl-8-(morpholin-4-yl)-7-(1,1-dioxothietan-3-yl)-3,7-dihydro-1H-purine-2,6-dione* (**6l**) white crystalline powder; yield 83%; mp 244.6–245.5 °C (*i-PrOH*); IR (KBr, *ν_max_*, cm^–1^) 1139, 1320 (SO_2_), 1612, 1657, 1665, 1698, 1705 (C=C, C=N, C=O); ^1^H NMR (500 MHz, DMSO-*d*_6_) *δ_H_* 0.83 (d, *J* = 6.6 Hz, 6H, (CH_3_)_2_), 2.01–2.08 (m, 1H, CH), 3.15 (br.s, 4H, 8-N(CH_2_)_2_), 3.38 (s, 3H, 3-CH_3_), 3.69–3.78 (m, 6H, 1-CH_2_, O(CH_2_)_2_), 4.55–4.59 (m, 2H, S(CH)_2_), 4.88–4.93 (m, 2H, S(CH)_2_), 5.05–5.12 (m, 1H, 7-CH) ppm; ^13^C NMR (125 MHz, DMSO-*d*_6_) *δ_C_* 20.4 ((CH_3_)_2_), 27.2 (CH), 30.1 (3-CH_3_), 38.6 (7-CH), 48.0 (1-CH_2_), 51.6 (8-N(CH_2_)_2_), 66.0 (O(CH_2_)_2_), 70.3 (S(CH_2_)_2_), 104.4 (C^5^), 149.2 (C^4^), 151.3 (C^2^), 154.7 (C^6^), 158.2 (C^8^) ppm; HRMS (ESI) *m*/*z* found 412.1668, calcd for C_17_H_26_N_5_O_5_S^+^ [M + H]^+^ 412.1650; Elemental analysis: Calcd for C_17_H_25_N_5_O_5_S (%): C, 49.62; H, 6.12; N, 17.02; S, 7.79. Found (%): C, 49.56; H, 6.37; N, 16.85; S, 7.63.

*8-(Azepan-1-yl)-1-isobutyl-3-methyl-7-(1,1-dioxothietan-3-yl)-3,7-dihydro-1H-purine-2,6-dione* (**6m**) white crystalline powder; yield 71%; mp 199.2–200.4 °C (*i-PrOH*); IR (KBr, *ν_max_*, cm^–1^) 1150, 1311 (SO_2_), 1615, 1655, 1661, 1697 (C=C, C=N, C=O); ^1^H NMR (500 MHz, DMSO-*d*_6_) *δ_H_* 0.83 (d, *J* = 6.7 Hz, 6H, (CH_3_)_2_), 1.57 (br.s, 4H, (CH_2_)_2_), 1.79 (br.s, 4H, 2CH_2_), 2.00–2.08 (m, 1H, CH), 3.35 (s, 3H, 3-CH_3_), 3.45 (t, *J* = 5.6 Hz, 4H, 8-N(CH_2_)_2_), 3.70 (d, *J* = 7.4 Hz, 2H, 1-CH_2_), 4.44–4.48 (m, 2H, S(CH)_2_), 4.86–4.97 (m, 3H, S(CH)_2_, 7-CH) ppm; ^13^C NMR (125 MHz, DMSO-*d*_6_) *δ_C_* 20.4 ((CH_3_)_2_), 27.2 (CH), 27.4 (CH_2_)_2_, 28.4 (2CH_2_), 30.0 (3-CH_3_), 39.9 (7-CH), 47.9 (1-CH_2_), 52.7 (8-N(CH_2_)_2_), 70.1 (S(CH_2_)_2_), 103.1 (C^5^), 150.3 (C^4^), 151.4 (C^2^), 154.4 (C^6^), 160.9 (C^8^) ppm; HRMS (ESI) *m*/*z* found 424.2033, calcd for C_19_H_30_N_5_O_4_S^+^ [M + H]^+^ 424.2014; Elemental analysis: Calcd for C_19_H_29_N_5_O_4_S (%): C, 53.88; H, 6.90; N, 16.54; S, 7.57. Found (%): C, 53.62; H, 7.15; N, 16.37; S, 7.51.

*8-(Benzylamino)-1-isobutyl-3-methyl-7-(1,1-dioxothietan-3-yl)-3,7-dihydro-1H-purine-2,6-dione* (**6n**) white crystalline powder; yield 83%; mp 244.8–246.3 °C (*AcCN*); IR (KBr, *ν_max_*, cm^–1^) 1131, 1324 (SO_2_), 1623, 1645, 1658, 1664, 1693 (C=C, C=N, C=O), 3320–3380 (N-H); ^1^H NMR (500 MHz, DMSO-*d*_6_) *δ_H_* 0.82 (d, *J* = 6.4 Hz, 6H, (CH_3_)_2_), 2.00–2.07 (m, 1H, CH), 3.33 (s, 3H, 3-CH_3_), 3.69 (d, *J* = 6.9 Hz, 2H, 1-CH_2_), 4.32–4.36 (m, 2H, S(CH)_2_), 4.52 (d, *J* = 4.6 Hz, 2H, 8-NCH_2_), 4.92–4.96 (m, 2H, S(CH)_2_), 5.28–5.36 (m, 1H, 7-CH), 7.24–7.39 (m, 5H, C_6_H_5_), 8.00 (br.s, 1H, 8-NH) ppm; ^13^C NMR (125 MHz, DMSO-*d*_6_) *δ_C_* 20.4 ((CH_3_)_2_), 27.2 (CH), 29.9 (3-CH_3_), 36.6 (7-CH), 46.8 (8-NCH_2_), 47.8 (1-CH_2_), 70.3 (S(CH_2_)_2_), 127.6, 128.3, 128.8, 139.3 (C_6_H_5_), 101.9 (C^5^), 150.8 (C^4^), 151.3 (C^2^), 153.6 (C^6^), 155.3 (C^8^) ppm; HRMS (ESI) *m*/*z* found 432.1718, calcd for C_20_H_26_N_5_O_4_S^+^ [M + H]^+^ 432.1701; Elemental analysis: Calcd for C_20_H_25_N_5_O_4_S (%): C, 55.67; H, 5.84; N, 16.23; S, 7.43. Found (%): C, 55.61; H, 5.89; N, 16.36; S, 7.17.

*8-[(2-Hydroxyethyl)amino]-1-isobutyl-3-methyl-7-(1,1-dioxothietan-3-yl)-3,7-dihydro-1H-purine-2,6-dione* (**6o**) white crystalline powder; yield 39%; mp 246.2–246.9 °C (*i-PrOH*); IR (KBr, *ν_max_*, cm^–1^) 1131, 1305 (SO_2_), 1616, 1653, 1661, 1696 (C=C, C=N, C=O), 3320–3580 (N-H, O-H); ^1^H NMR (500 MHz, DMSO-*d*_6_) *δ_H_* 0.82 (d, *J* = 6.6 Hz, 6H, (CH_3_)_2_), 1.99–2.07 (m, 1H, CH), 3.32 (s, 3H, 3-CH_3_), 3.36–3.39 (m, 2H, 8-NCH_2_), 3.53–3.56 (m, 2H, OCH_2_), 3.68 (d, *J* = 7.3 Hz, 2H, 1-CH_2_), 4.29–4.33 (m, 2H, S(CH)_2_), 4.75 (t, *J* = 6.5 Hz, 1H, OH), 4.90–4.94 (m, 2H, S(CH)_2_), 5.25–5.32 (m, 1H, 7-CH), 7.54 (t, *J* = 5.0 Hz, 1H, 8-NH) ppm; ^13^C NMR (125 MHz, DMSO-*d*_6_) *δ_C_* 20.4 ((CH_3_)_2_), 27.2 (CH), 29.9 (3-CH_3_), 36.6 (7-CH), 45.9 (8-NCH_2_), 47.7 (1-CH_2_), 59.9 (OCH_2_), 70.3 (S(CH_2_)_2_), 101.7 (C^5^), 151.0 (C^4^), 151.3 (C^2^), 153.5 (C^6^), 155.5 (C^8^) ppm; HRMS (ESI) *m*/*z* found 386.1510, calcd for C_15_H_24_N_5_O_5_S^+^ [M + H]^+^ 386.1493; Elemental analysis: Calcd for C_15_H_23_N_5_O_5_S (%): C, 46.74; H, 6.01; N, 18.17; S, 8.32. Found (%): C, 46.58; H, 6.25; N, 18.01; S, 8.26.

### 4.3. Antiplatelet Activity Study

The in vitro experiments were performed using the blood of healthy male donors aged 18–24 years (total 21 donors). The study was approved by the Ethics Committee of the Bashkir State Medical University (No. 2, dated 12 November 2020). Informed consent was obtained from all participants before blood sampling. The blood was collected from the cubital vein using the system of vacuum blood collection BD Vacutainer^®^ equipment (Becton, Dickinson and Company, Franklin Lakes, NJ, USA). A 3.8% sodium citrate solution in a 9:1 ratio was used as a venous blood stabilizer. The study of the effect on platelet aggregation was performed using the Born method [47] on an «AT-02» aggregometer (SPC Medtech, Moscow, Russia). The assessment of the antiplatelet activity of the studied compounds and reference preparations was started with the final concentration of 2×10^−3^ mol/L. Adenosine diphosphate (ADP; 20 μg/mL) and collagen (5 mg/mL) manufactured by Tehnologia-Standart Company, Barnaul, Russia, were used as inducers of aggregation. The results of the study of the antiplatelet activity were processed using the statistical package Statistica 10.0 (StatSoft Inc., Tulsa, OK, USA). The Shapiro–Wilk test was used to check the normality of actual data distribution. The form of the distribution of the data obtained differed from the normal one; therefore, non-parametric methods were used for further analysis. The data were presented as medians and 25 and 75 percentiles. Analysis of variance was conducted using the Kruskal–Wallis test. A *p* value of 0.05 was considered statistically significant.

## 5. Conclusions

New 8-amino-substituted 1-isobutyl-3-methylpurine-2,6-diones containing thietane, thietane oxide, and thietane dioxide rings were synthesized from the key 8-bromo-1-isobutyl-7-(thietan-3-yl)purine-2,6-dione via oxidation of the thietane ring and subsequent reaction with amines. Analysis of two-dimensional correlation NMR spectra allowed assignment of the signals of the methylene protons of the thietane ring to the axial and equatorial arrangements. The results of biological activity screening showed that the synthesized compounds **6d**,**g**,**h** exhibit activity similar to that of acetylsalicylic acid, while compounds **6b**,**c**,**f** exceed the activity of acetylsalicylic acid. Our future research in this area will focus on investigating the mechanism of action and designing target molecules with improved antiplatelet aggregation properties to evaluate the potential of 8-substituted 1-alkyl-3-methylpurine-2,6-diones containing a thietane ring.

## Figures and Tables

**Table 1:** Measured ^1^ and calculated *J_HH_* ^2^, *J_CH_* ^3^ and *J_NH_* ^3^ for compound **2** in Hz.

*J*	Exp.	Calcd.	Valence and Dihedral Angles	
			M06-2X/6-311+G(d,p)	B3LYP/6-311++G(d,p)
^2^ *J_H_*_2*a*-*H*2*e*_	8.6	–7.9	111.7°	112.0°
^3^ *J_H_*_3-*H*2*a*_	9.7	9.1	160.3°	159.4°
^3^ *J_H_*_3-*H*2*e*_	8.6	8.1	29.1°	28.3°
^3^ *J_C_*_4-*H*2*e*_	~9	9.1	138.6°	141.4°
^3^ *J_C_*_4-*H*2*a*_	~0	0.8	94.4°	87.4°
^3^ *J_N_*_7-*H*2*e*_	~0	0.1	91.1°	92.9°
^3^ *J_N_*_7-*H*2*a*_	~1	1.3	40.1°	38.2°

^1^ DMSO-*d*_6_, 500 MHz, with experimental values (*J*) given as magnitudes; ^2^ M06-2X/6-311+G(d,p)//mPW1PW91/6-311+G(2d,p) GIAO PCM (DMSO); ^3^ M06-2X/6-311+G(d,p)//B3LYP/6-31G(d,p) u+1s.

**Table 2:** Antiplatelet activity of test compounds.

Cmpd	Latent Period, % of Control	Maximum Amplitude (MA), % of Control	Aggregation Rate, % of Control
**6a**	+3.7 (3.5–5.2) *	−3.0 (2.1–4.8) #	−7.6 (7.1–8.6) *,#
**6b**	+12.7 (10.9–14.3) *,#	−19.6 (17.3–20.5) *,#	−27.3 (23.1–30.2) *,#
**6c**	+11.3 (10.5–14.1) *,#	−19.3 (16.2–22.5) *,#	−26.8 (22.3–27.5) *,#
**6d**	+12.9 (10.4–15.6) *,#	−15.9 (13.5–17.9) *	−9.7 (7.8–10.4) *
**6e**	+9.2 (9.1–11.4) *,#	+8.4 (7.6–9.3) *,#	+9.1 (7.6–11.2) *,#
**6f**	+11.2 (9.3–12.4) *,#	−16.2 (14.7–19.2) *	−28.5 (24.7–30.4) *,#
**6g**	+2.4 (1.9–3.5)	−17.5 (15.3–19.8) *	+2.4 (1.7–3.4) *,#
**6h**	−4.2 (3.9–5.7)	−15.5 (14.3–17.2) *	−21.9 (20.5–23.5) *,#
**6i**	+1.2 (0.7–2.1)	−8.1 (7.3–9.5) *,#	−6.9 (6.3–8.1) *,#
**6j**	+4.3 (3.2–5.7) #	−2.5 (1.4–6.7) #	+1.3 (0.9–1.7) #
**6k**	−3.3 (2.7–4.1)	−7.9 (6.5–9.6) *,#	−4.3 (3.2–5.7) #
**6m**	+3.2 (2.7–4.3)	−7.1 (6.7–9.4) *,#	−26.9 (24.8–30.1) *,#
**Acetylsalicylic acid**	−2.1 (1.1–2.6)	−13.7 (10.8–16.4) *	−10.5 (7.6–12.3) *

The latent period is presented for collagen-induced platelet aggregation, the maximum amplitude and aggregation rate—for ADP-induced platelet aggregation. * *p* < 0.05—compared to control; # *p* < 0.05—compared to acetylsalicylic acid.

## Data Availability

Data are available in the manuscript.

## Supplementary Materials

The following supporting information can be downloaded at: https://www.mdpi.com/article/10.3390/molecules31193521/s1, Figures S1–S18: copies of NMR spectra of all synthesized compounds, Figures S19–S36: copies of HRMS of all synthesized compounds, Table S1: HRMS data for all synthesized compounds, Figures S37–S54: Copies of IR spectra of all synthesized compounds.
