# Supplementary material for: Stereochemical 2D NMR Assignment of Thietane, Synthesis and Antiplatelet Activity of Novel Thietane-Containing 1-Isobutylpurine-2,6-dione Derivatives as Potential GPIIb/IIIa Inhibitors
Source: Molecules. 2026 Oct 2;31(19):3521. doi: 10.3390/molecules31193521 (PMC13666470; doi:10.3390/molecules31193521)
Supplement: Supplementary file 1 [file molecules-31-03521-s001.zip › molecules-4549095-supplementary.pdf]

Supporting Information

For

Stereochemical 2D NMR Assignment of Thietane, Synthesis and Antiplatelet Activity of Novel Thietane-Containing 1-Isobutylpurine-2,6-dione Derivatives as Potential GPIIb/IIIa Inhibitors

NMR spectra

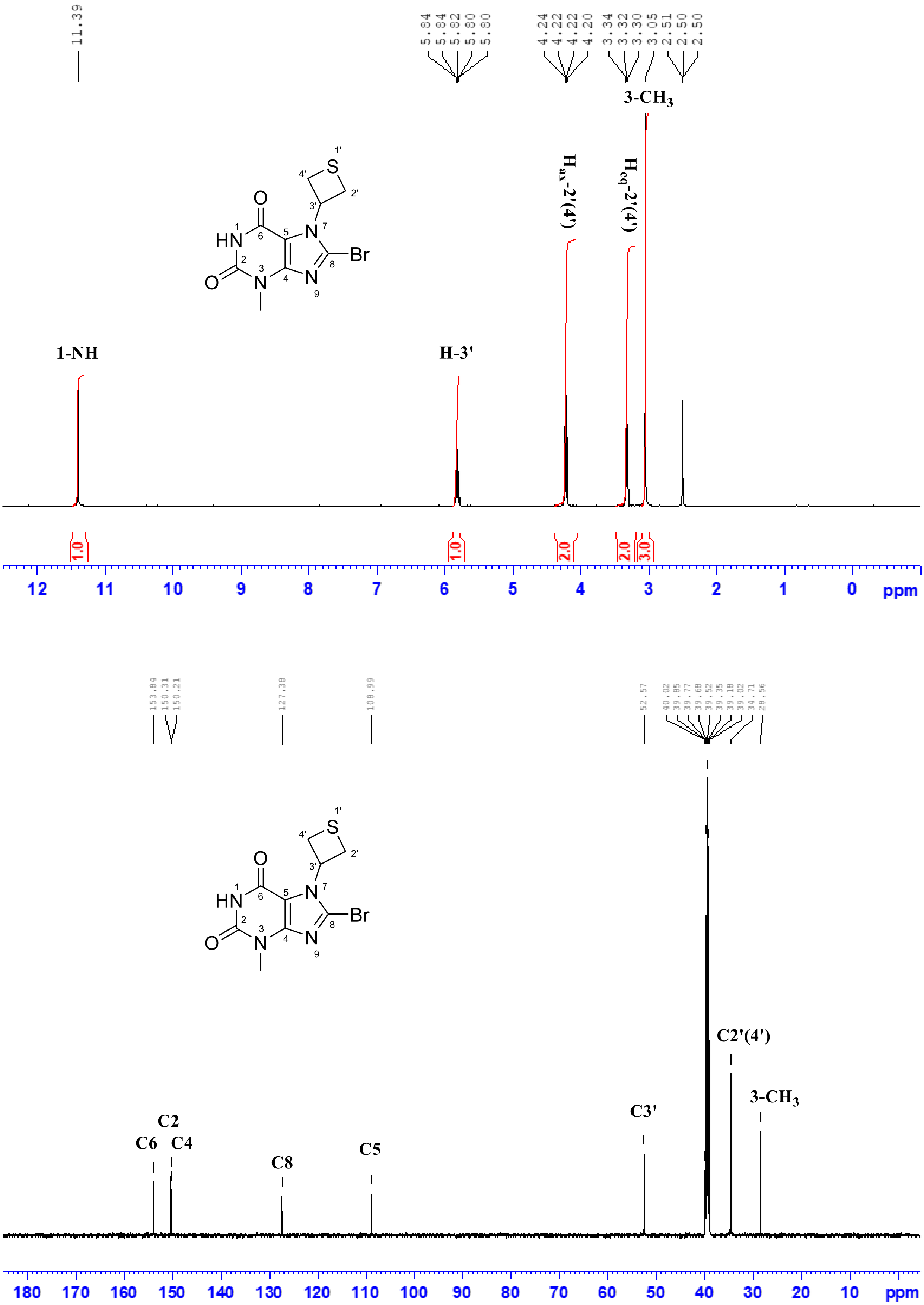

Figure S1.1: ^{1}H NMR (500 MHz, DMSO-d_6) and ^{13}C NMR (125 MHz, DMSO-d_6) spectra of **2**

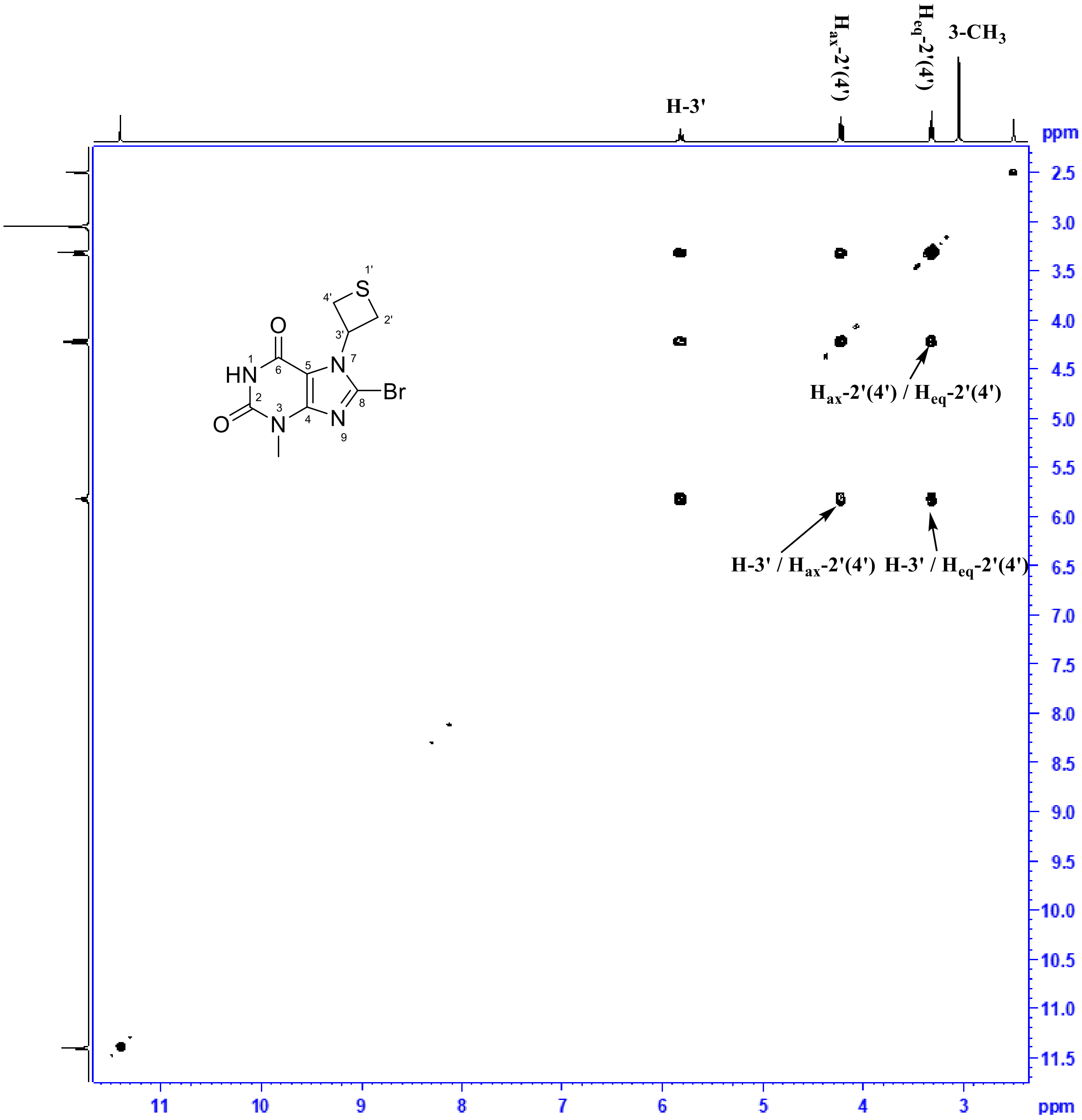

Figure S1.2: {^{1}H,^{1}H} COSY spectra of **2**

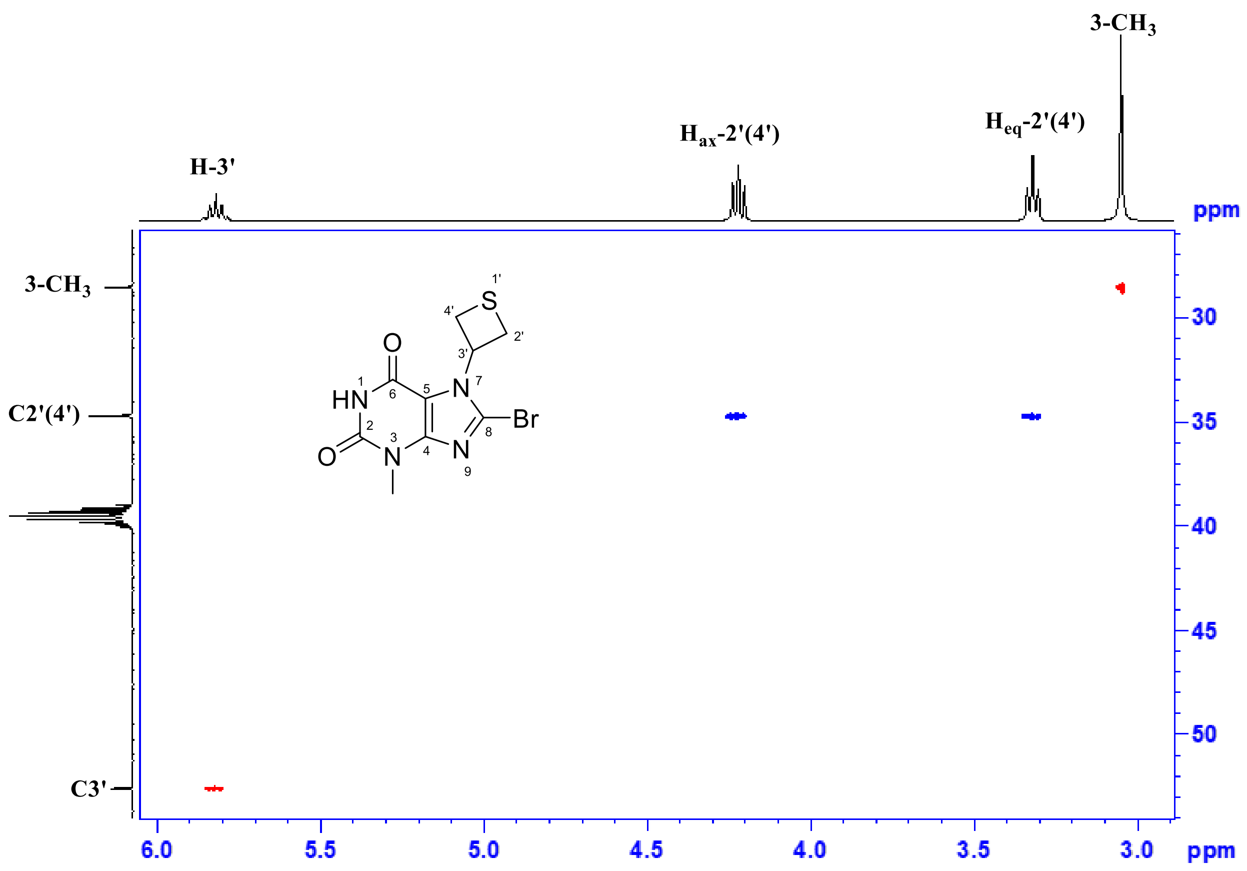

Figure S1.3: {^{1}H,^{13}C} HSQC spectra of **2**

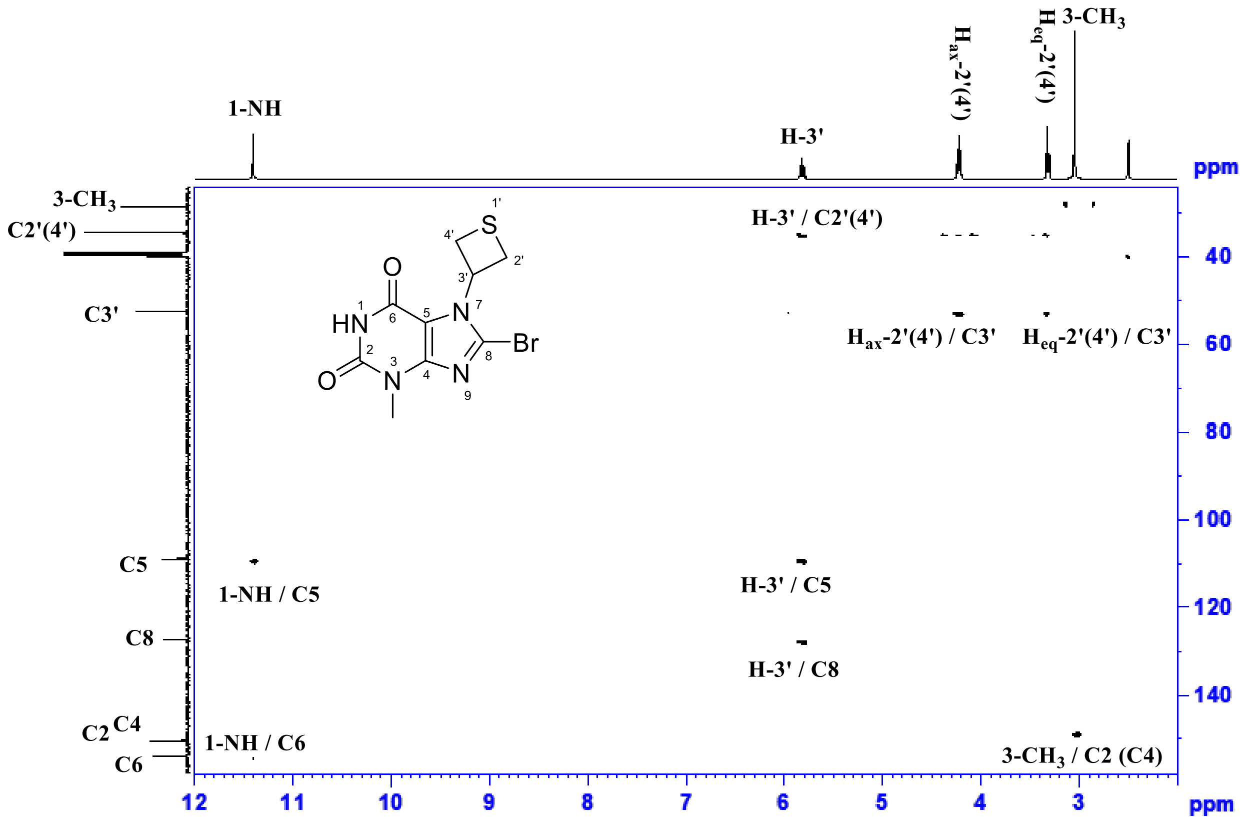

Figure S1.4: {^{1}H,^{13}C} HMBC spectra of **2**

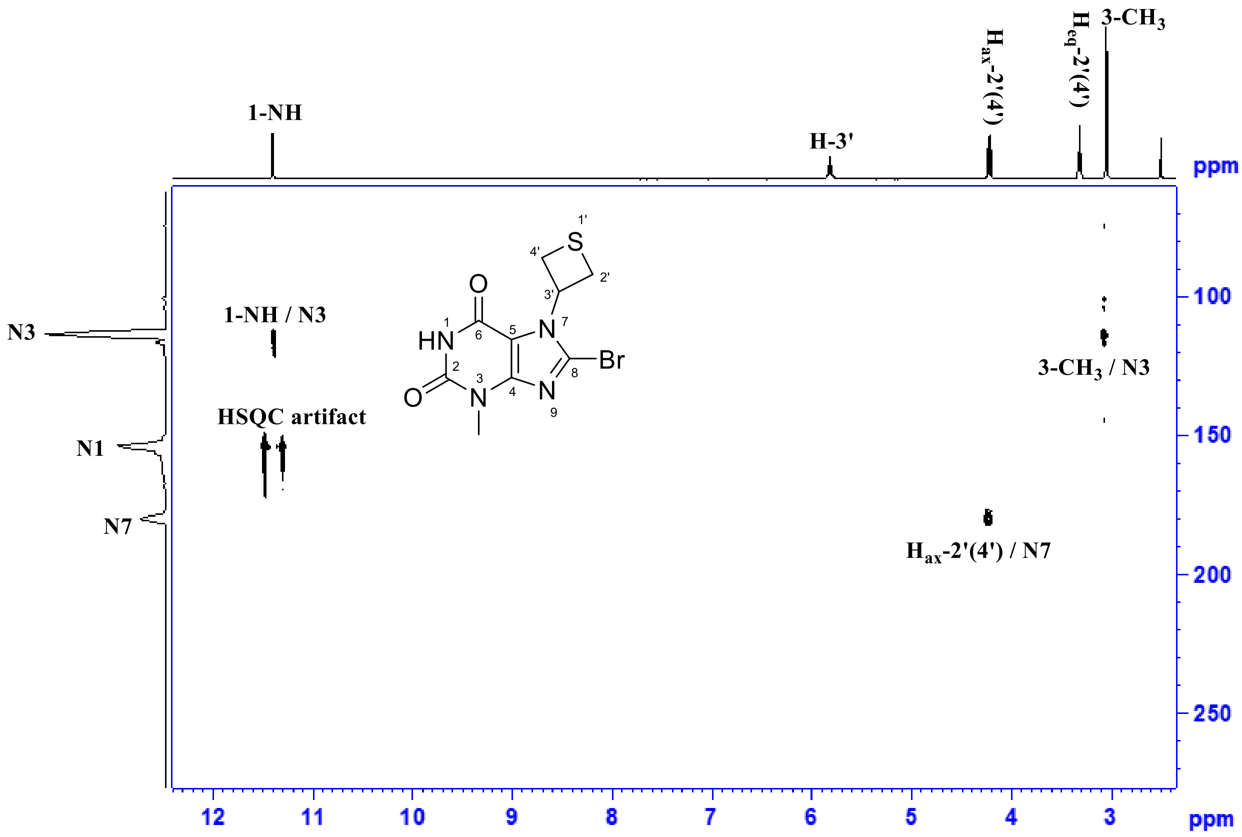

Figure S1.5: {^{1}H,^{15}N} HMBC spectra of **2**

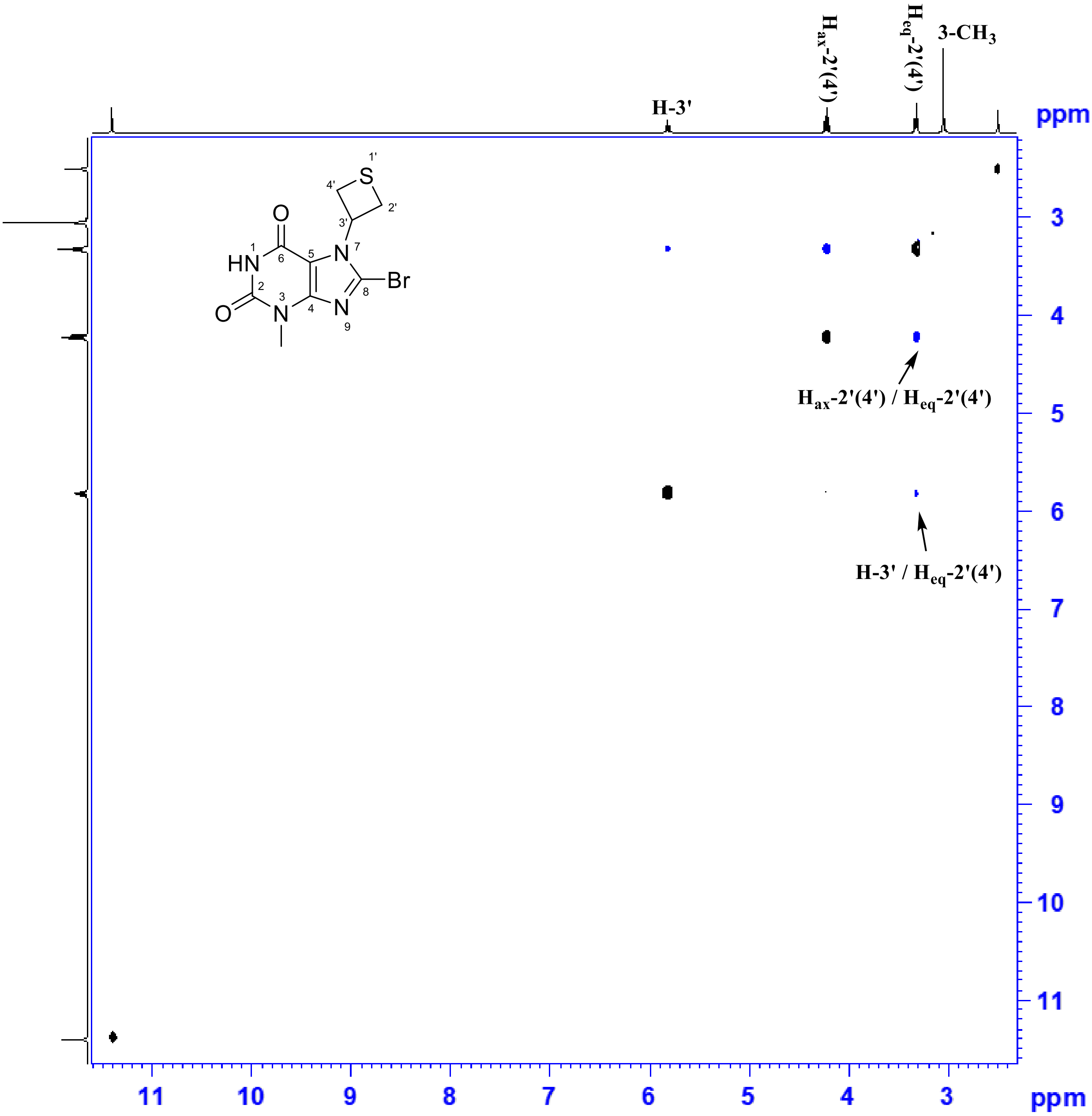

Figure S1.6: {^{1}H,^{1}H} NOESY spectra of **2**

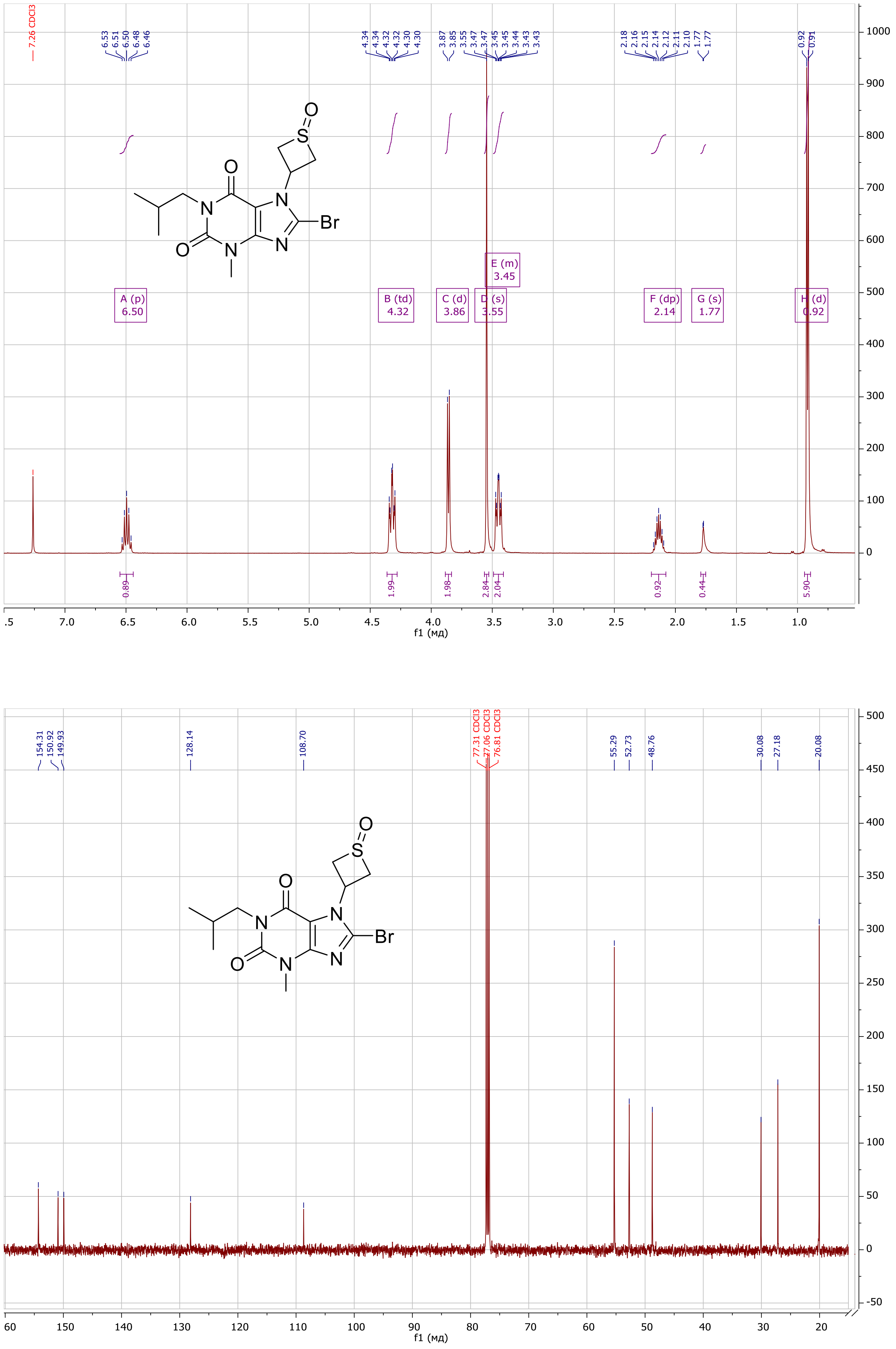

Figure S2: ^{1}H NMR (500 MHz, $CDCl_3$) and ^{13}C NMR (125 MHz, $CDCl_3$) spectra of **4**

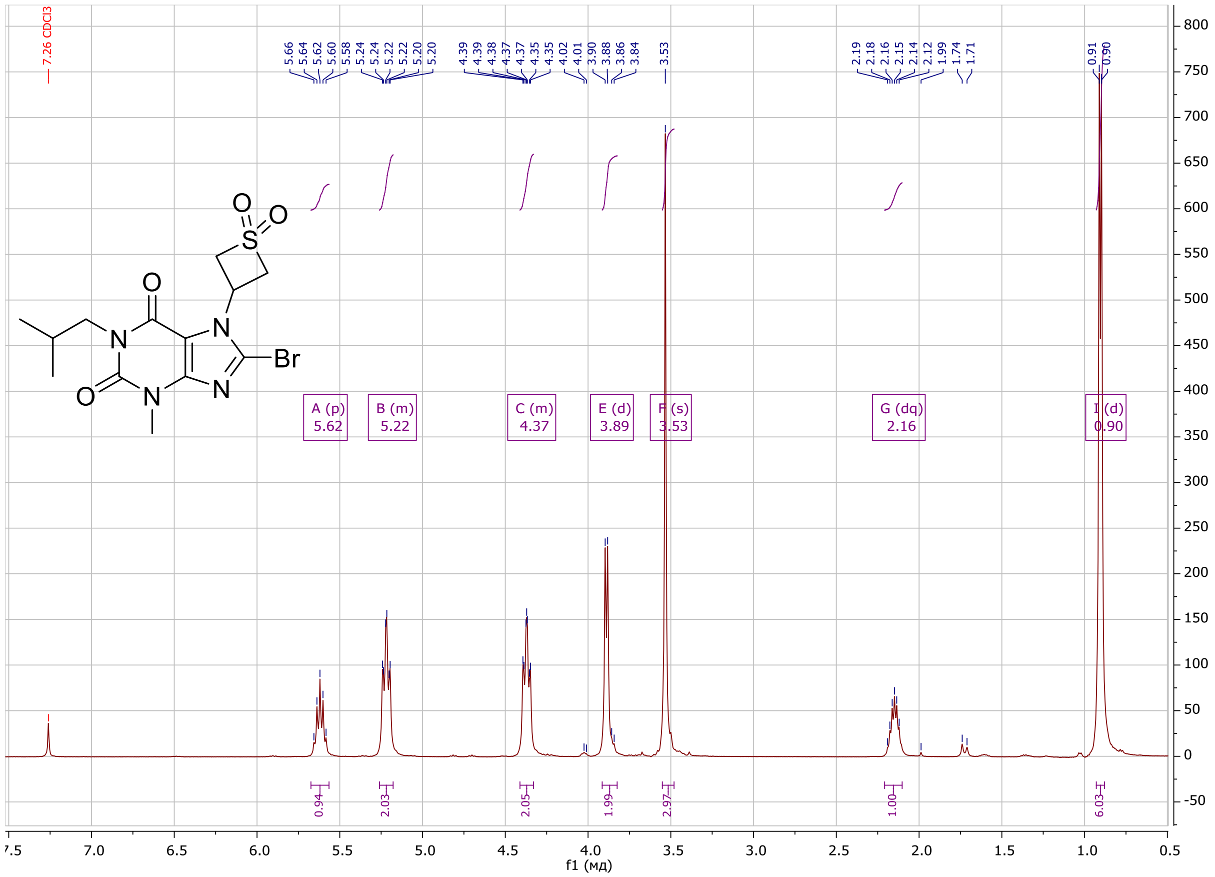

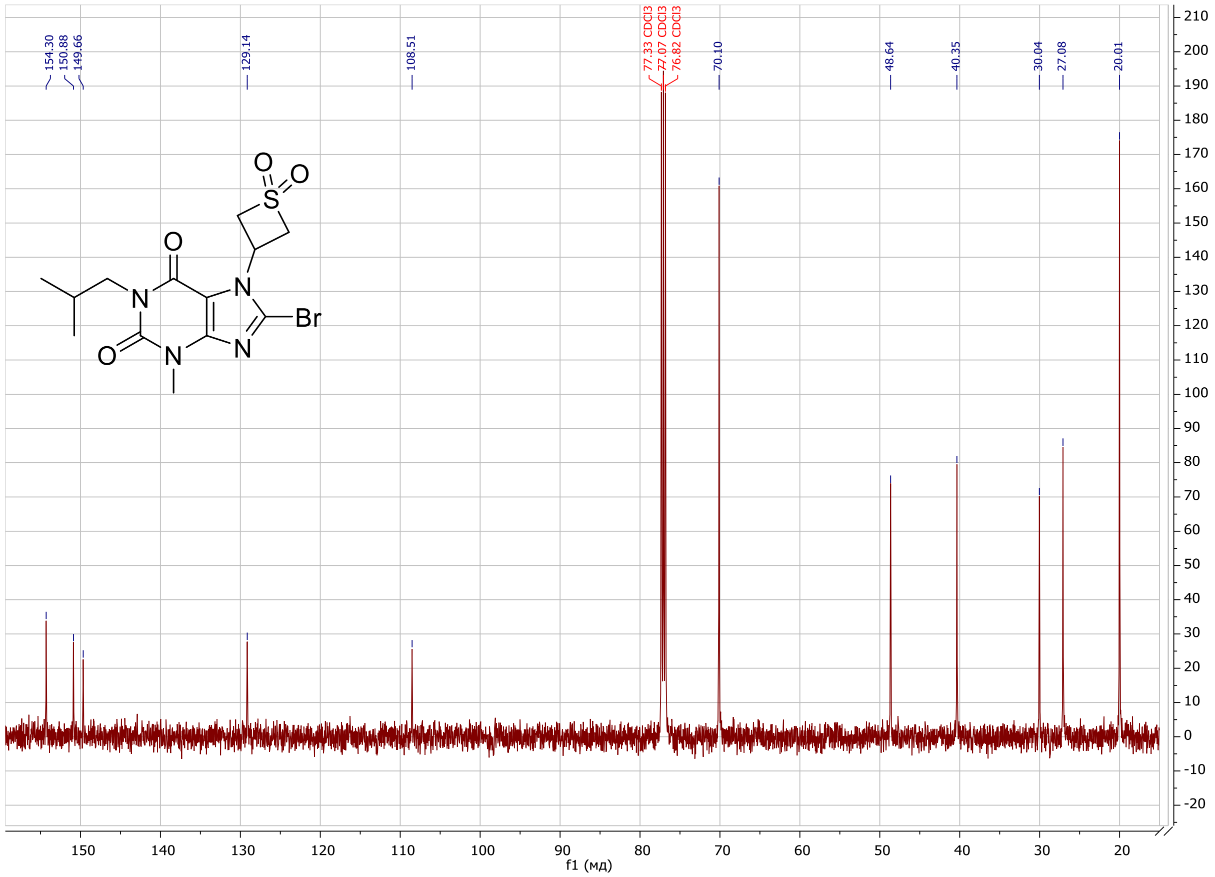

Figure S3: ^{1}H NMR (500 MHz, $CDCl_3$) and ^{13}C NMR (125 MHz, $CDCl_3$) spectra of **5**

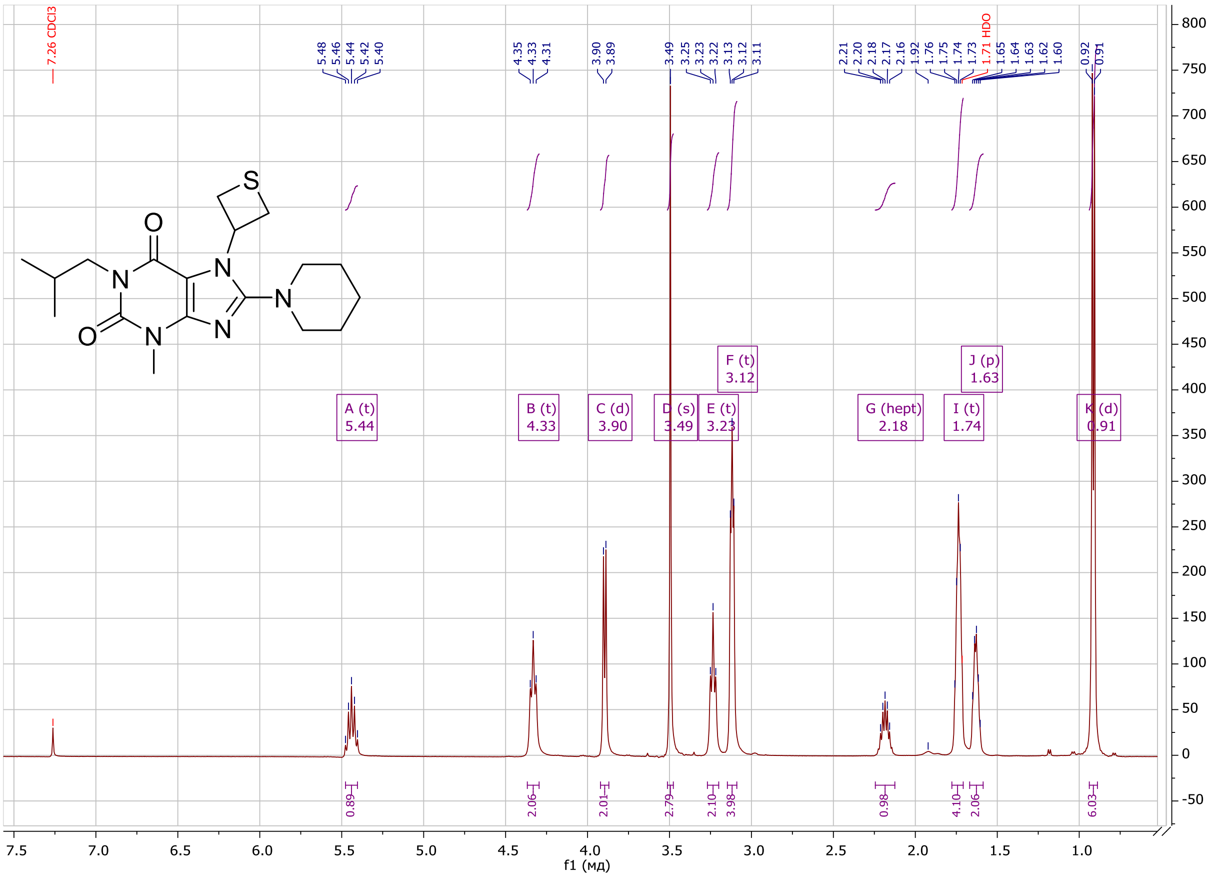

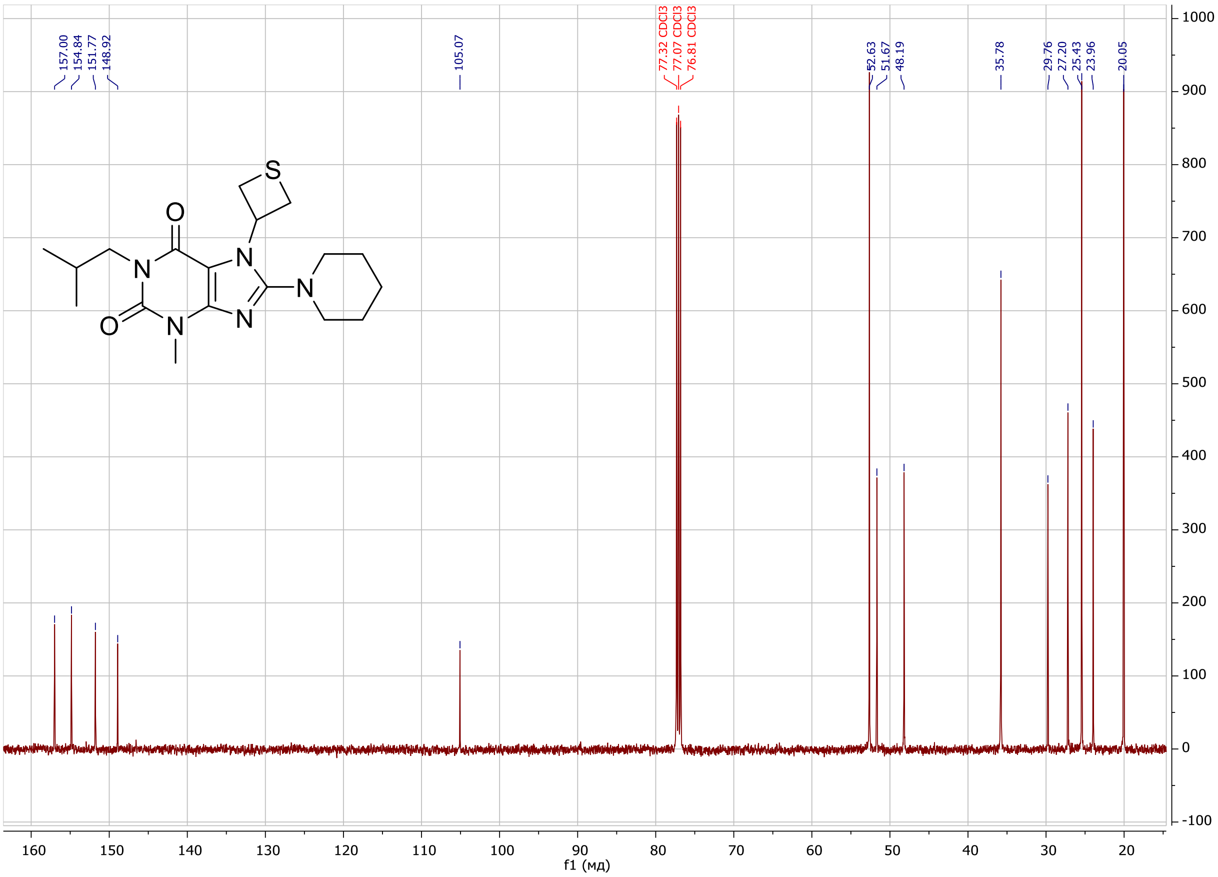

Figure S4: ^{1}H NMR (500 MHz, $CDCl_3$) and ^{13}C NMR (125 MHz, $CDCl_3$) spectra of **6a**

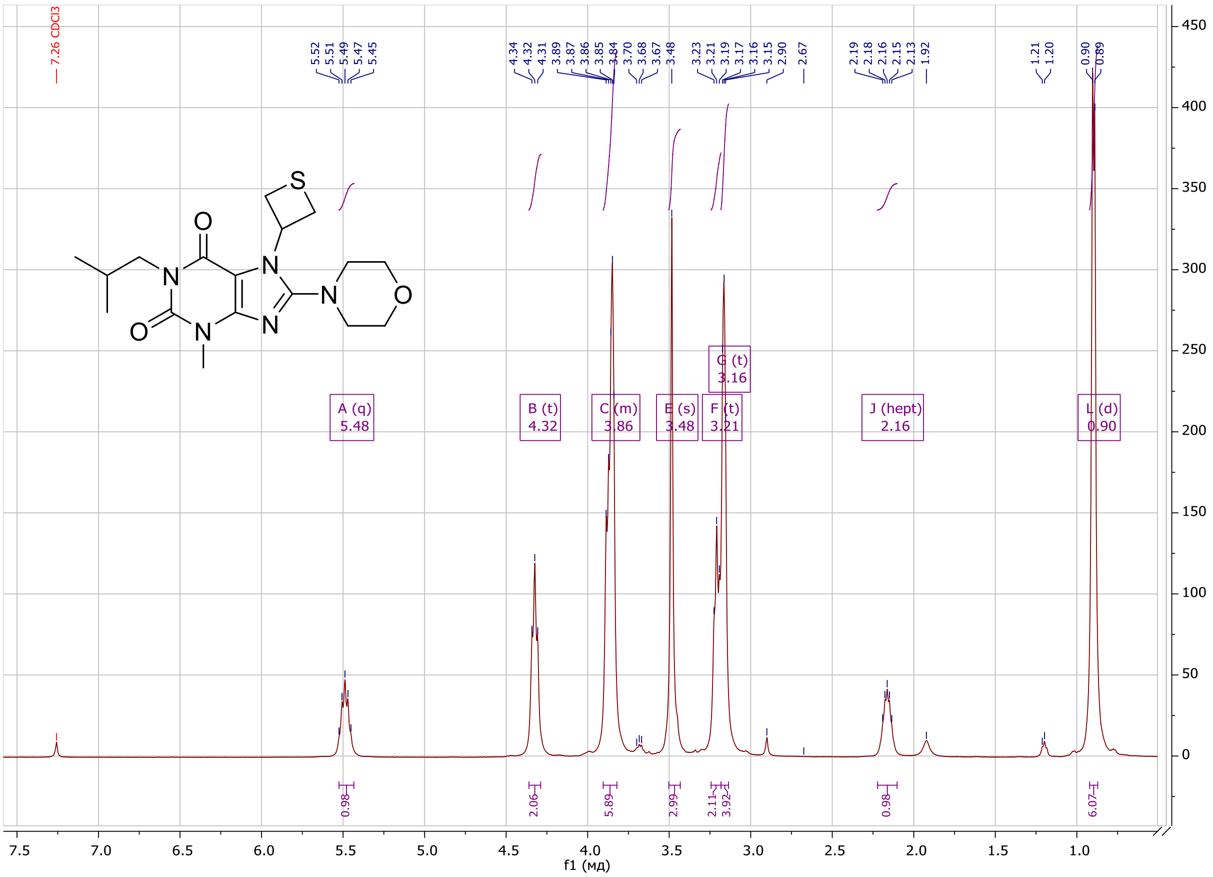

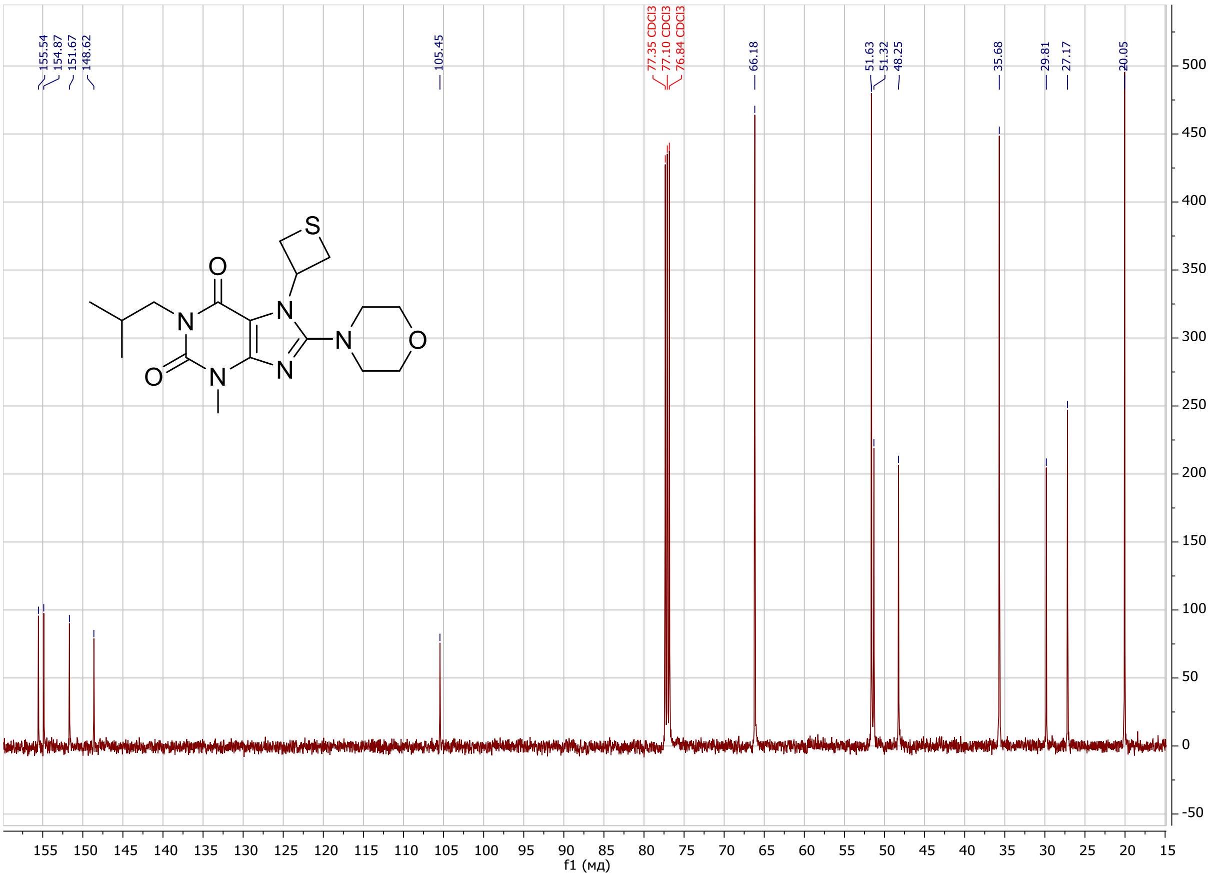

Figure S5: ^{1}H NMR (500 MHz, $CDCl_3$) and ^{13}C NMR (125 MHz, $CDCl_3$) spectra of **6b**

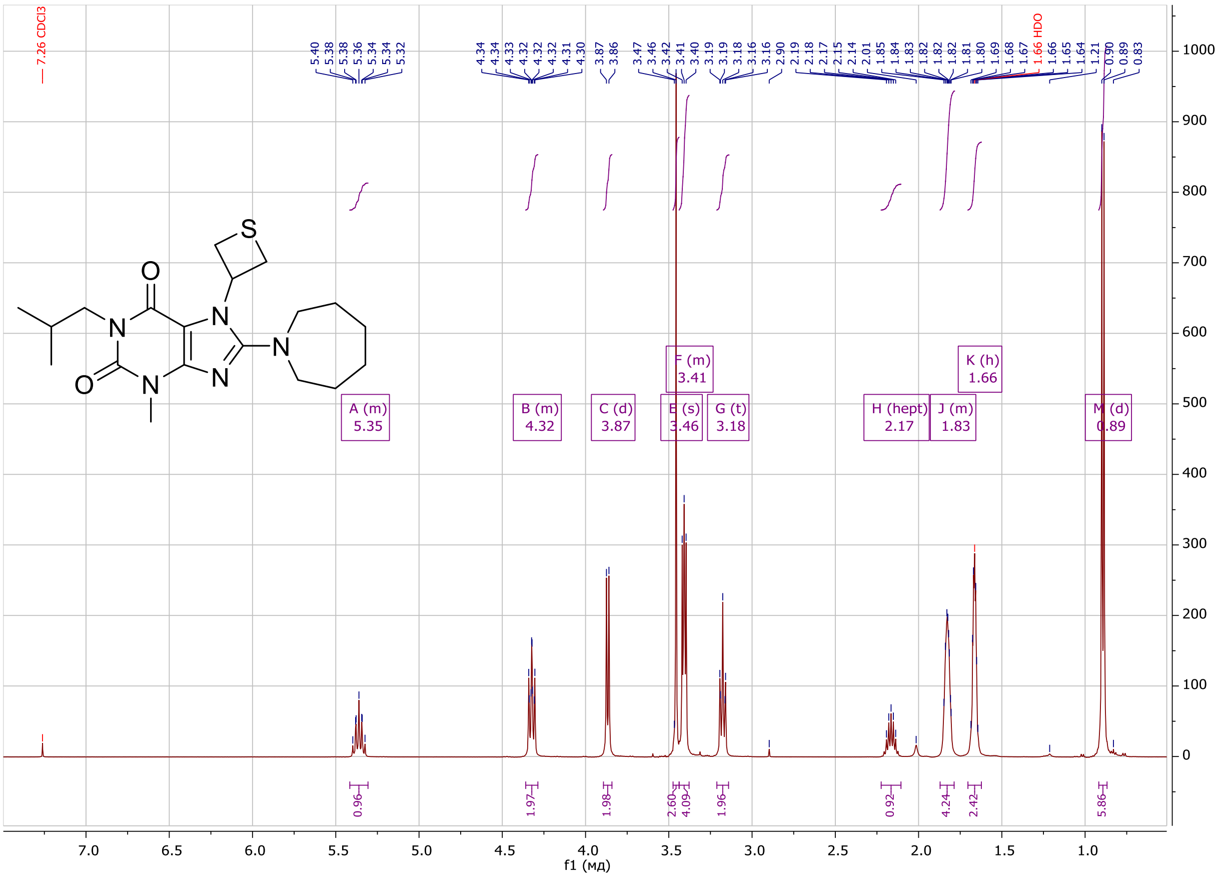

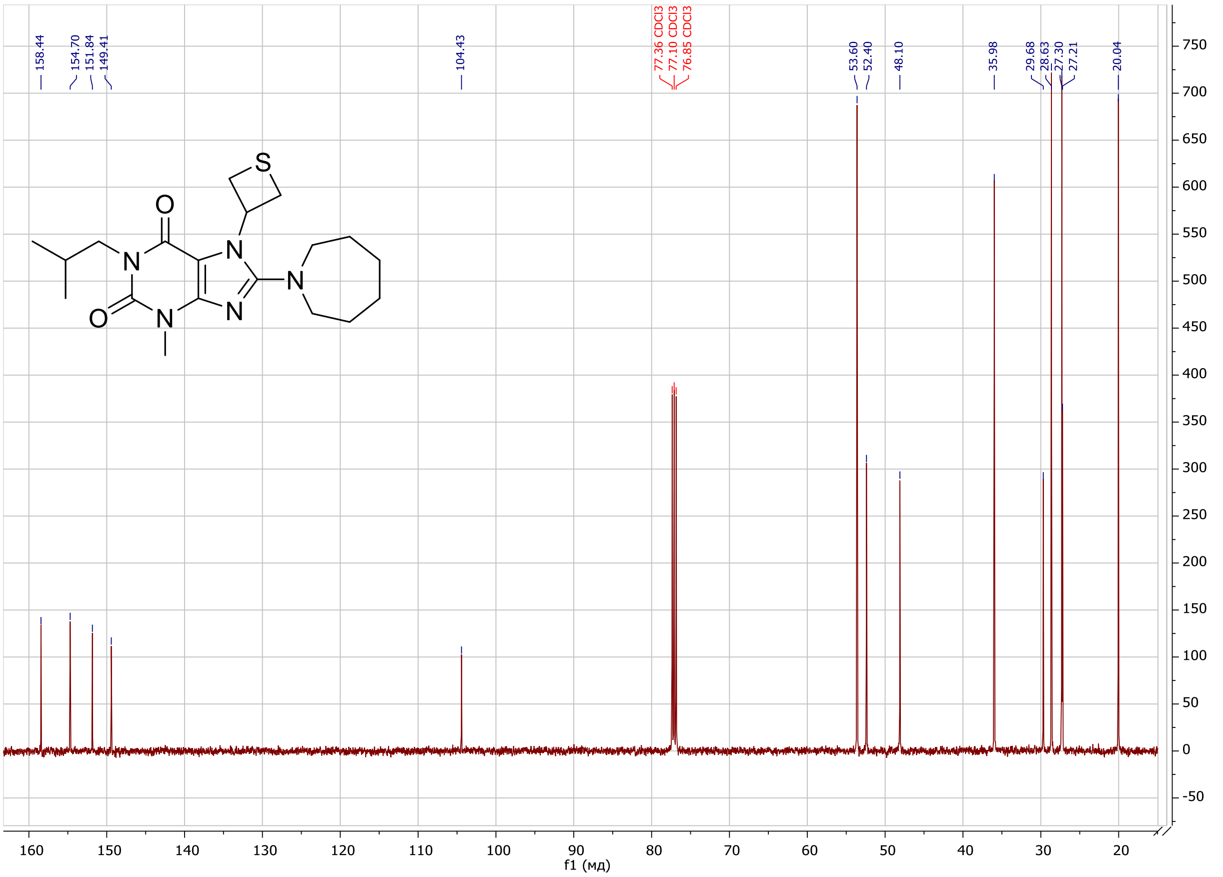

Figure S6: ^{1}H NMR (500 MHz, $CDCl_3$) and ^{13}C NMR (125 MHz, $CDCl_3$) spectra of **6c**

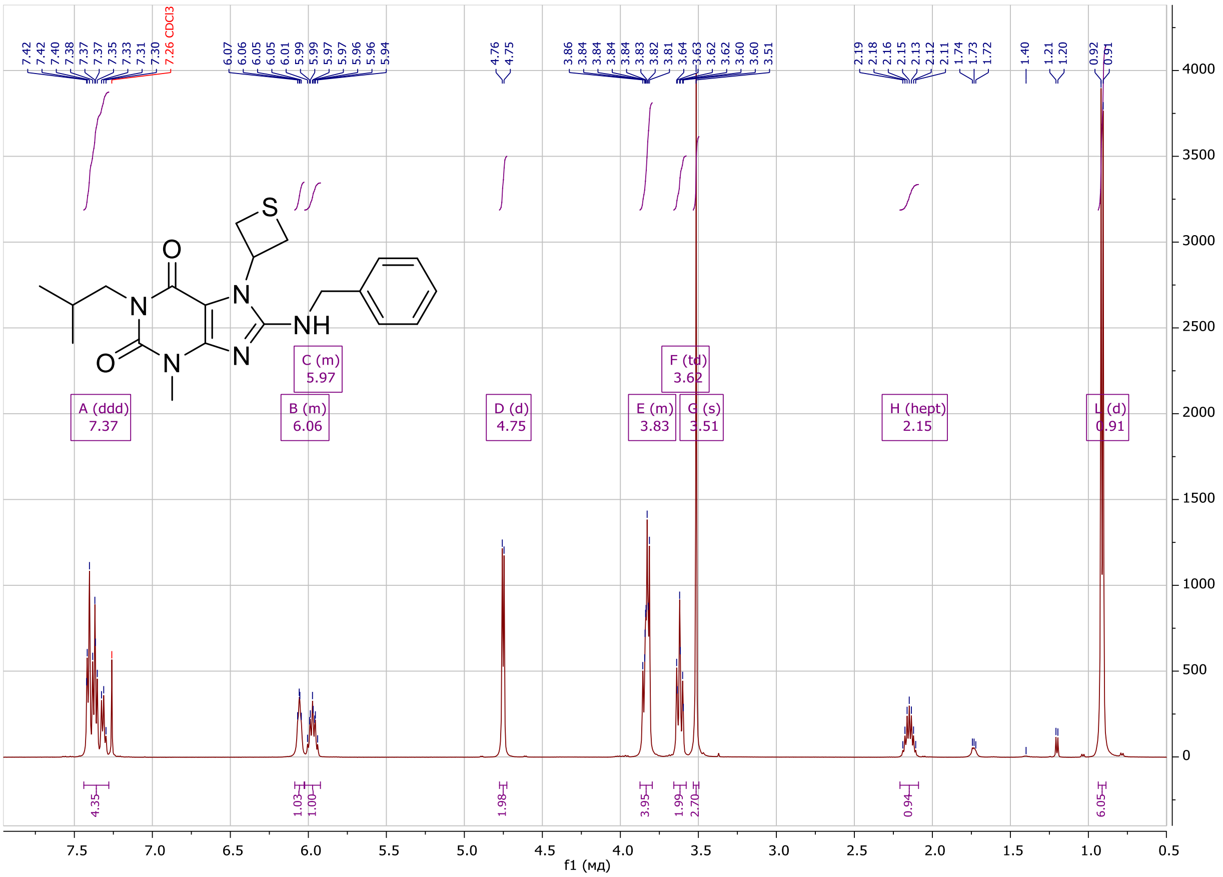

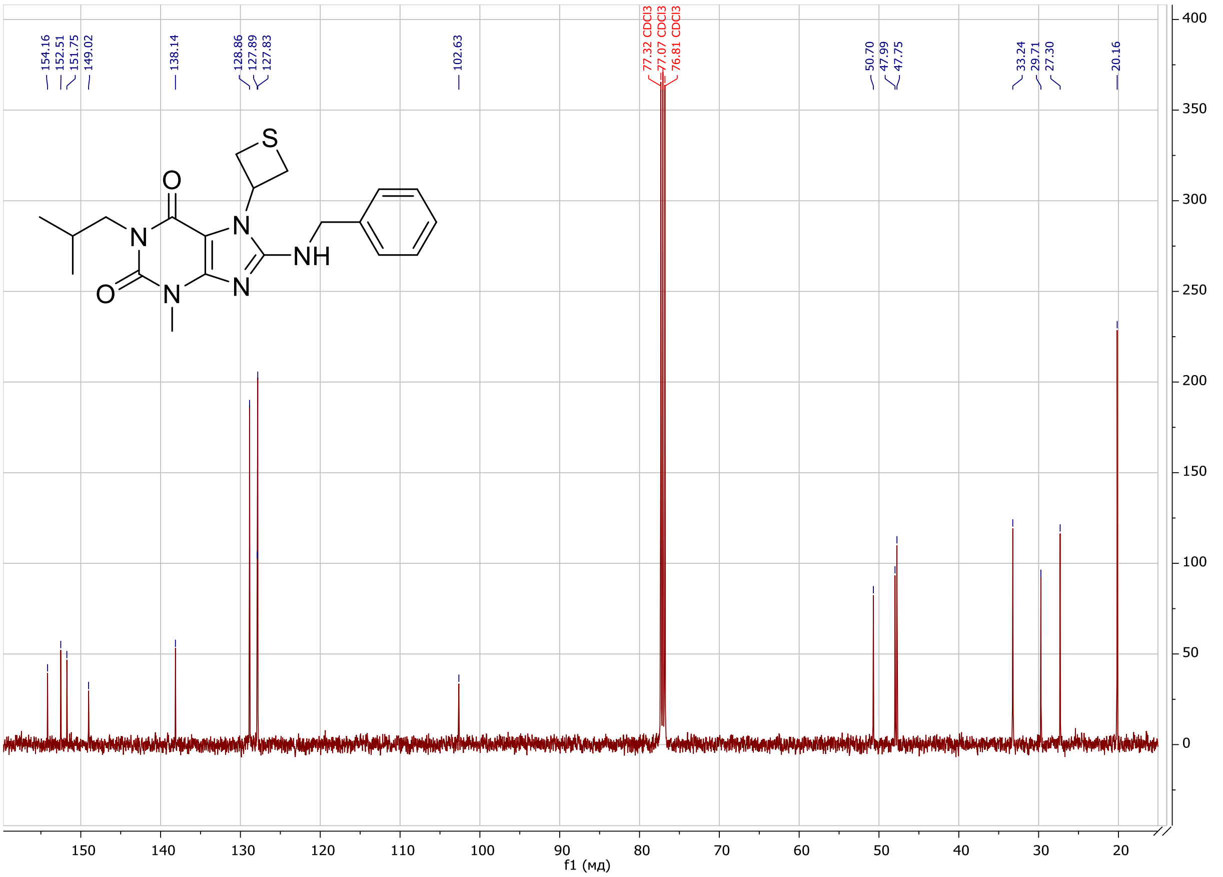

Figure S7: ^{1}H NMR (500 MHz, $CDCl_3$) and ^{13}C NMR (125 MHz, $CDCl_3$) spectra of **6d**

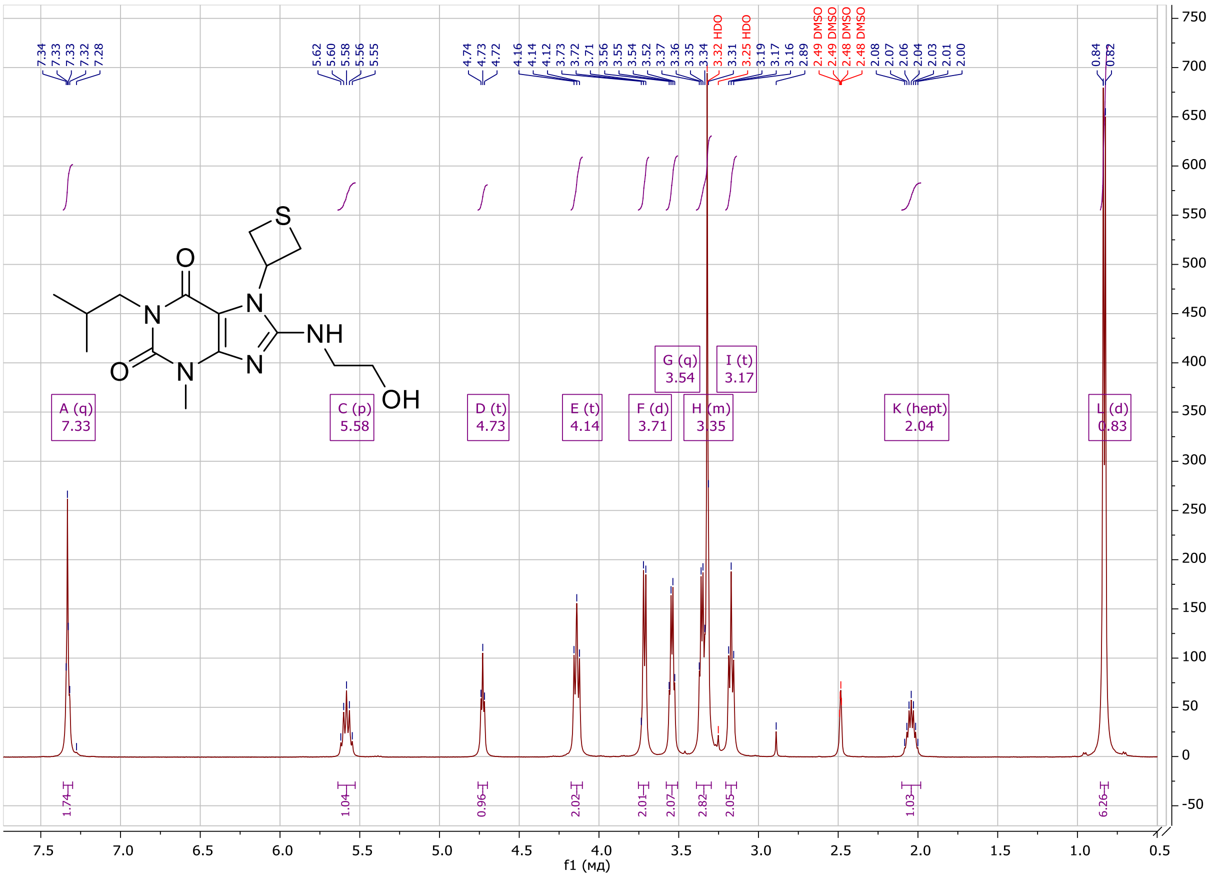

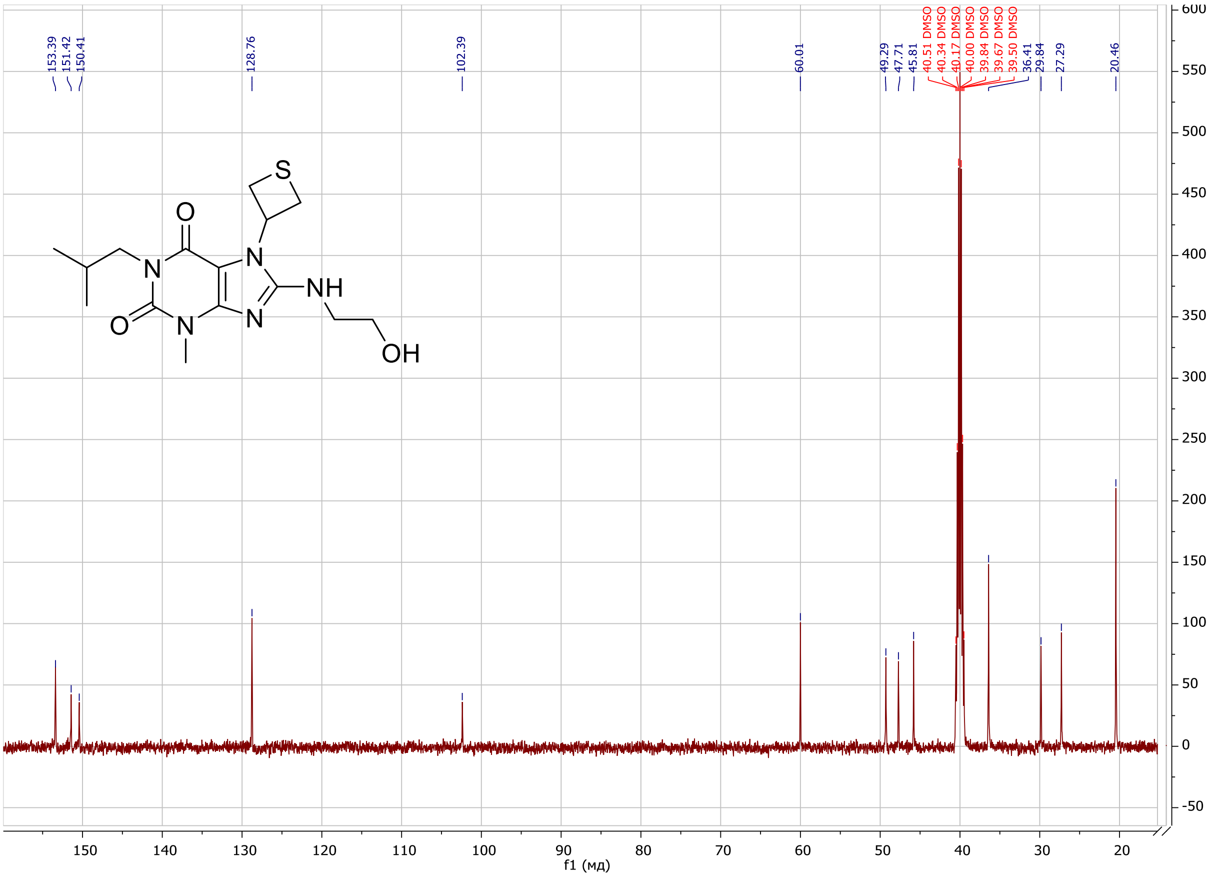

Figure S8: ^{1}H NMR (500 MHz, DMSO-d_6) and ^{13}C NMR (125 MHz, DMSO-d_6) spectra of **6e**

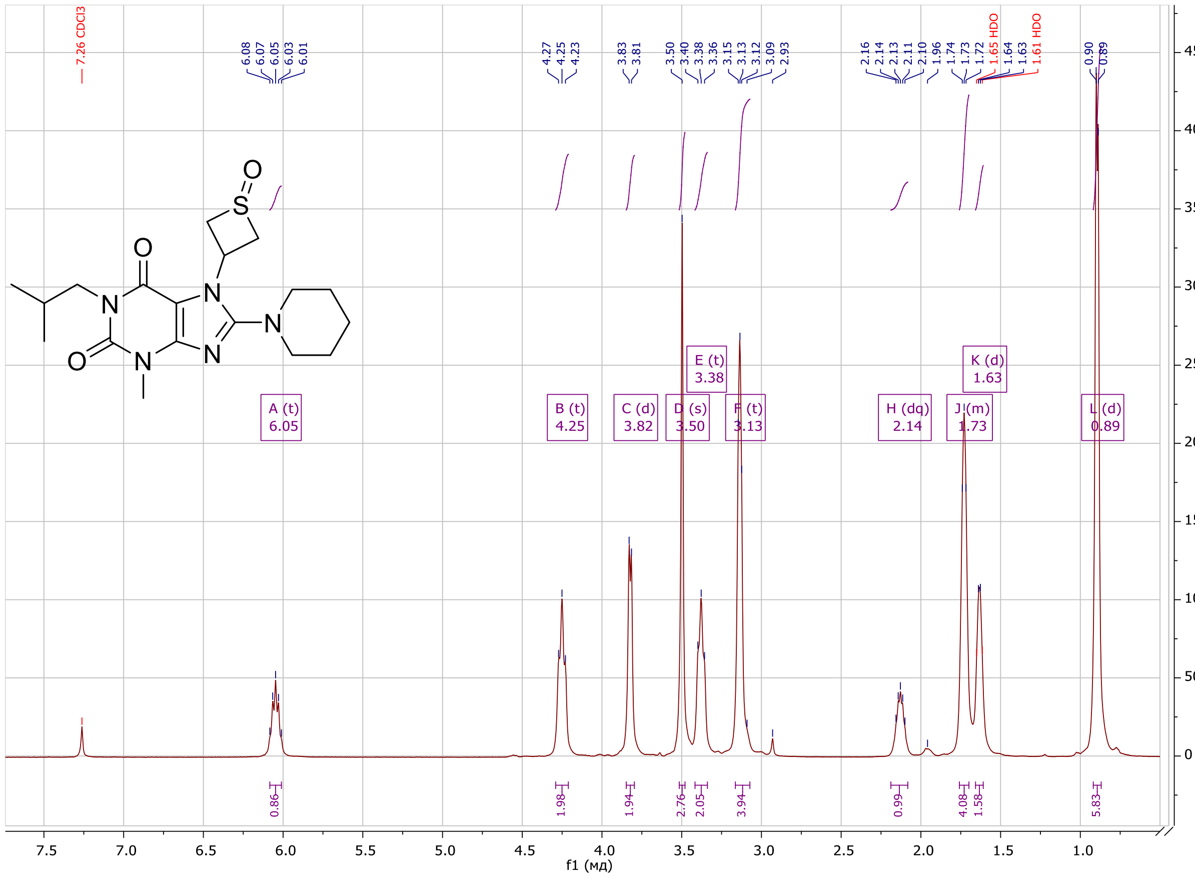

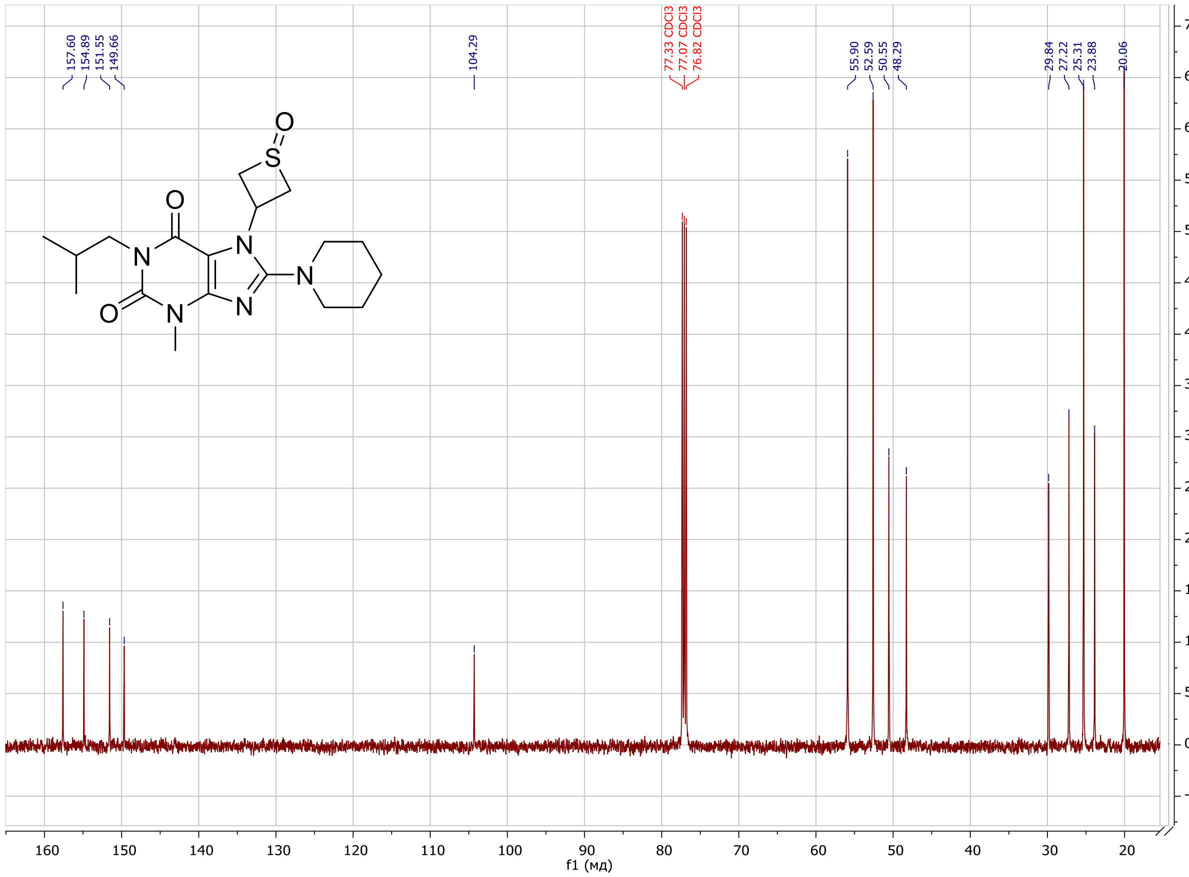

Figure S9: ^{1}H NMR (500 MHz, $CDCl_3$) and ^{13}C NMR (125 MHz, $CDCl_3$) spectra of **6f**

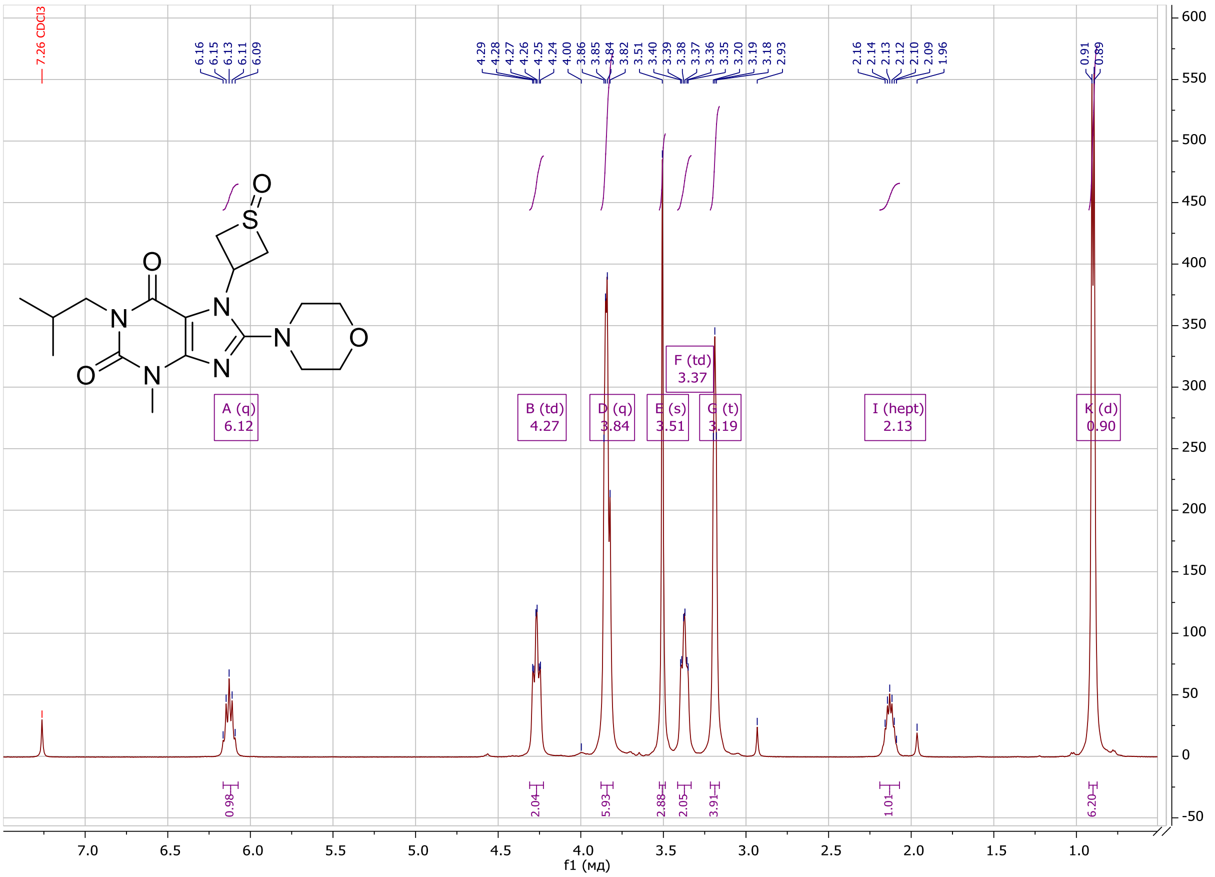

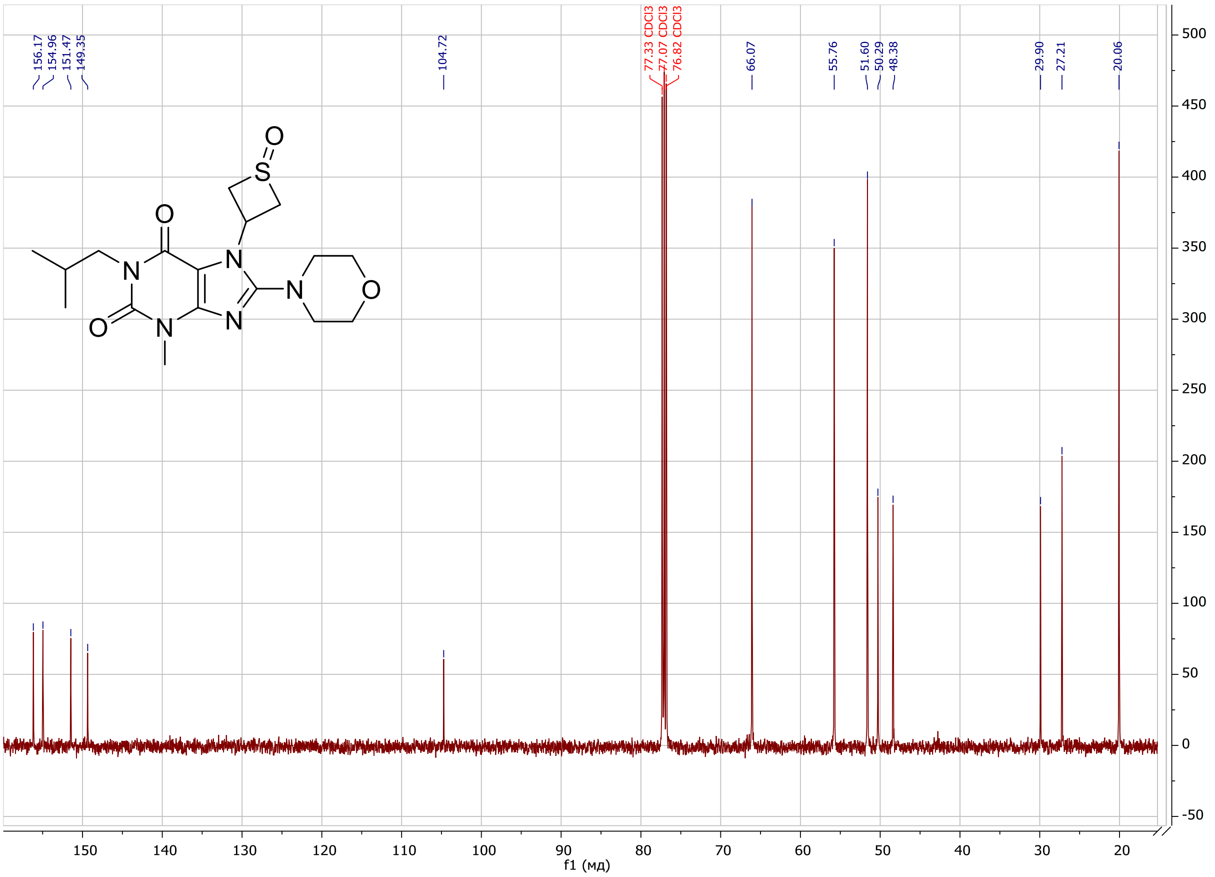

Figure S10: ^{1}H NMR (500 MHz, $CDCl_3$) and ^{13}C NMR (125 MHz, $CDCl_3$) spectra of **6g**

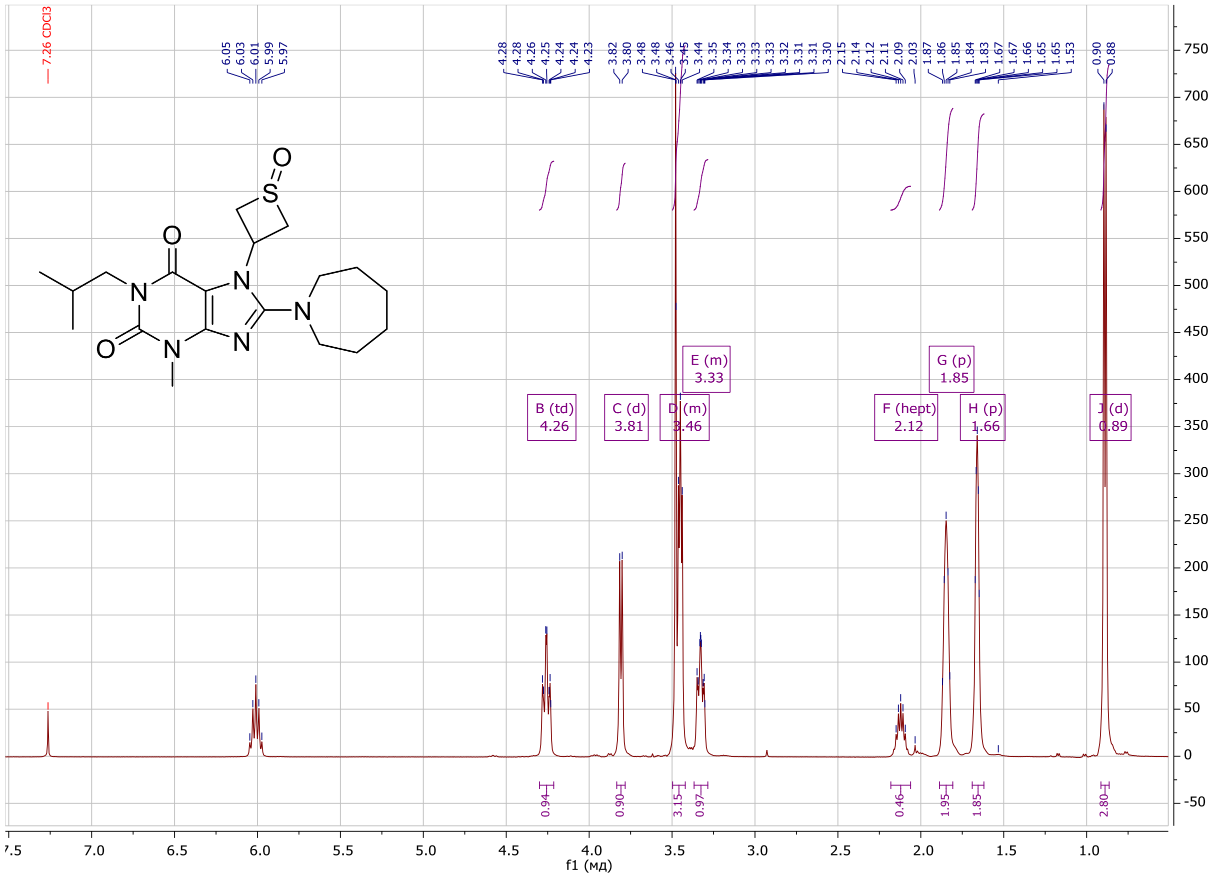

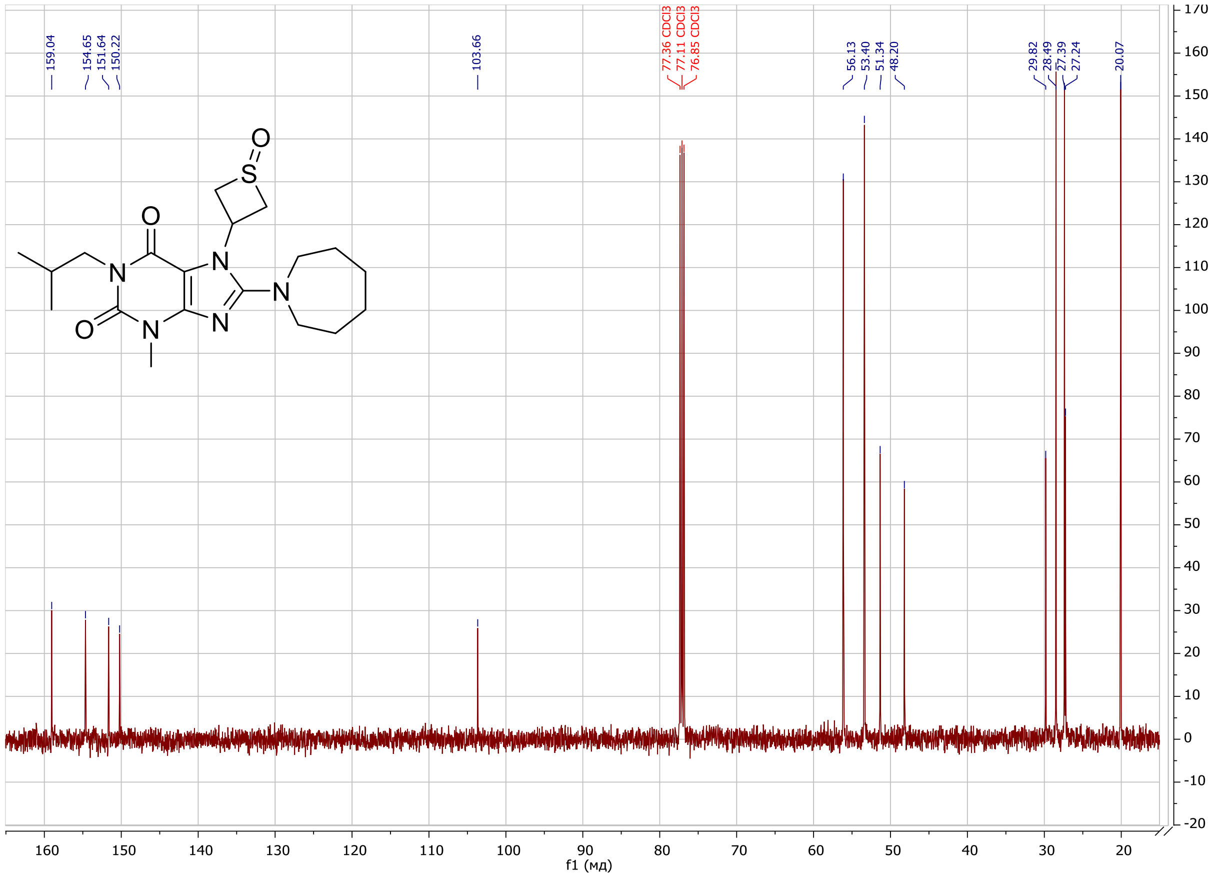

Figure S11: ^{1}H NMR (500 MHz, $CDCl_3$) and ^{13}C NMR (125 MHz, $CDCl_3$) spectra of **6h**

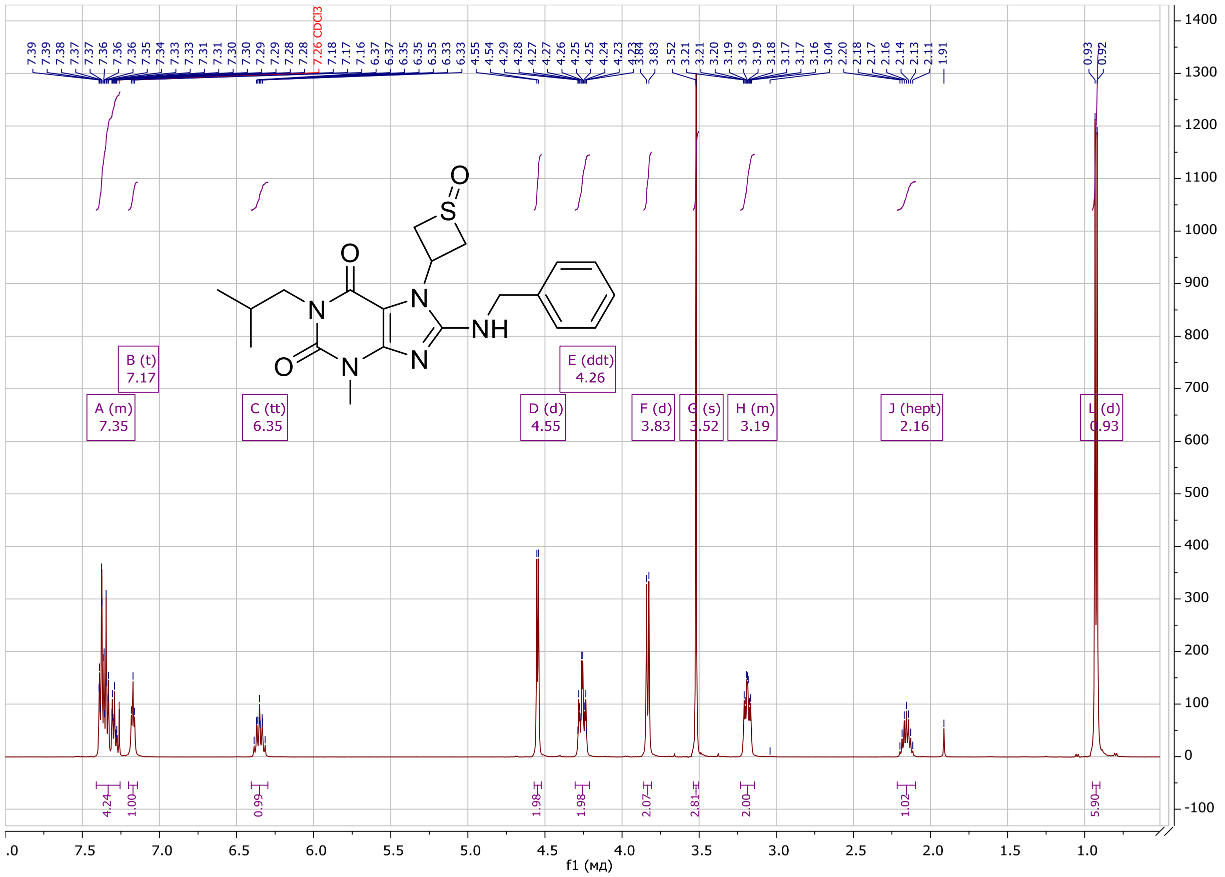

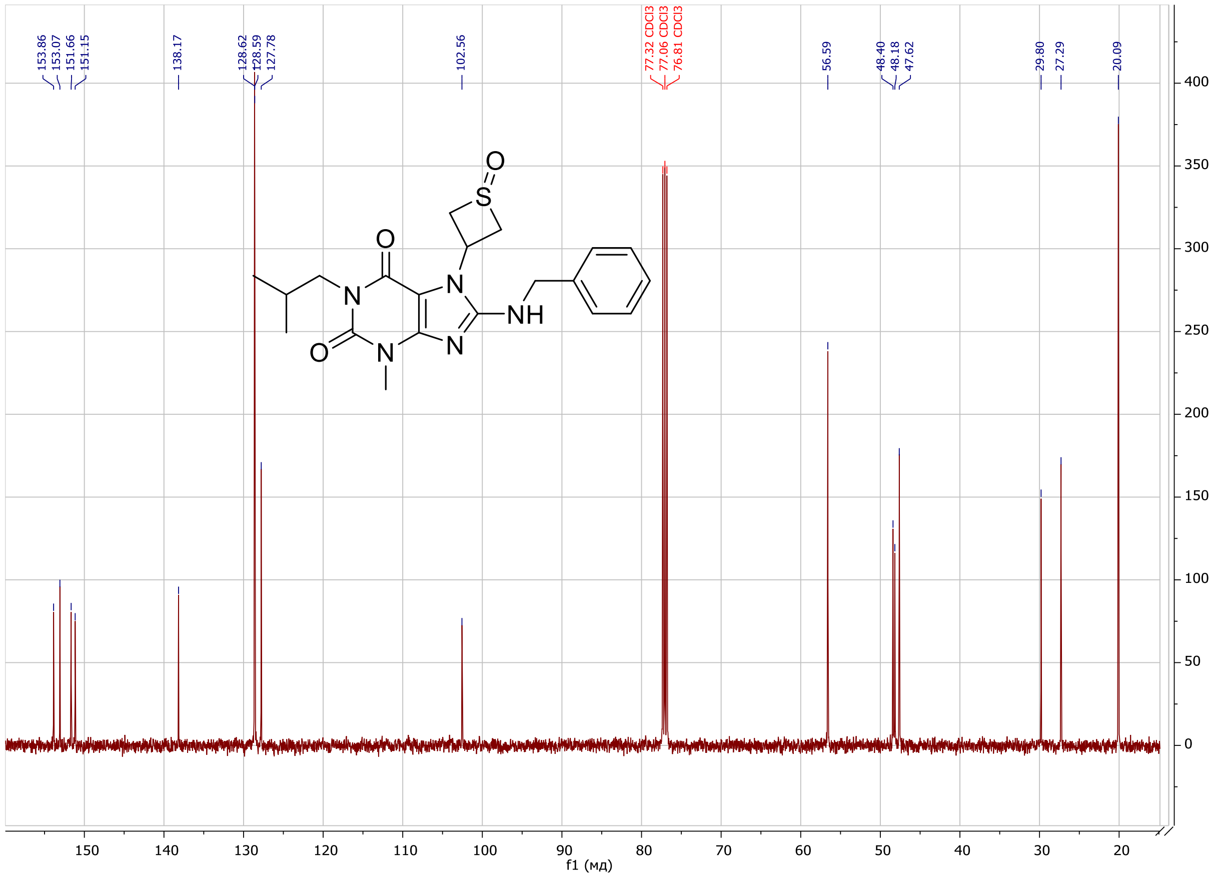

Figure S12: ^{1}H NMR (500 MHz, $CDCl_3$) and ^{13}C NMR (125 MHz, $CDCl_3$) spectra of **6i**

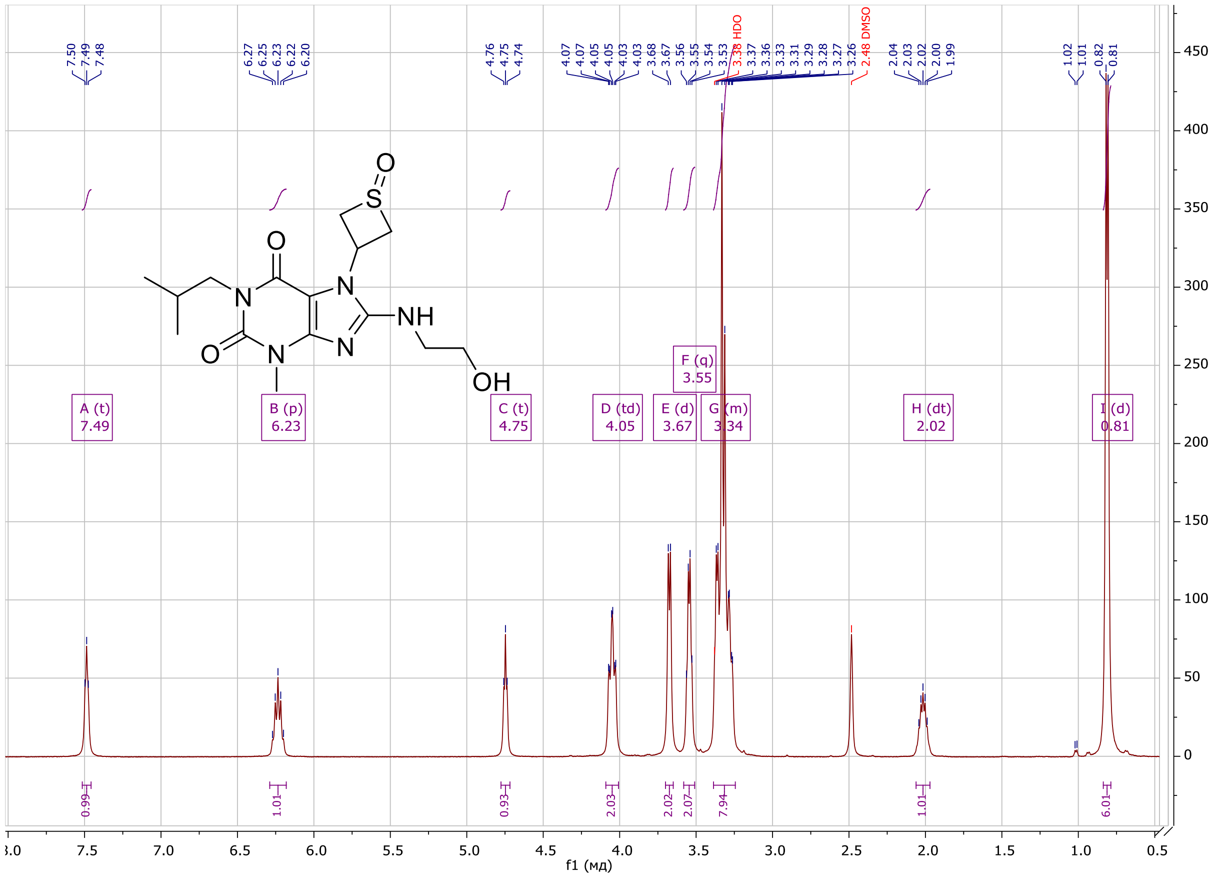

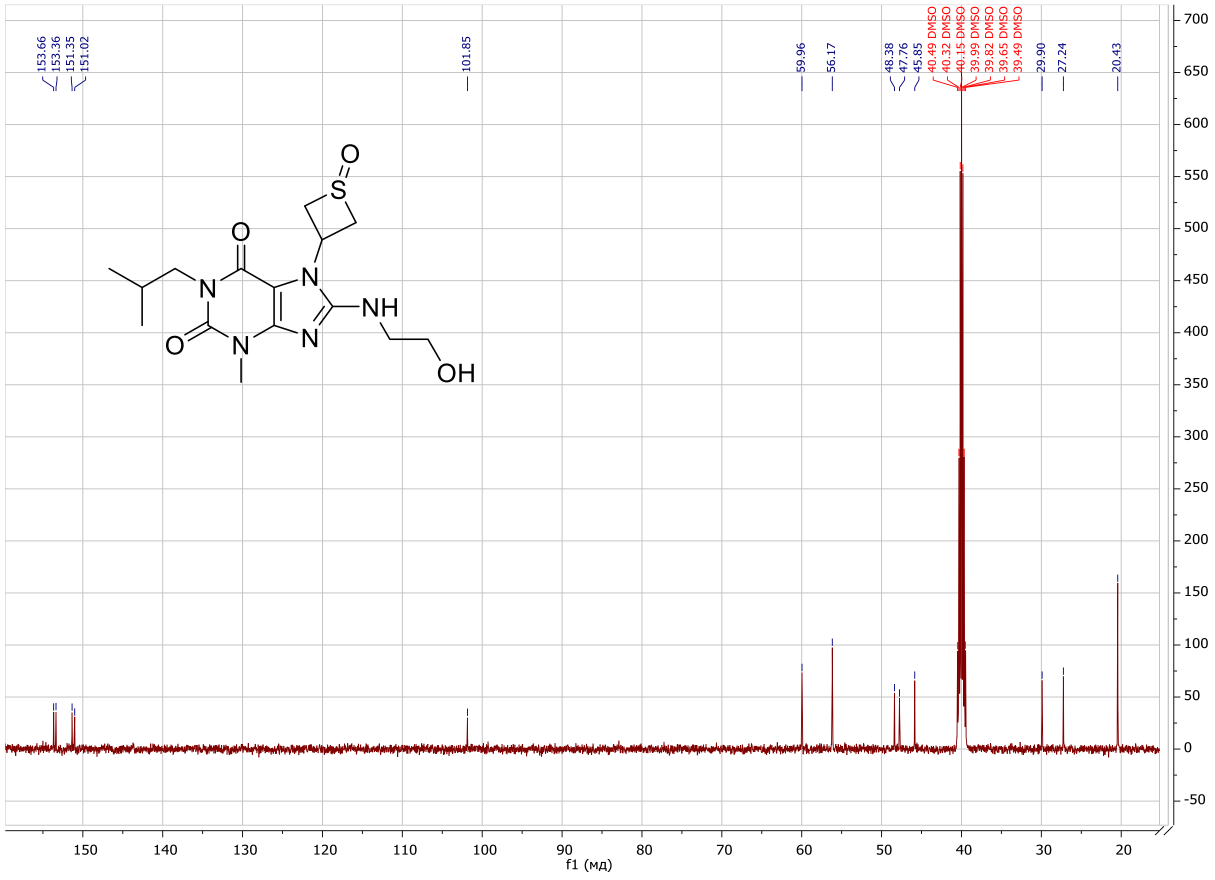

Figure S13: ^{1}H NMR (500 MHz, DMSO-d_6) and ^{13}C NMR (125 MHz, DMSO-d_6) spectra of **6j**

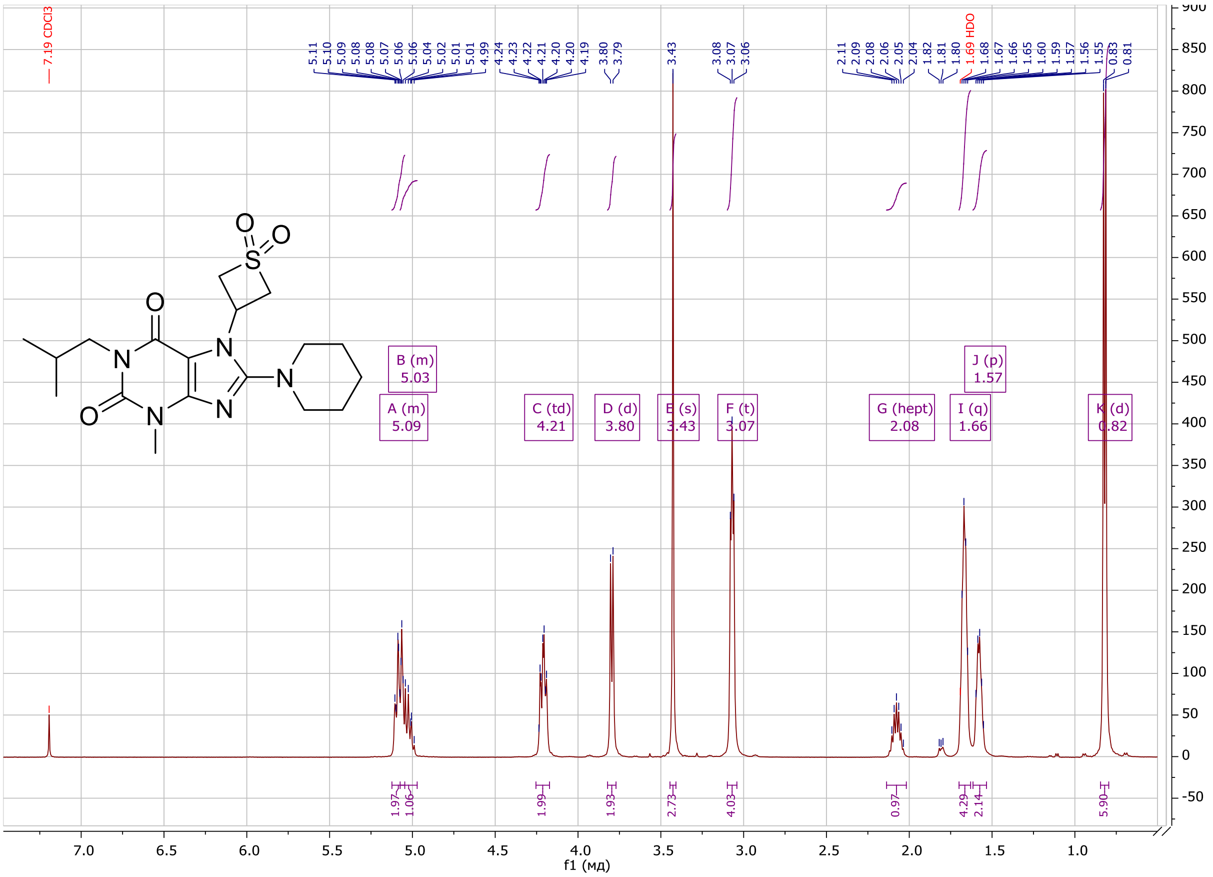

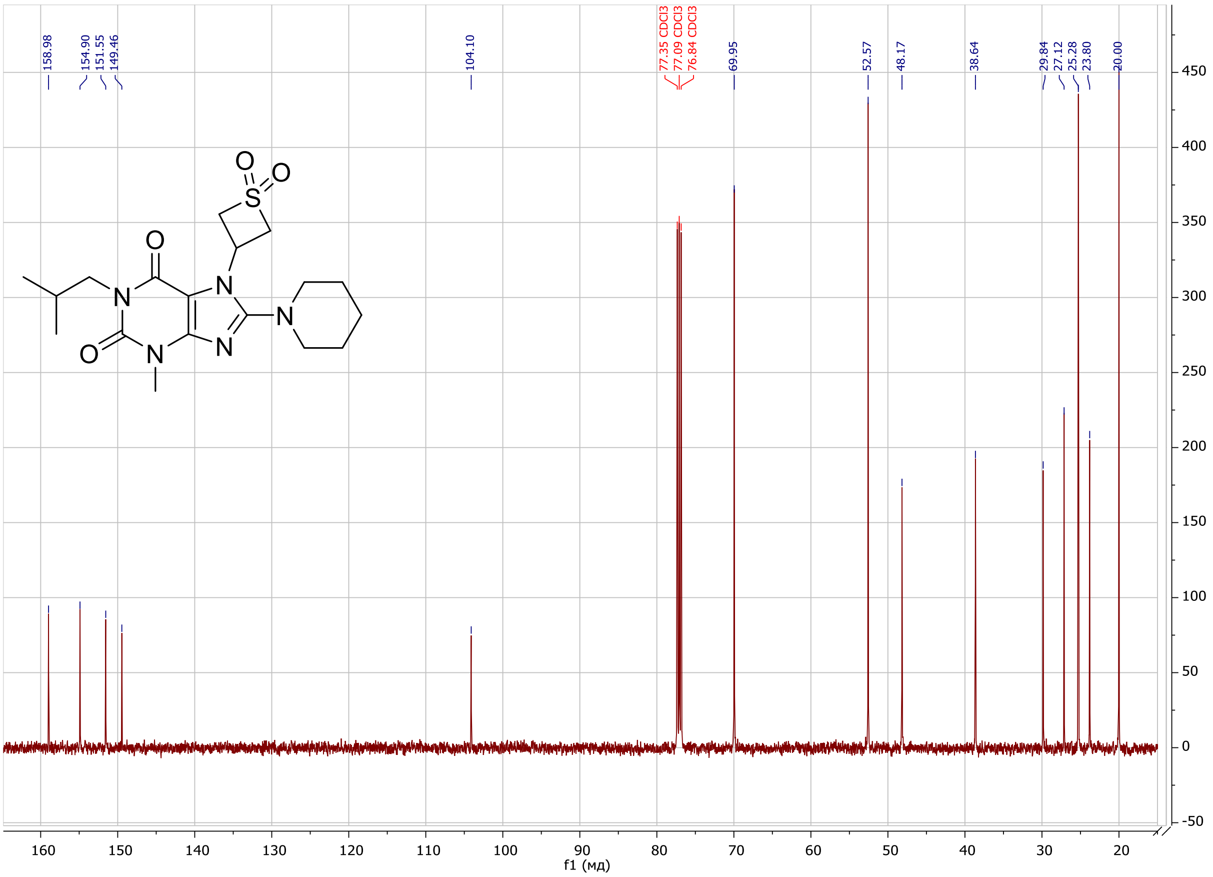

Figure S14: ^{1}H NMR (500 MHz, $CDCl_3$) and ^{13}C NMR (125 MHz, $CDCl_3$) spectra of **6k**

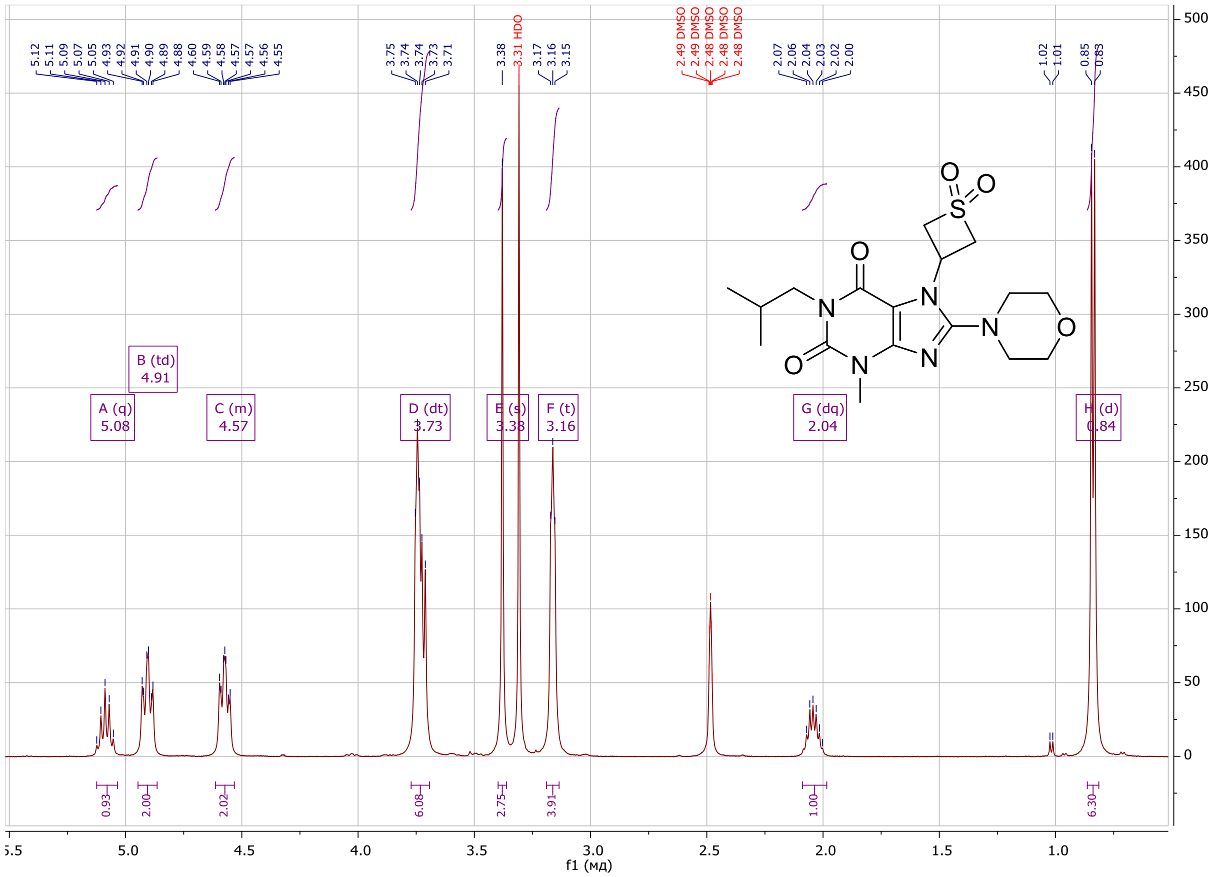

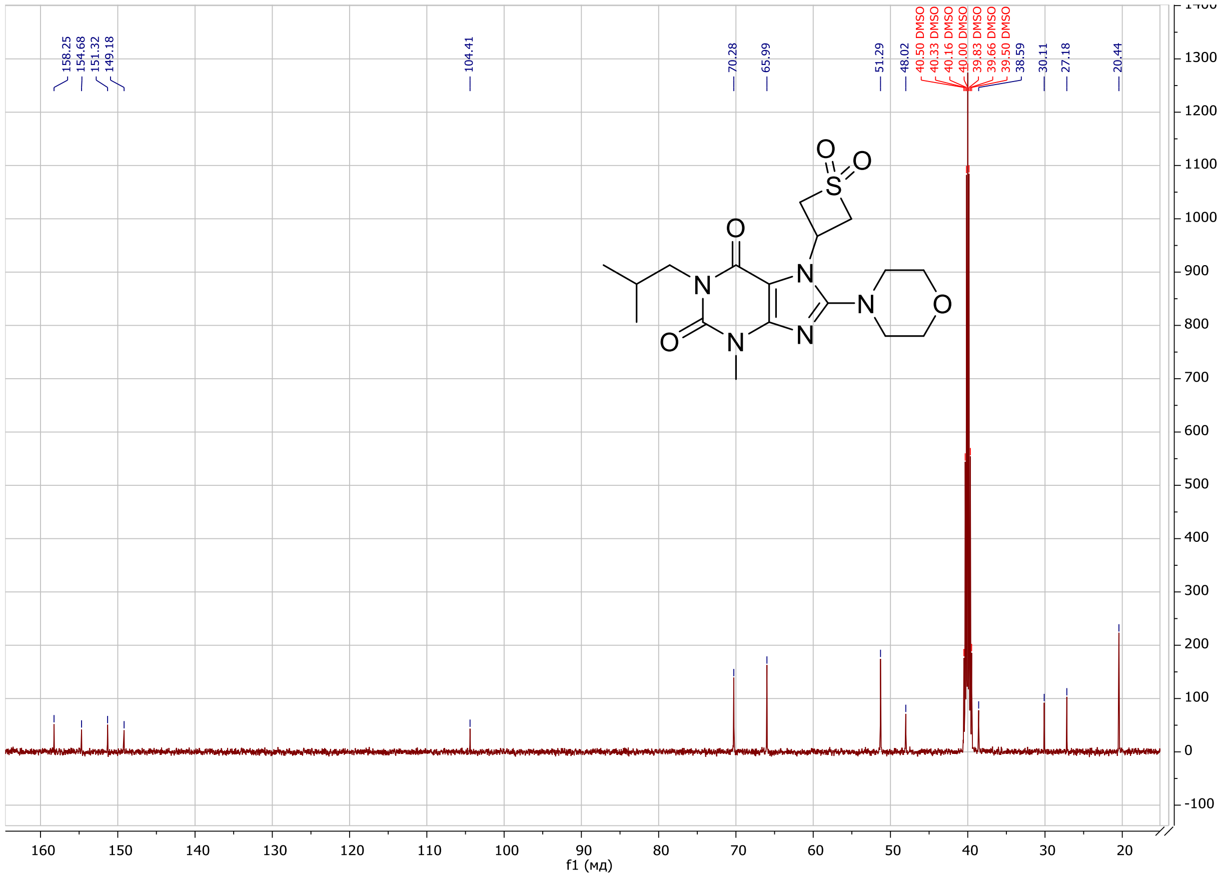

Figure S15: ^{1}H NMR (500 MHz, DMSO-d_6) and ^{13}C NMR (125 MHz, DMSO-d_6) spectra of **6l**

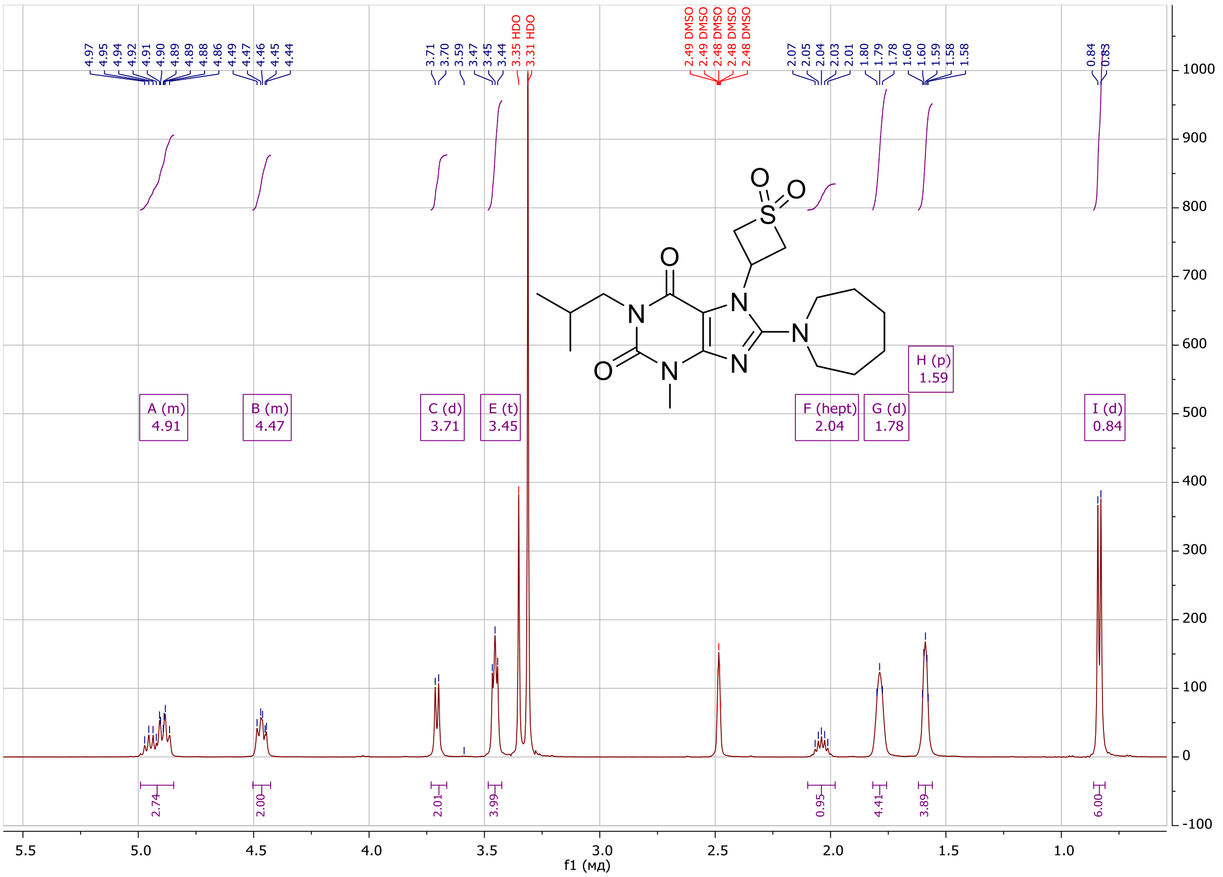

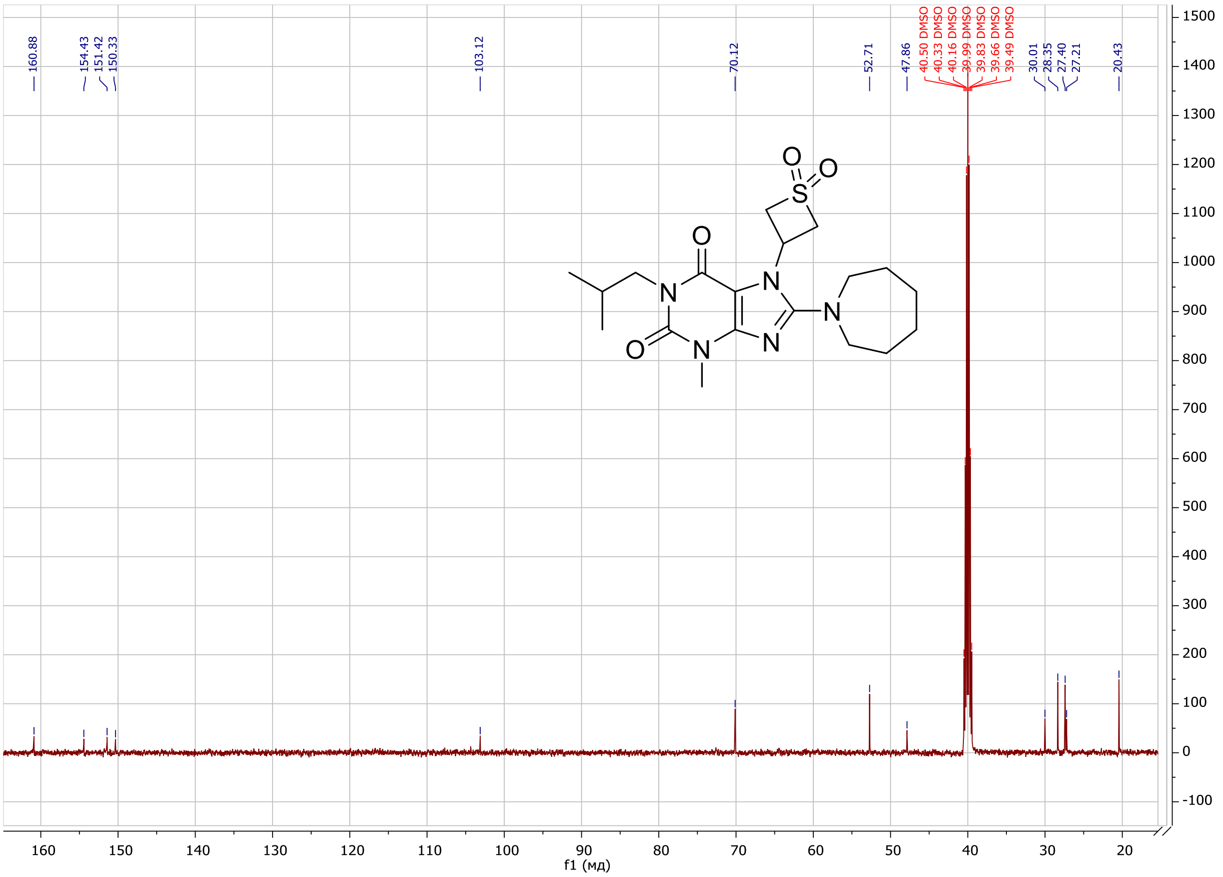

Figure S16: ^{1}H NMR (500 MHz, DMSO-d_6) and ^{13}C NMR (125 MHz, DMSO-d_6) spectra of **6m**

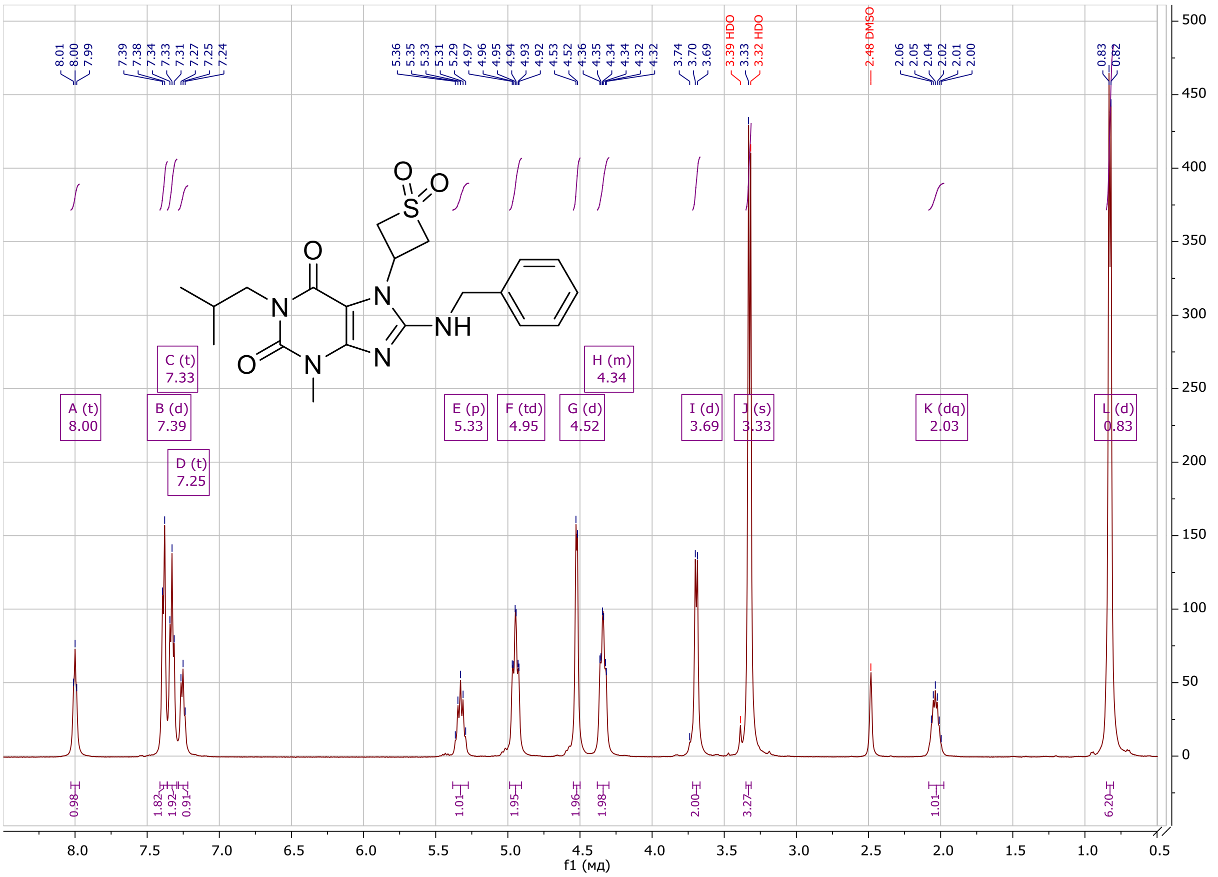

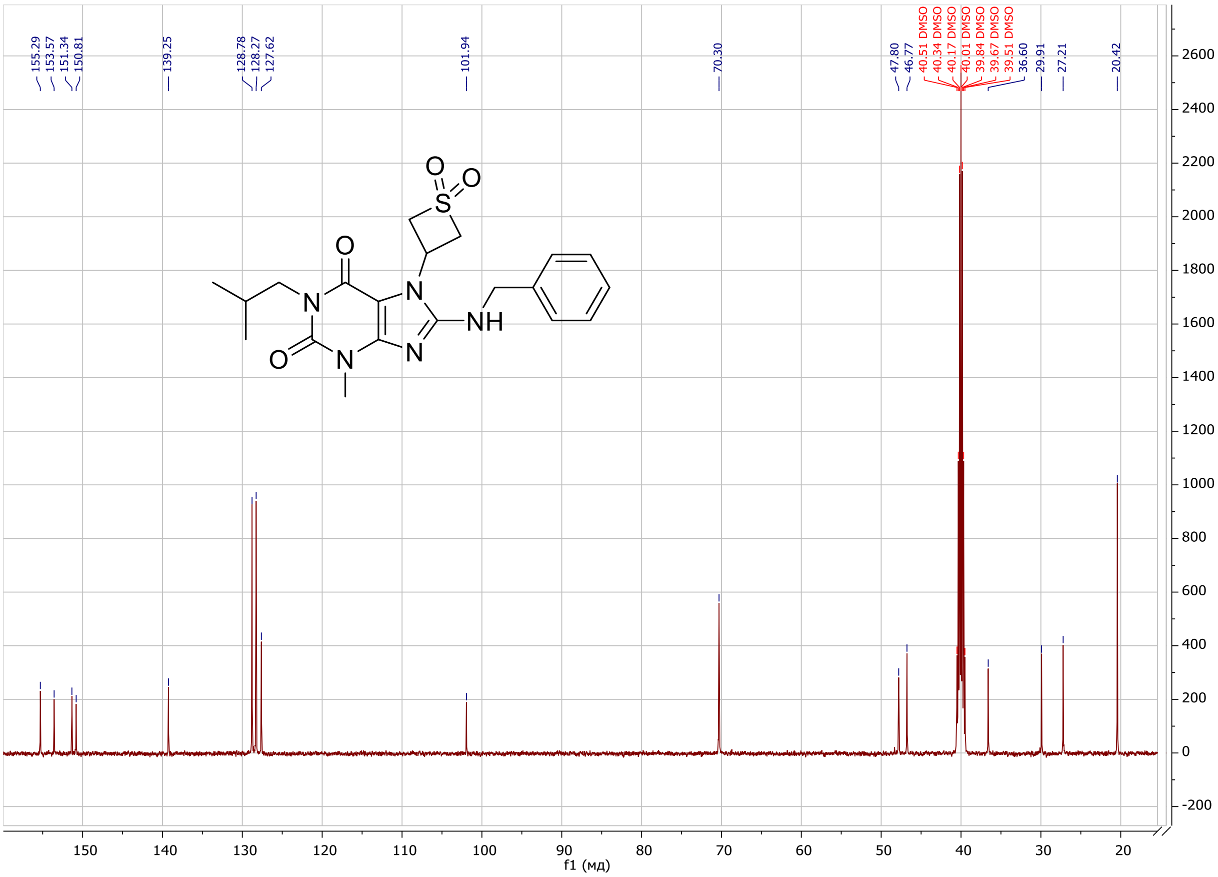

Figure S17: ^{1}H NMR (500 MHz, DMSO-d_6) and ^{13}C NMR (125 MHz, DMSO-d_6) spectra of **6n**

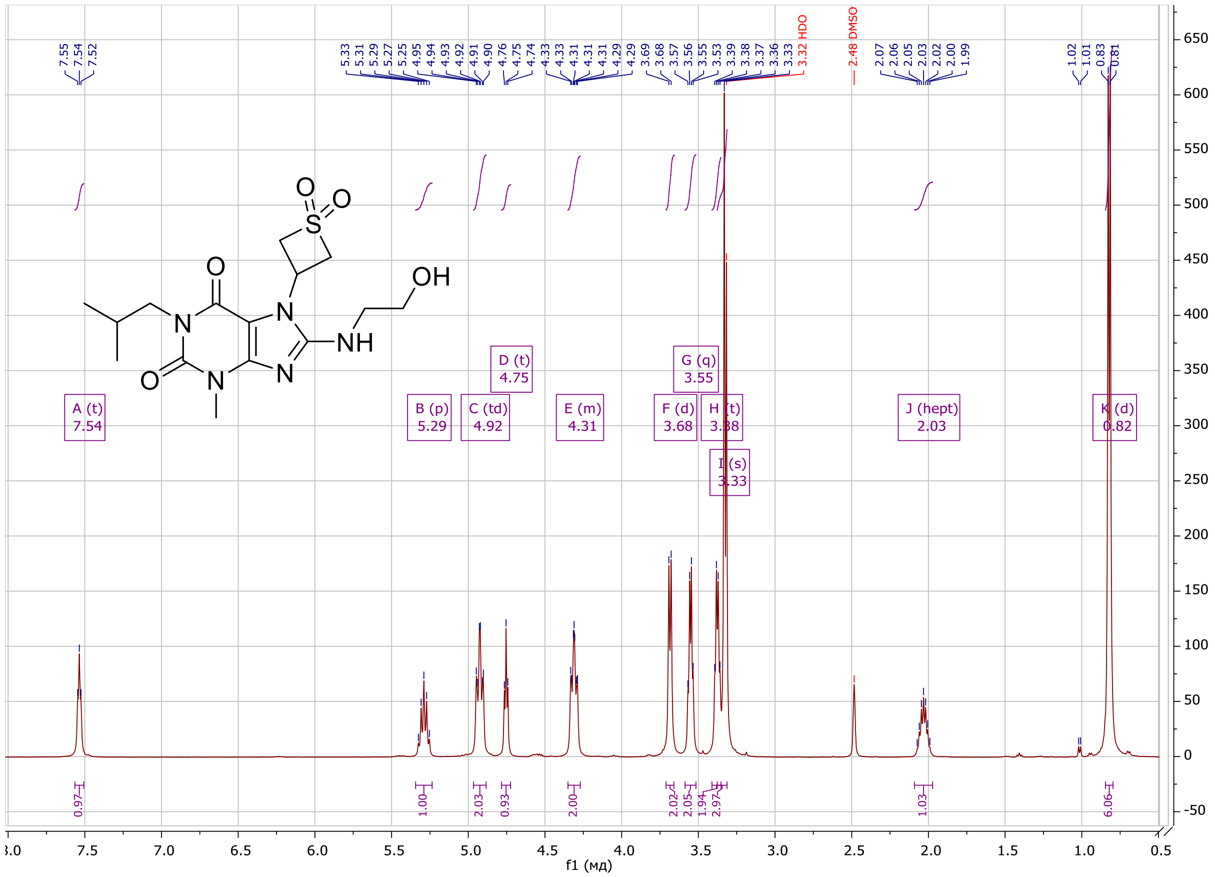

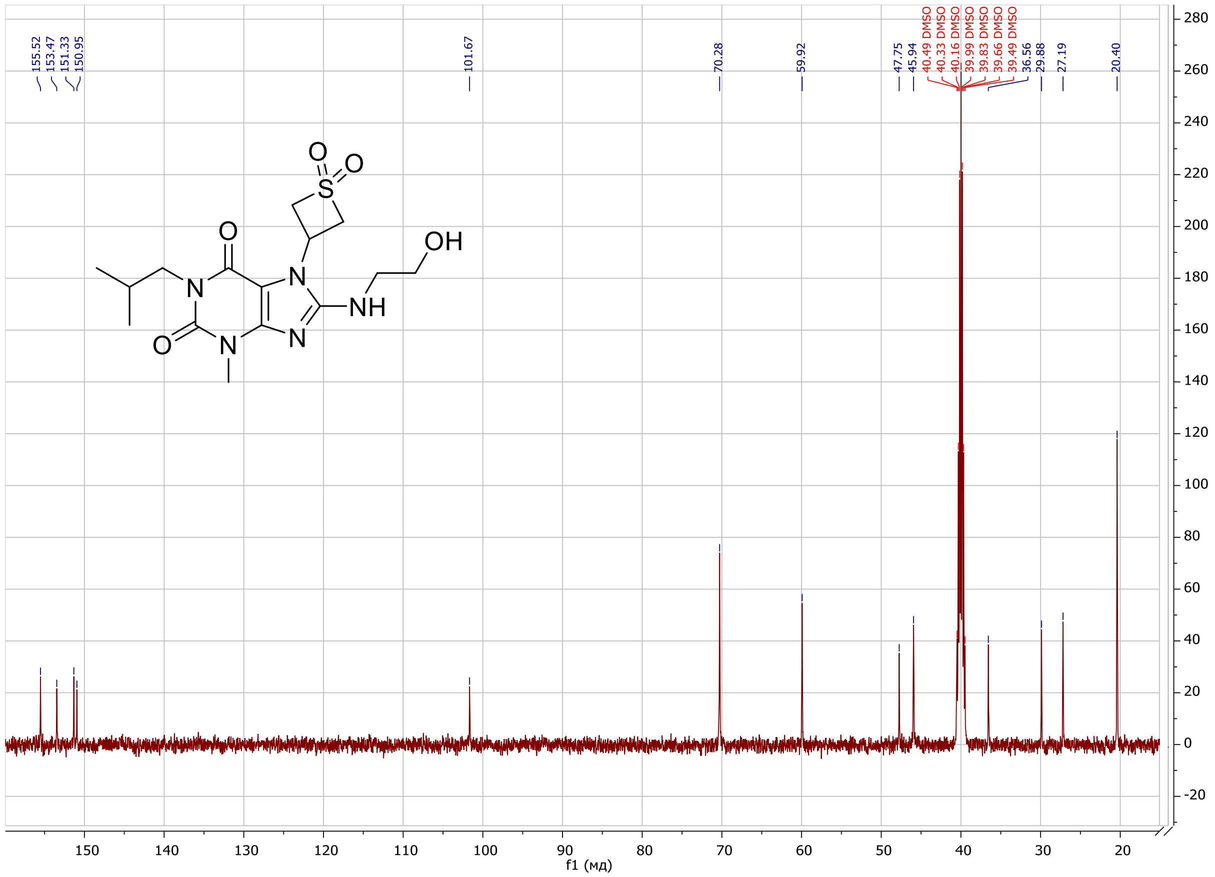

Figure S18: ^{1}H NMR (500 MHz, DMSO-d_6) and ^{13}C NMR (125 MHz, DMSO-d_6) spectra of **6o**

HRMS spectra

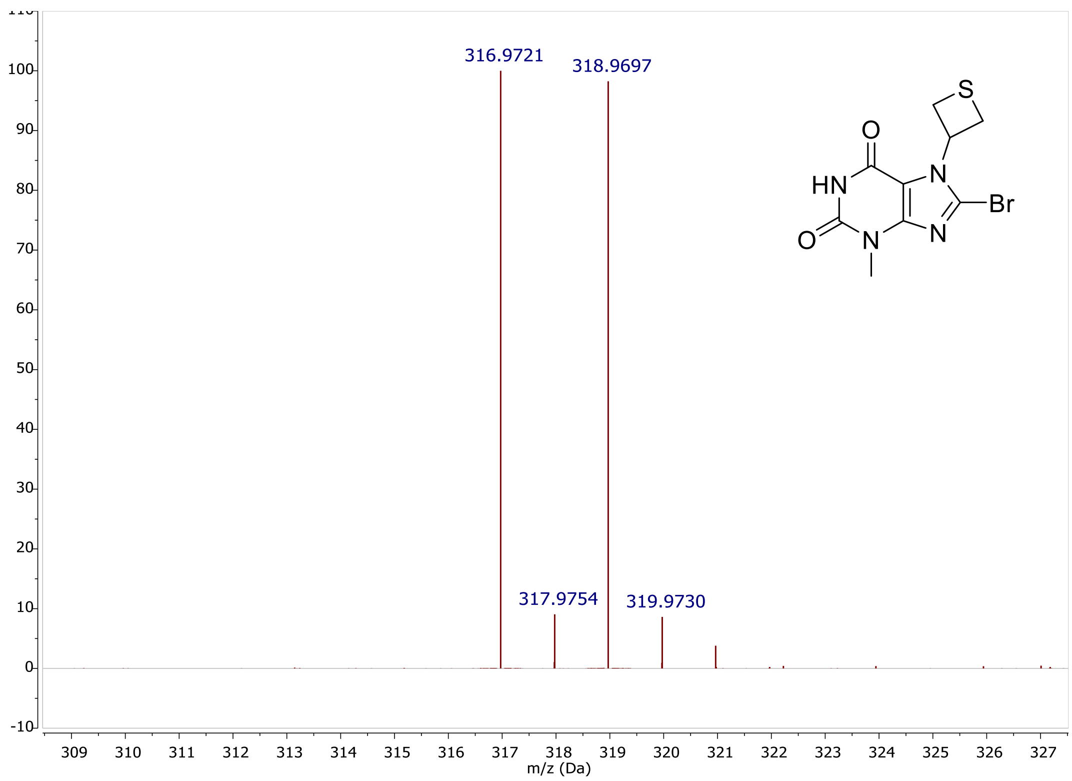

Figure S19: HRMS spectrum of **2**

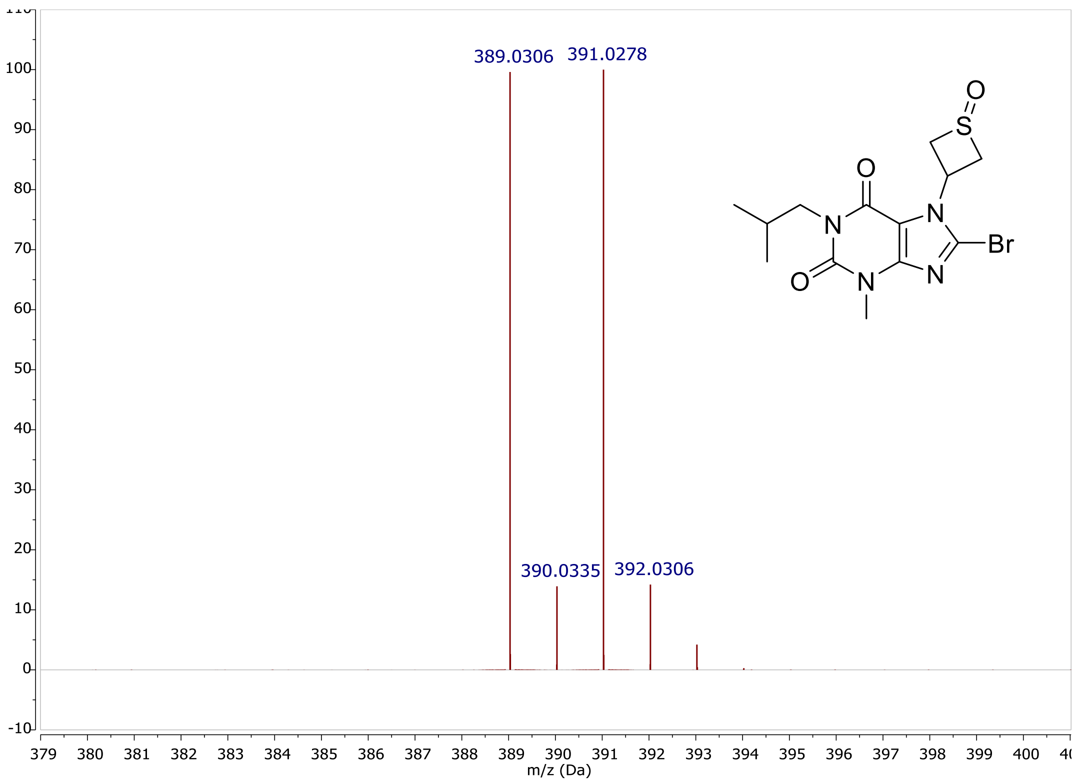

Figure S20: HRMS spectrum of **4**

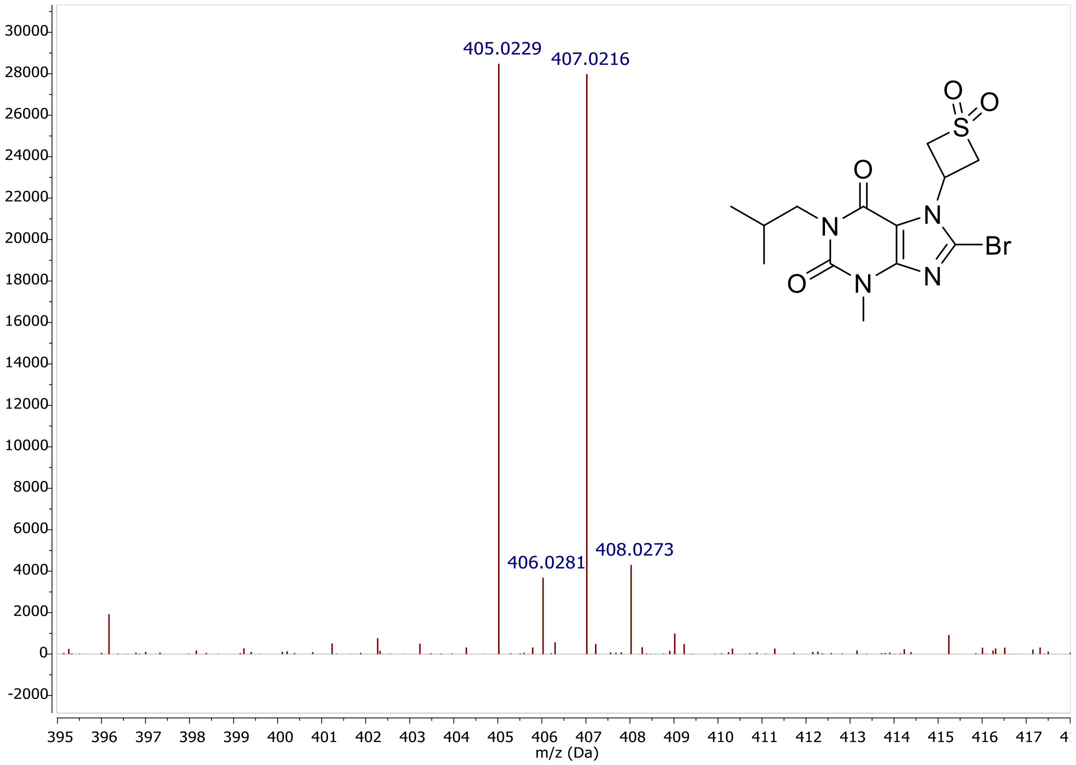

Figure S21: HRMS spectrum of **5**

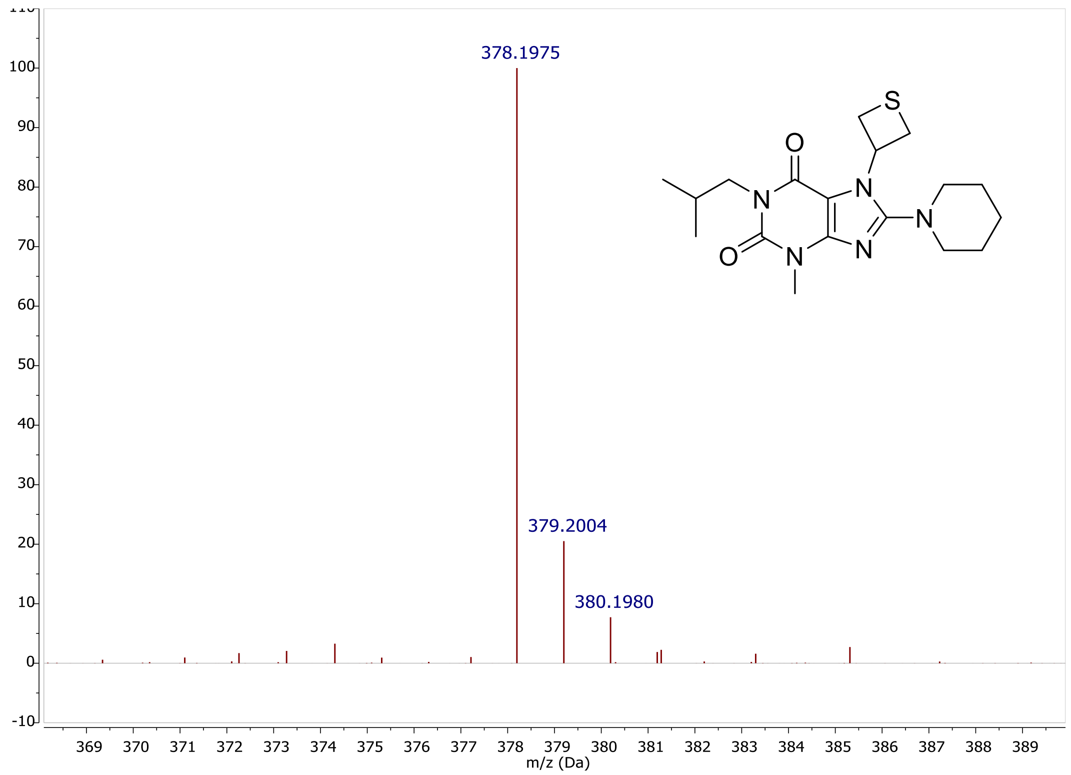

Figure S22: HRMS spectrum of **6a**

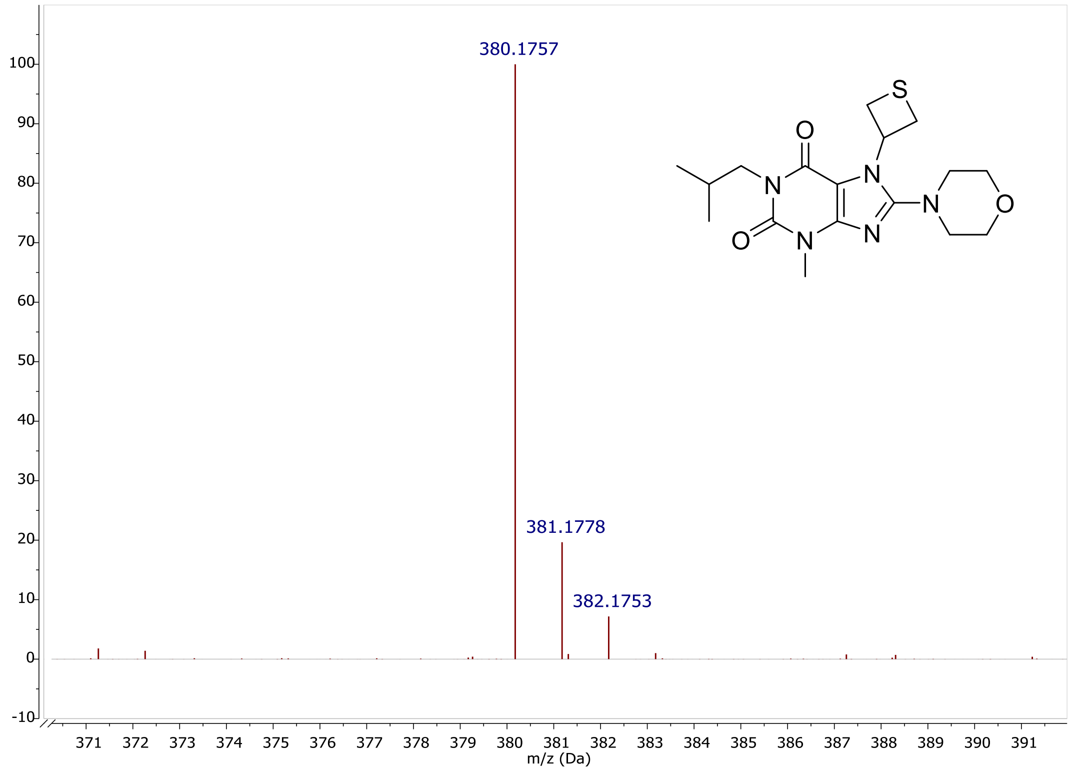

Figure S23: HRMS spectrum of **6b**

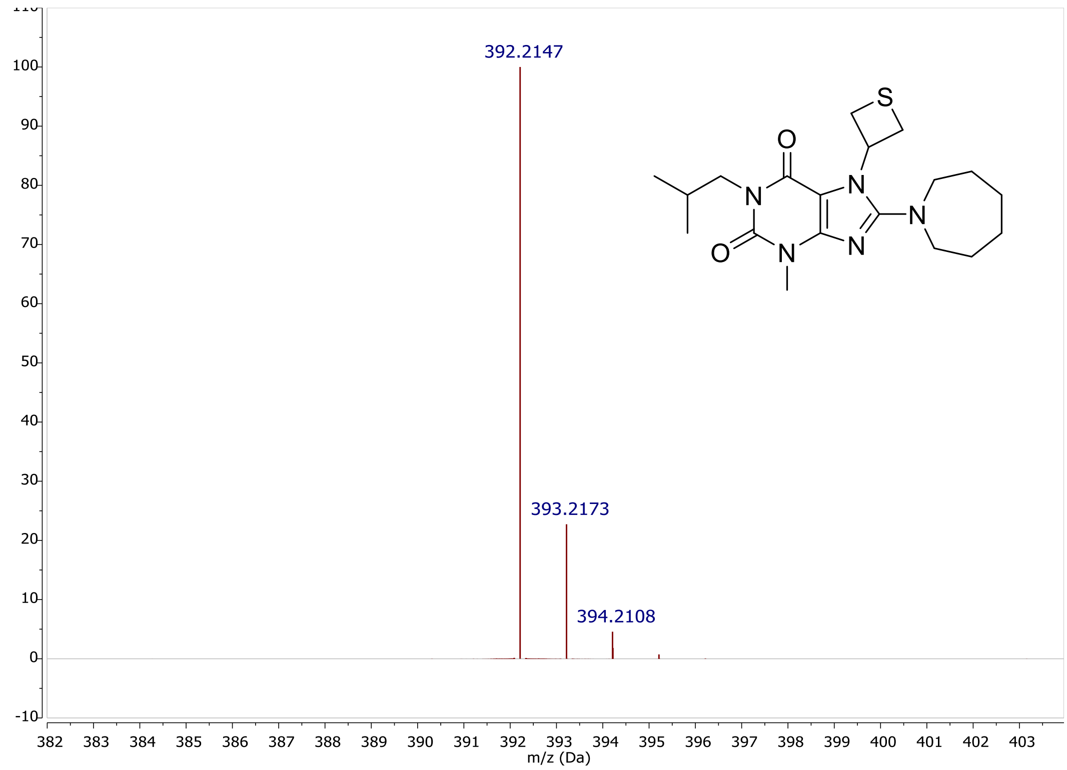

Figure S24: HRMS spectrum of **6c**

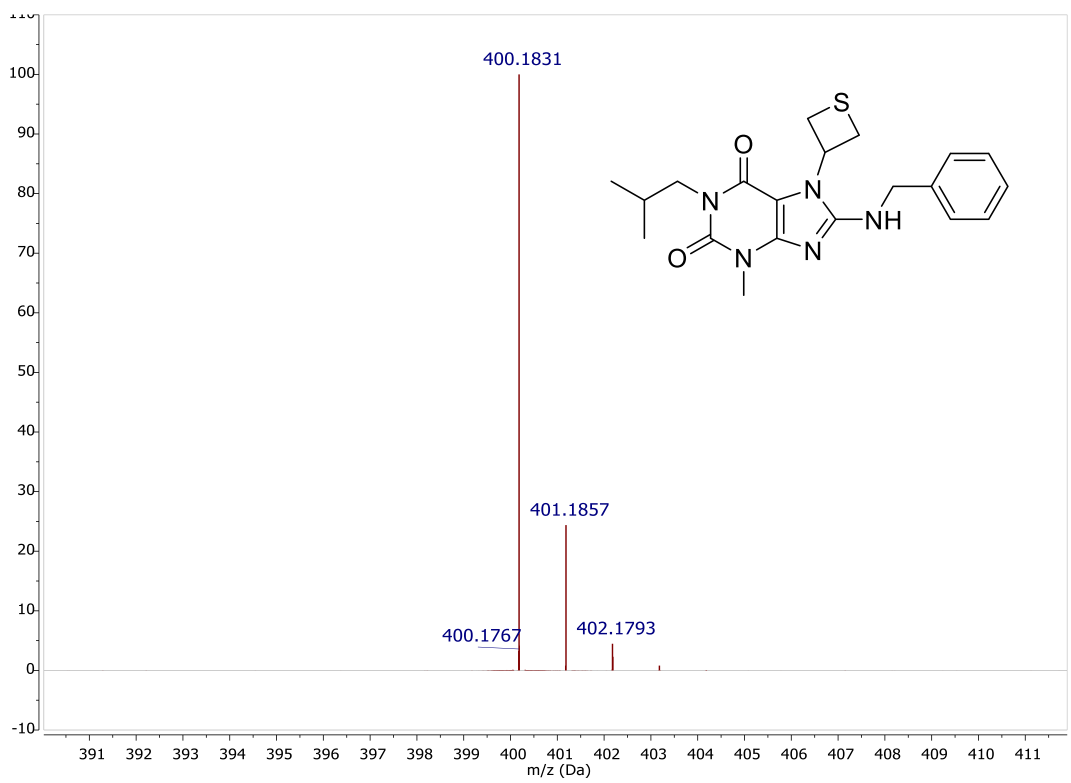

Figure S25: HRMS spectrum of **6d**

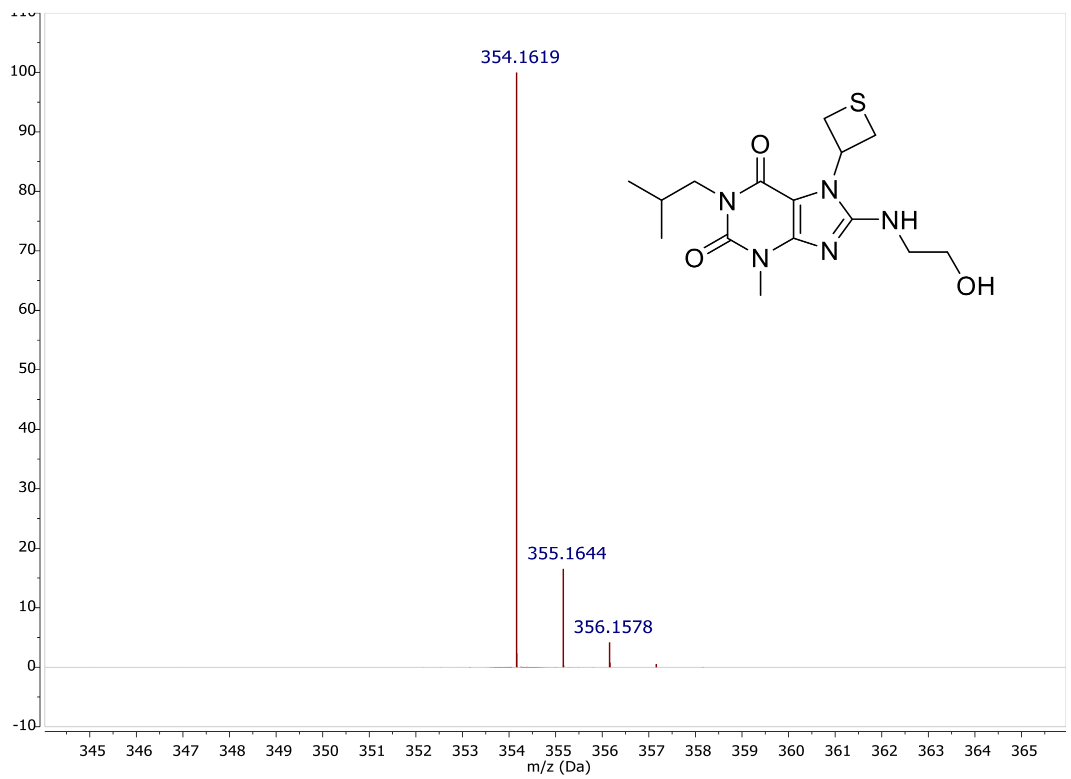

Figure S26: HRMS spectrum of **6e**

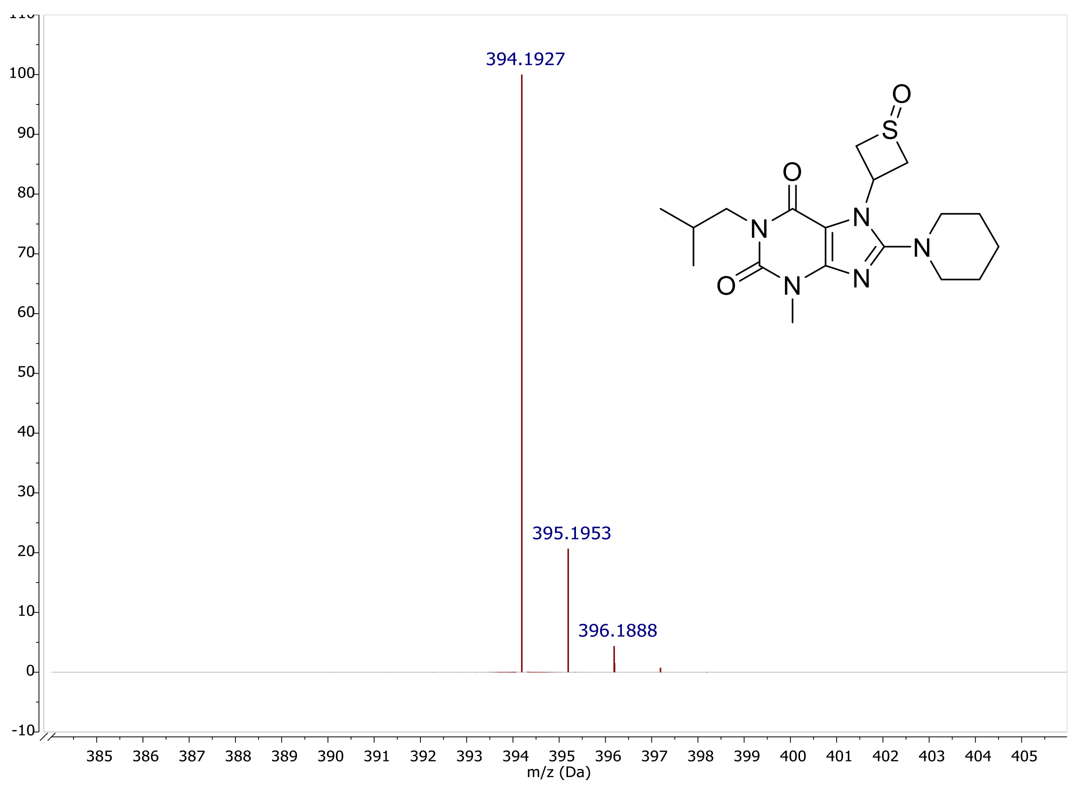

Figure S27: HRMS spectrum of **6f**

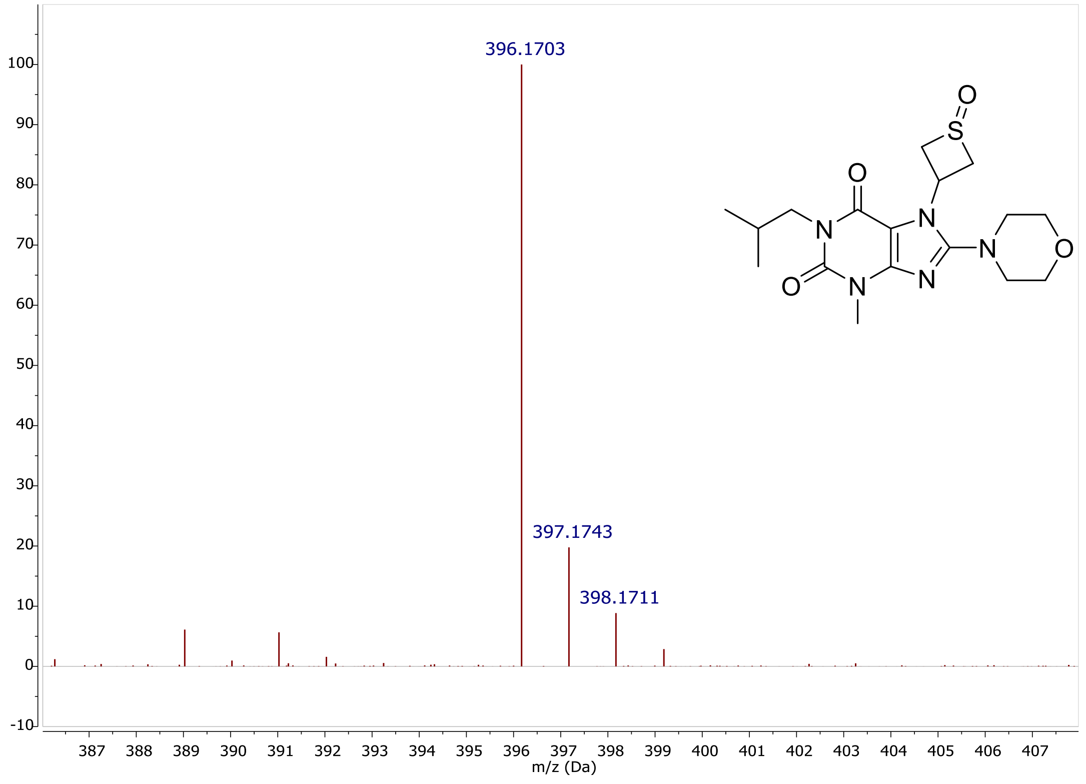

Figure S28: HRMS spectrum of **6g**

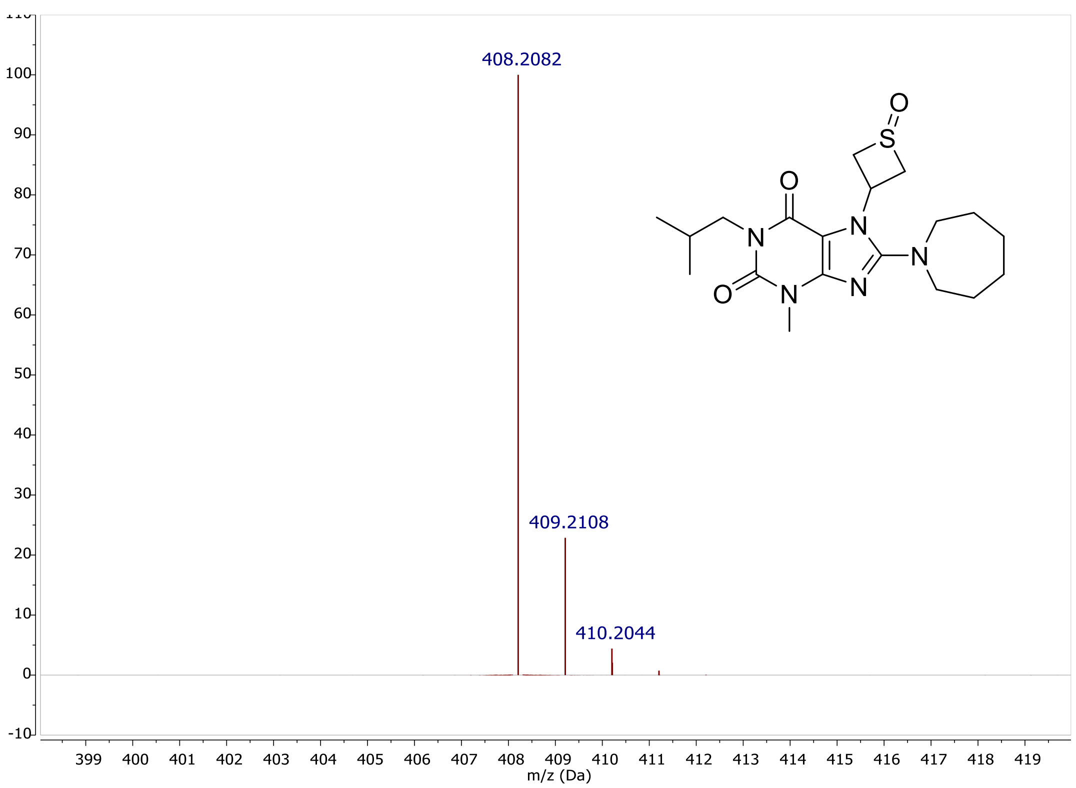

Figure S29: HRMS spectrum of **6h**

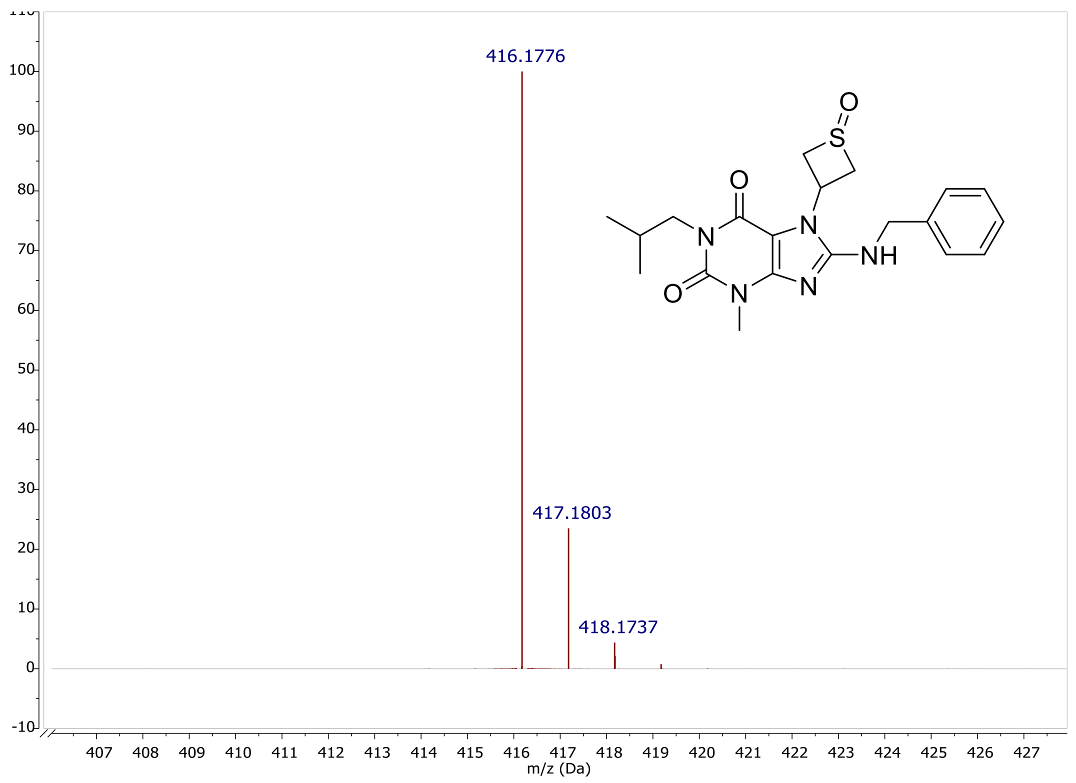

Figure S30: HRMS spectrum of **6i**

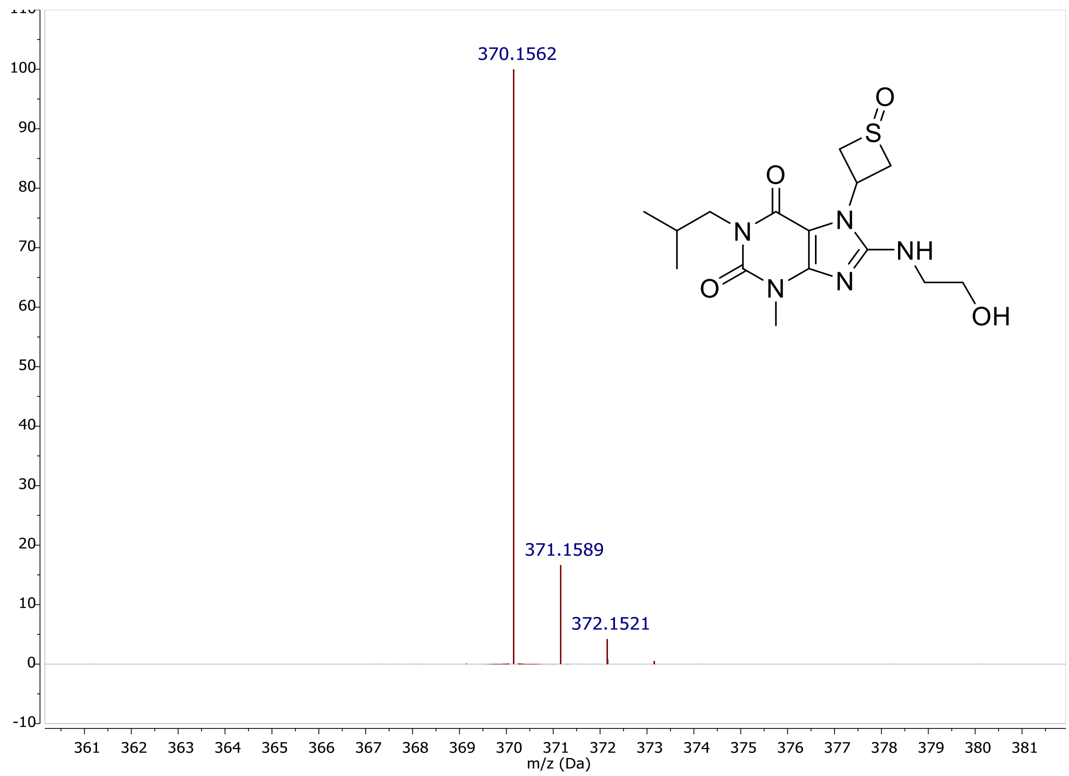

Figure S31: HRMS spectrum of **6j**

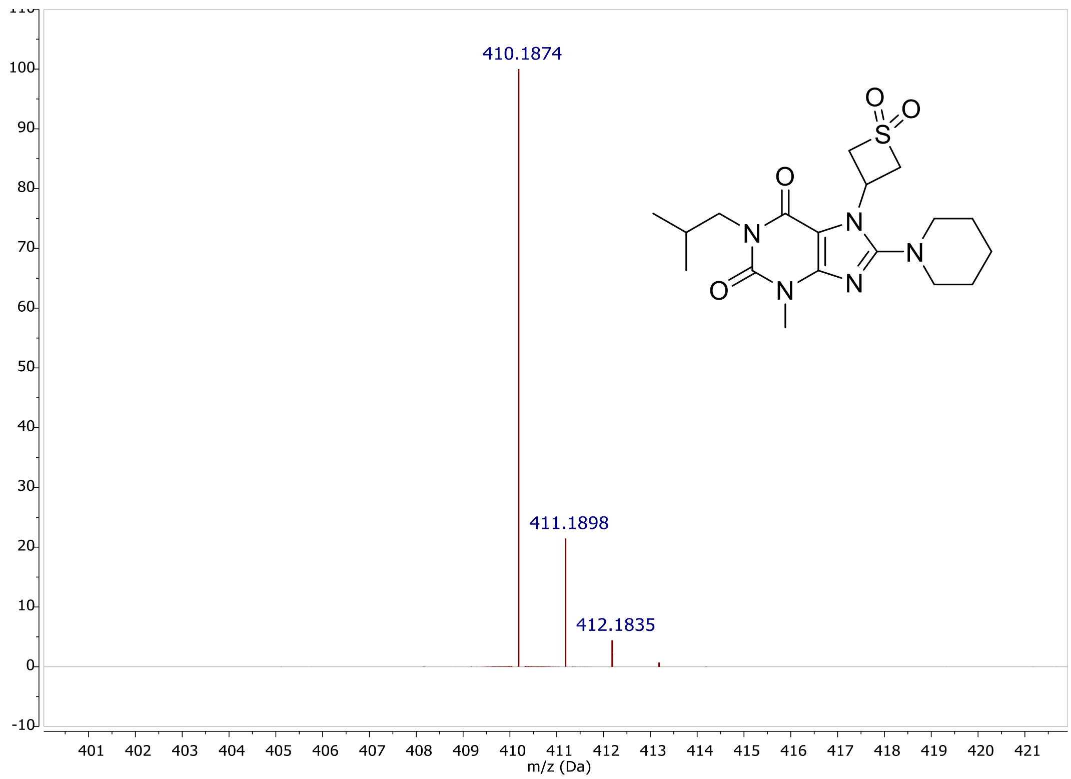

Figure S32: HRMS spectrum of **6k**

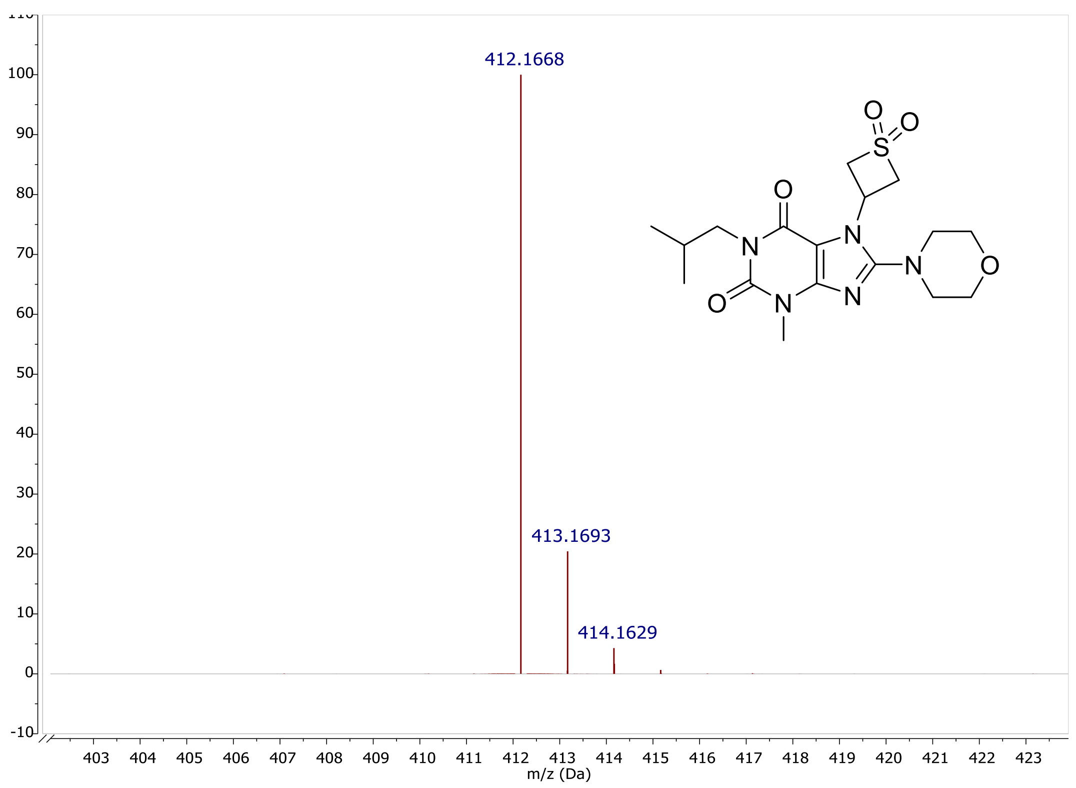

Figure S33: HRMS spectrum of **6l**

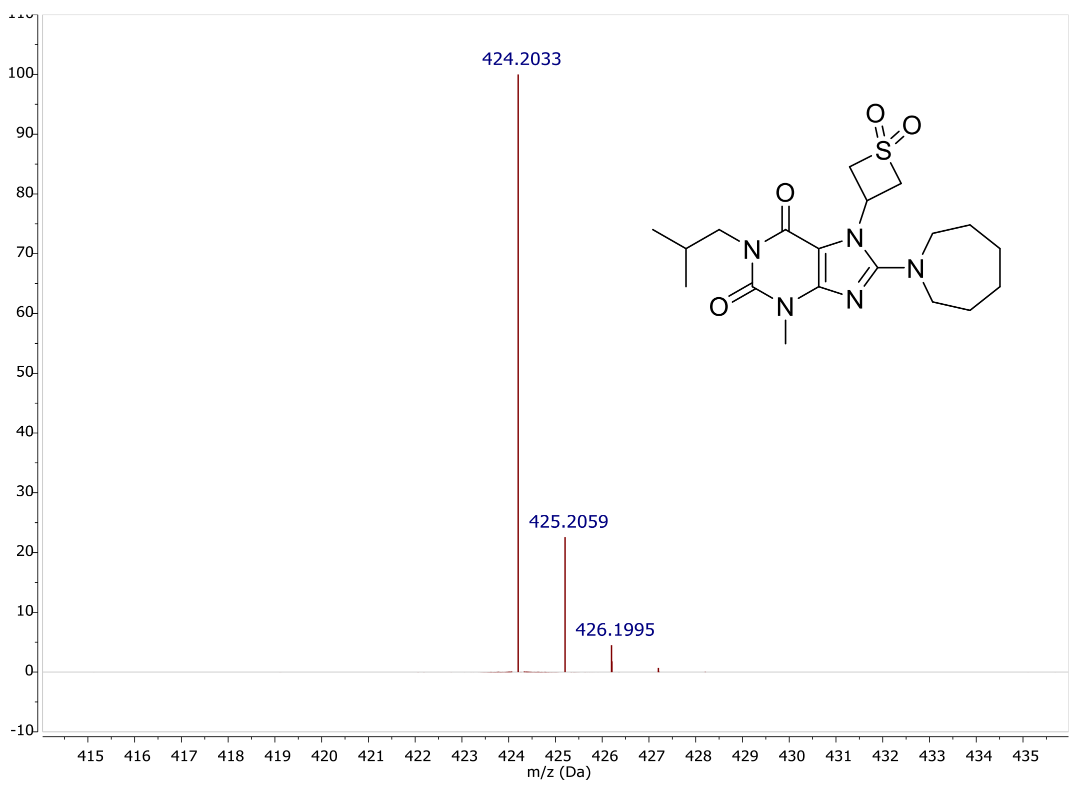

Figure S34: HRMS spectrum of **6m**

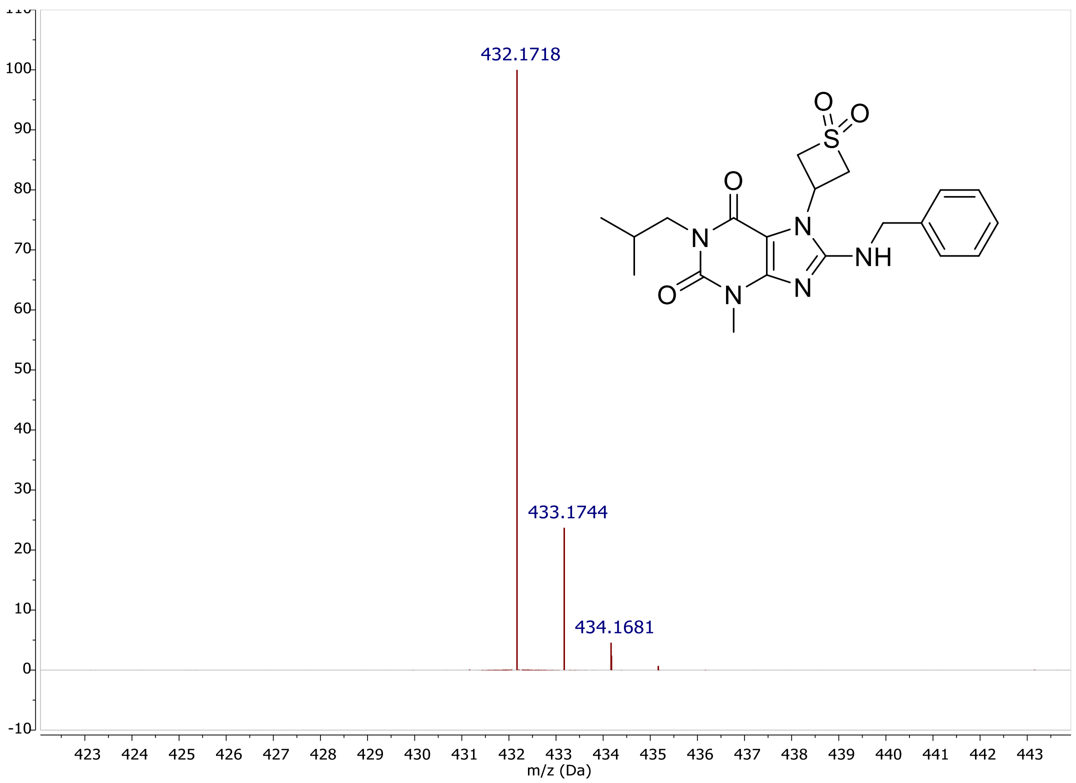

Figure S35: HRMS spectrum of **6n**

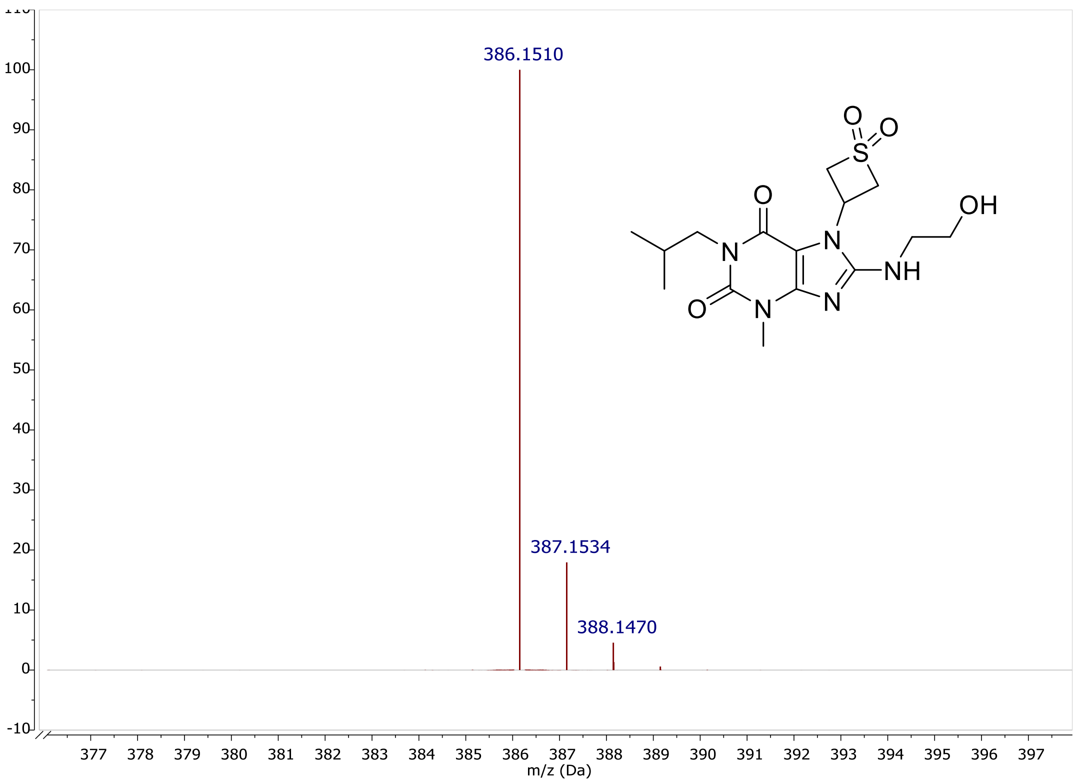

Figure S36: HRMS spectrum of **6o**

Table S1. HRMS data for **2**, **4**, **5**, and **6a–o**.

Cmpd	Formula	Ion	*m/z* calcd	*m/z* found	Δ (ppm)
**2**	$C_9H_9BrN_4O_2S$	$[M+H]^+$	316.9703	316.9721	+5.7
			318.9682	318.9697	+4.7
**4**	$C_{13}H_{17}BrN_4O_3S$	$[M+H]^+$	389.0278	389.0306,	+7.2
			391.0258	91.0278	+5.1
**5***	$C_{13}H_{17}BrN_4O_4S$	$[M+H]^+$	405.0227	405.0229	+0.5
			407.0207	407.0216	+2.2
**6a***	$C_{18}H_{27}N_5O_2S$	$[M+H]^+$	378.1959	378.1975	+4.2
**6b***	$C_{17}H_{25}N_5O_3S$	$[M+H]^+$	380.1751	380.1757	+1.6
**6c**	$C_{19}H_{29}N_5O_2S$	$[M+H]^+$	392.2115	392.2147	+8.2
**6d**	$C_{20}H_{25}N_5O_2S$	$[M+H]^+$	400.1802	400.1831	+7.2
**6e**	$C_{15}H_{23}N_5O_3S$	$[M+H]^+$	354.1595	354.1619	+6.8
**6f**	$C_{18}H_{27}N_5O_3S$	$[M+H]^+$	394.1908	394.1927	+4.8
**6g***	$C_{17}H_{25}N_5O_4S$	$[M+H]^+$	396.1701	396.1703	+0.5
**6h**	$C_{19}H_{29}N_5O_3S$	$[M+H]^+$	408.2064	408.2082	+4.4
**6i**	$C_{20}H_{25}N_5O_3S$	$[M+H]^+$	416.1751	416.1776	+6.0
**6j**	$C_{15}H_{23}N_5O_4S$	$[M+H]^+$	370.1544	370.1562	+4.9
**6k**	$C_{18}H_{27}N_5O_4S$	$[M+H]^+$	410.1857	410.1874	+4.1
**6l**	$C_{17}H_{25}N_5O_5S$	$[M+H]^+$	412.1650	412.1668	+4.4
**6m**	$C_{19}H_{29}N_5O_4S$	$[M+H]^+$	424.2014	424.2033	+4.5
**6n**	$C_{20}H_{25}N_5O_4S$	$[M+H]^+$	432.1701	432.1718	+3.9
**6o**	$C_{15}H_{23}N_5O_5S$	$[M+H]^+$	386.1493	386.1510	+4.4

** – measurements were performed on an Agilent 6530C Accurate-Mass Q-TOF LC/MS system (Agilent Technologies, Santa Clara, CA, USA) (Dual AJS ESI, positive ion mode).*

All other measurements were performed on an an Orbitrap Elite mass spectrometer (Thermo Fisher Scientific, Waltham, MA, USA) (HESI, positive ion mode). Calcd values refer to the monoisotopic mass of the protonated molecular ion $[M+H]^+$. For Br-containing compounds, both peaks of the $^{79}Br/^{81}Br$ isotopic doublet are listed.

IR spectra

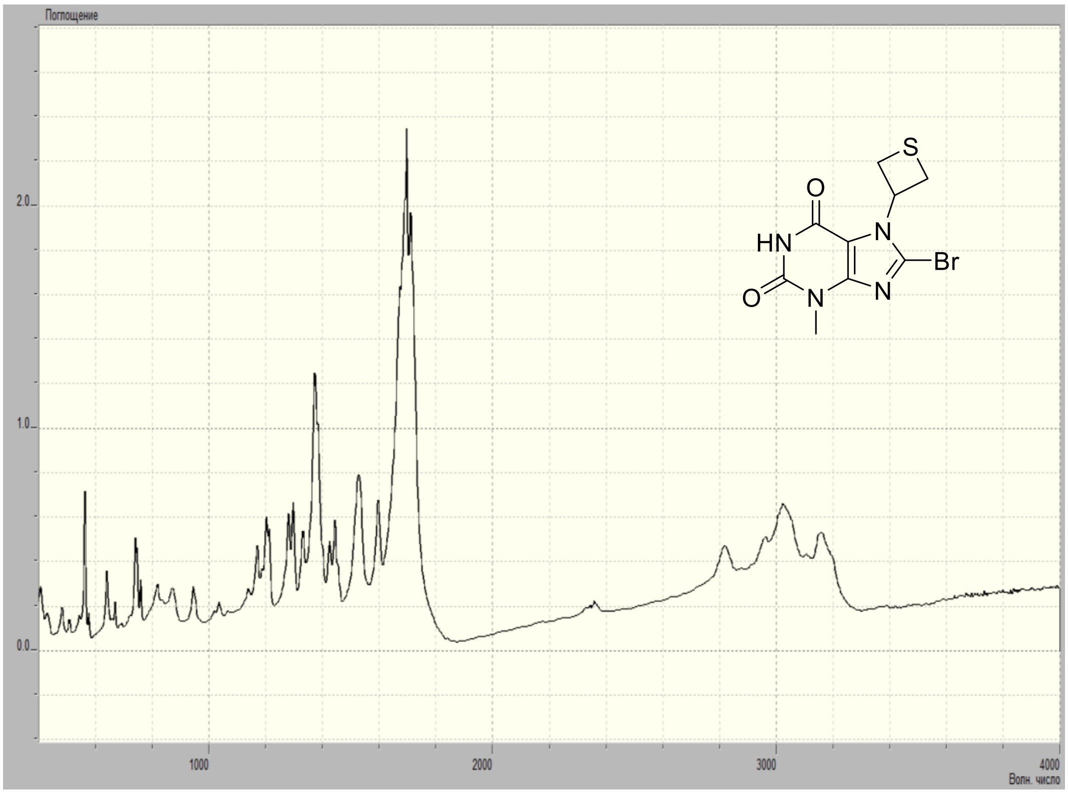

Figure S37: IR spectrum of **2**

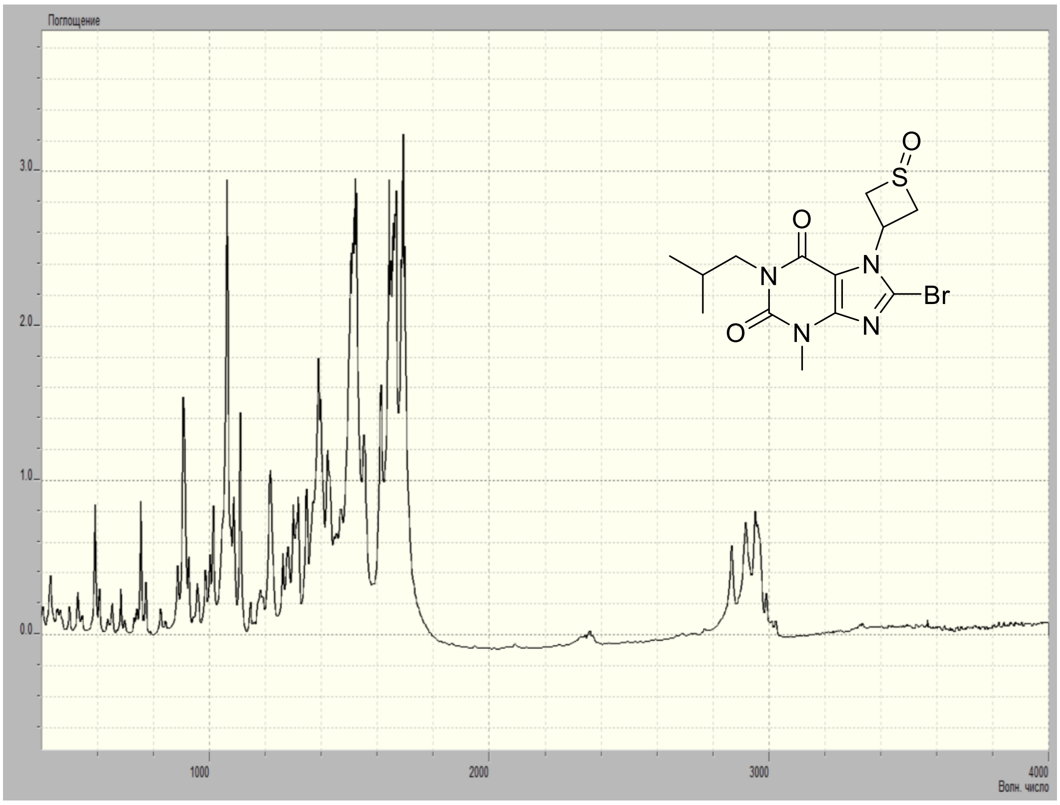

Figure S38: IR spectrum of **4**

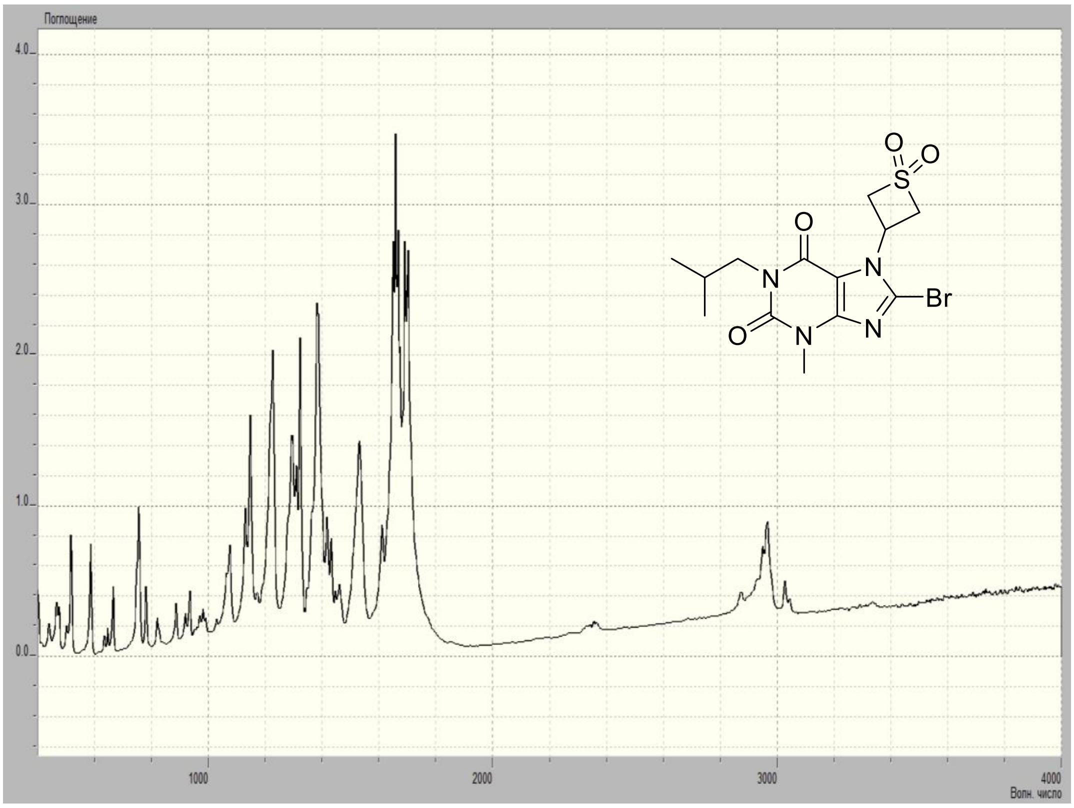

Figure S39: IR spectrum of **5**

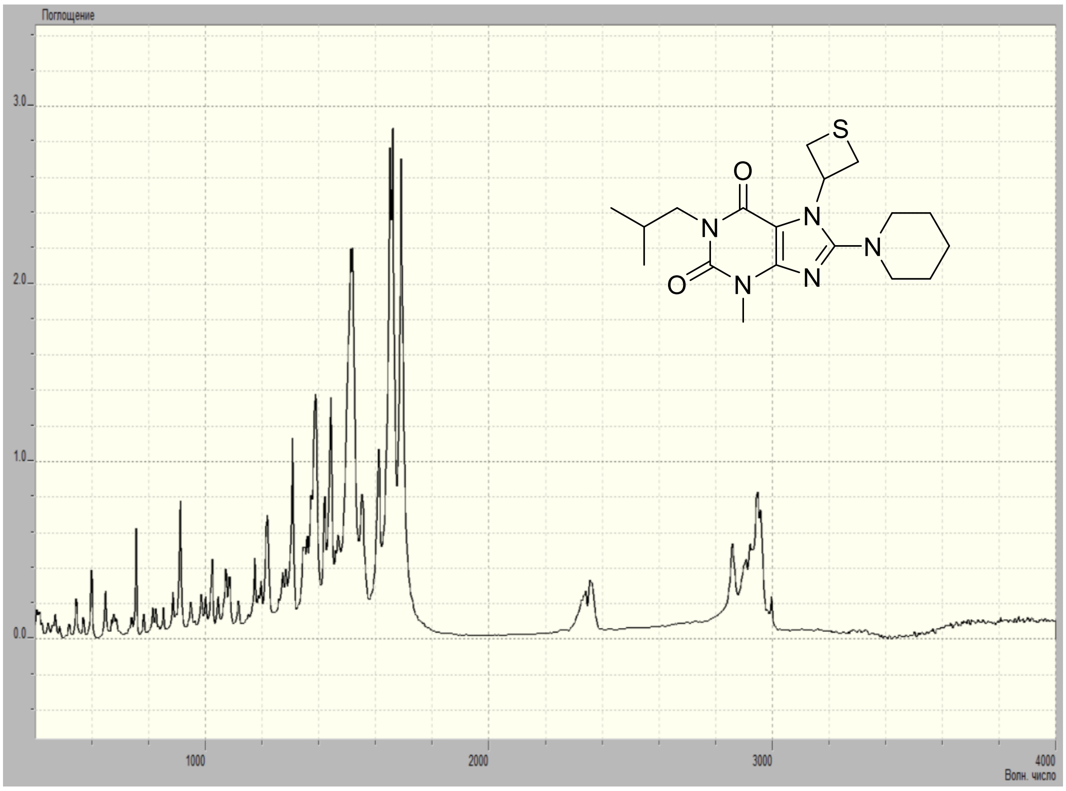

Figure S40: IR spectrum of **6a**

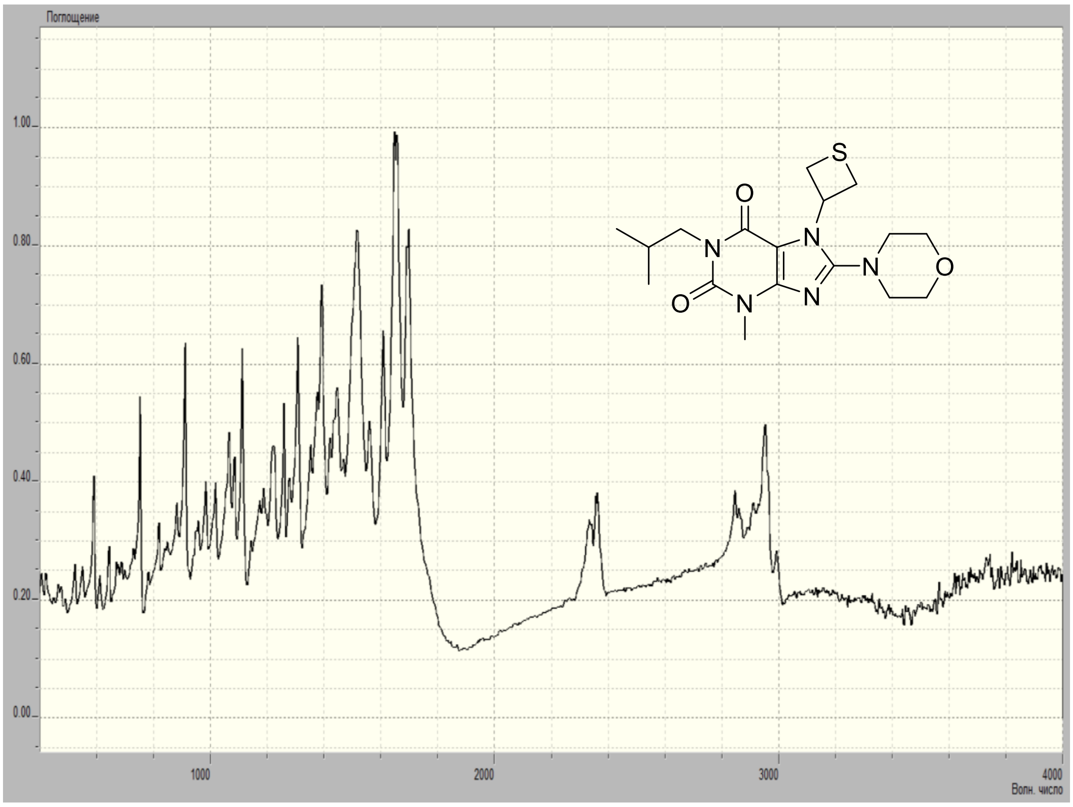

Figure S41: IR spectrum of **6b**

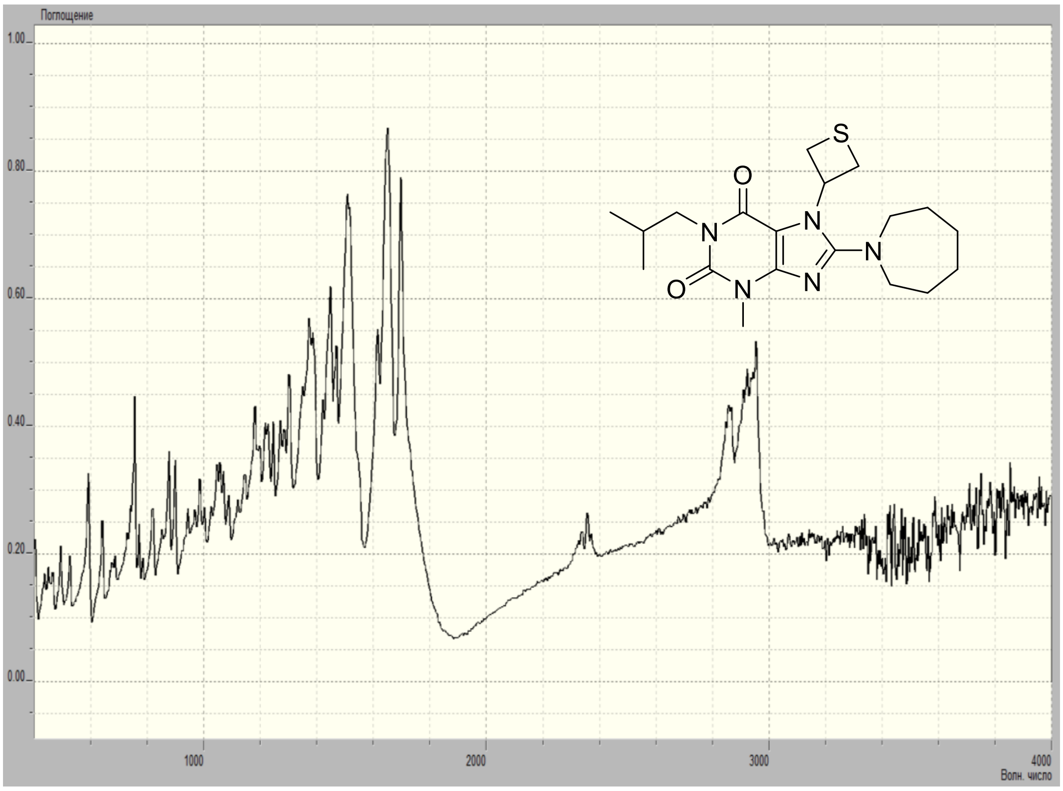

Figure S42: IR spectrum of **6c**

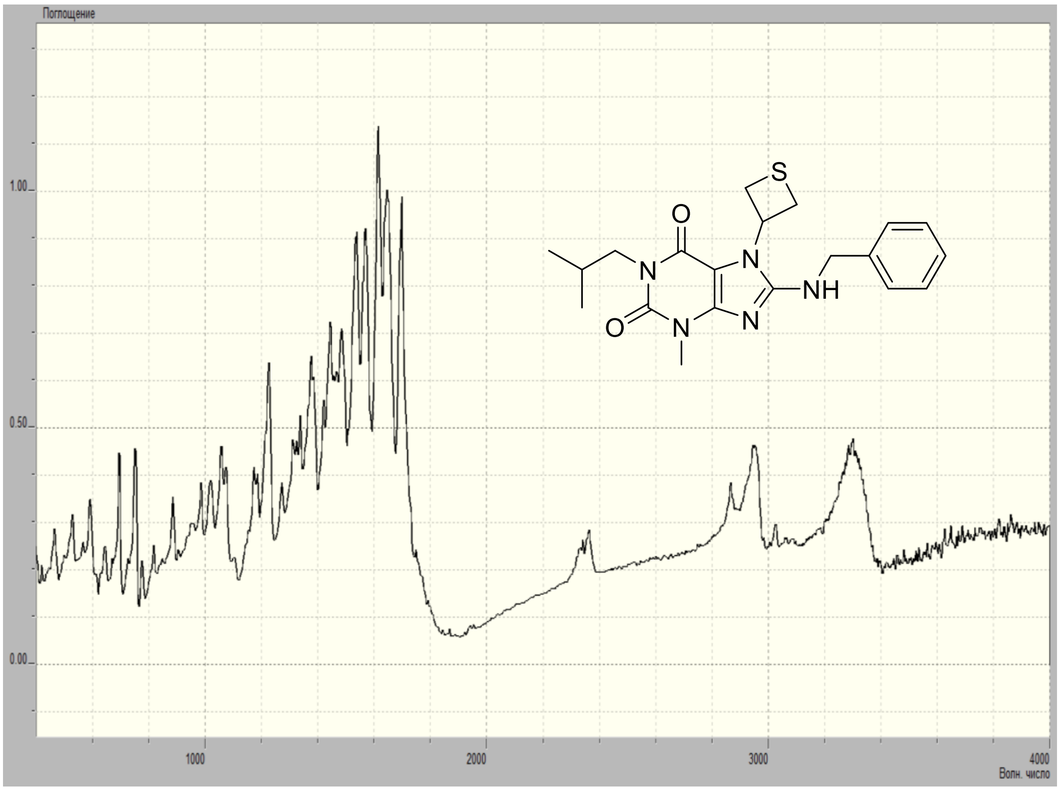

Figure S43: IR spectrum of **6d**

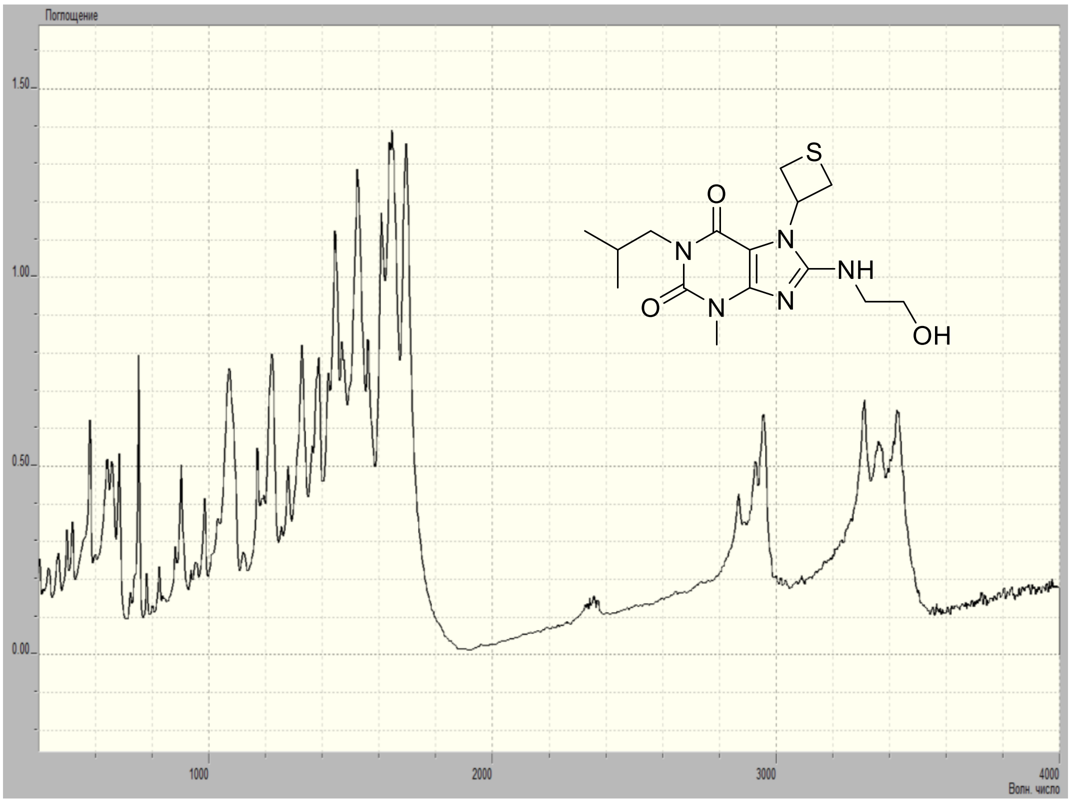

Figure S44: IR spectrum of **6e**

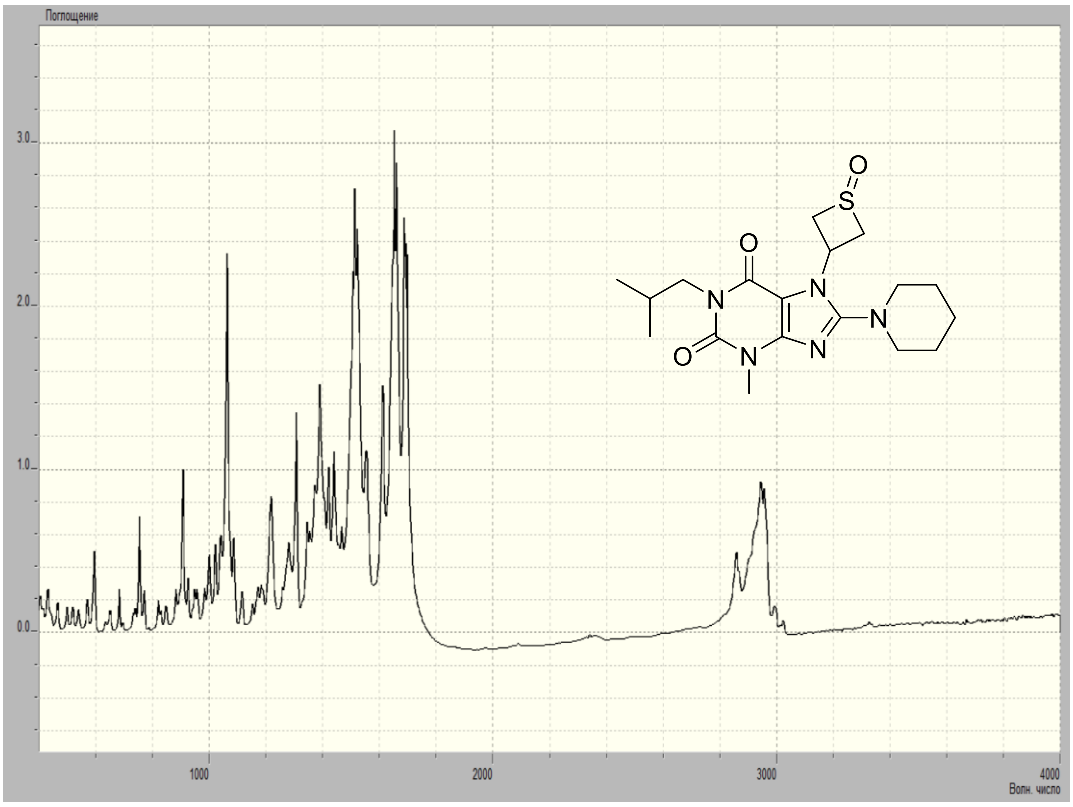

Figure S45: IR spectrum of **6f**

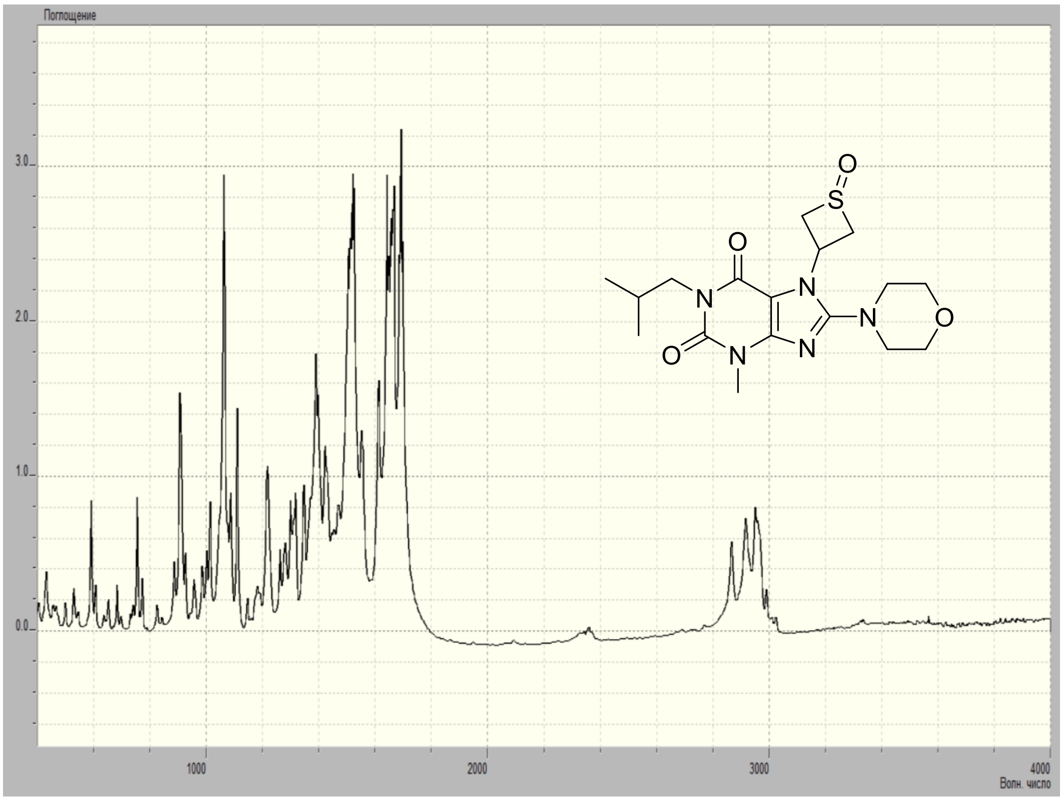

Figure S46: IR spectrum of **6g**

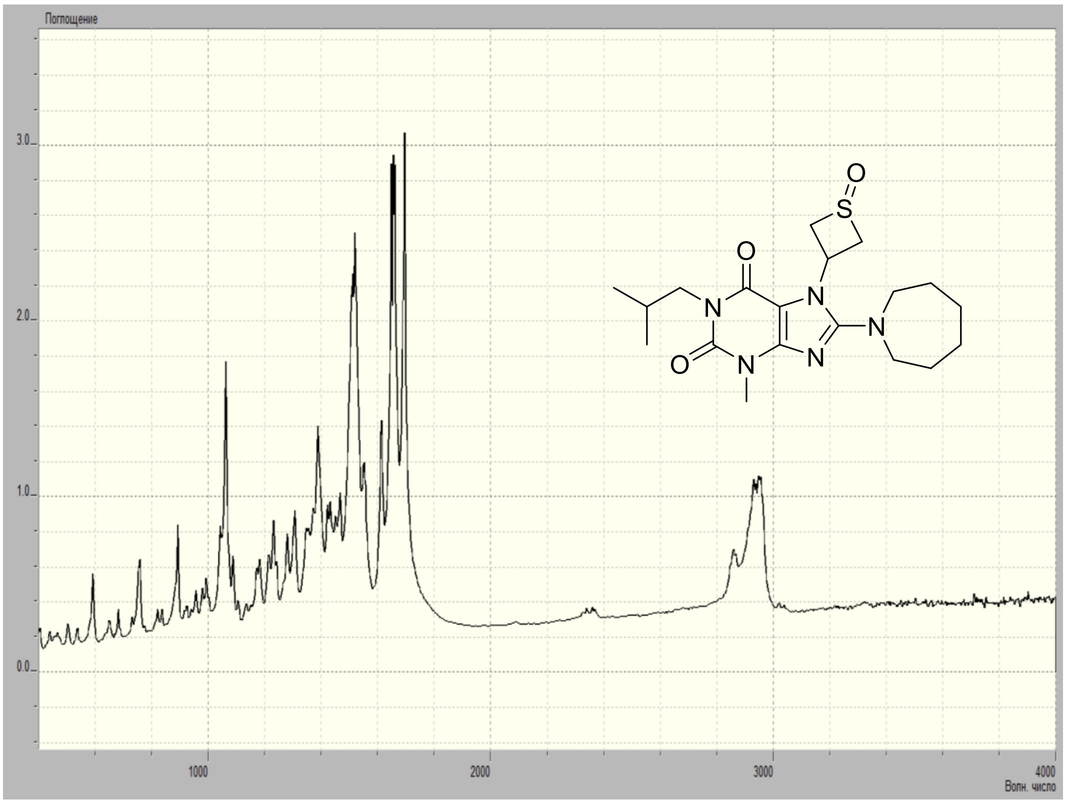

Figure S47: IR spectrum of **6h**

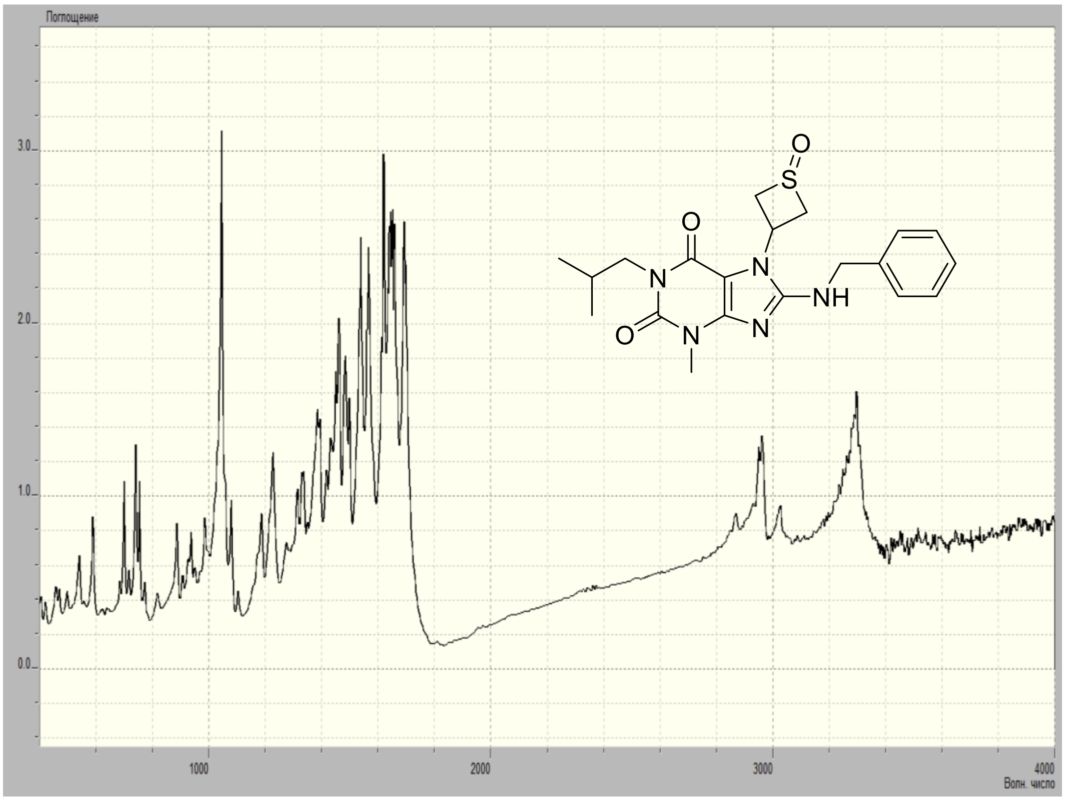

Figure S48: IR spectrum of **6i**

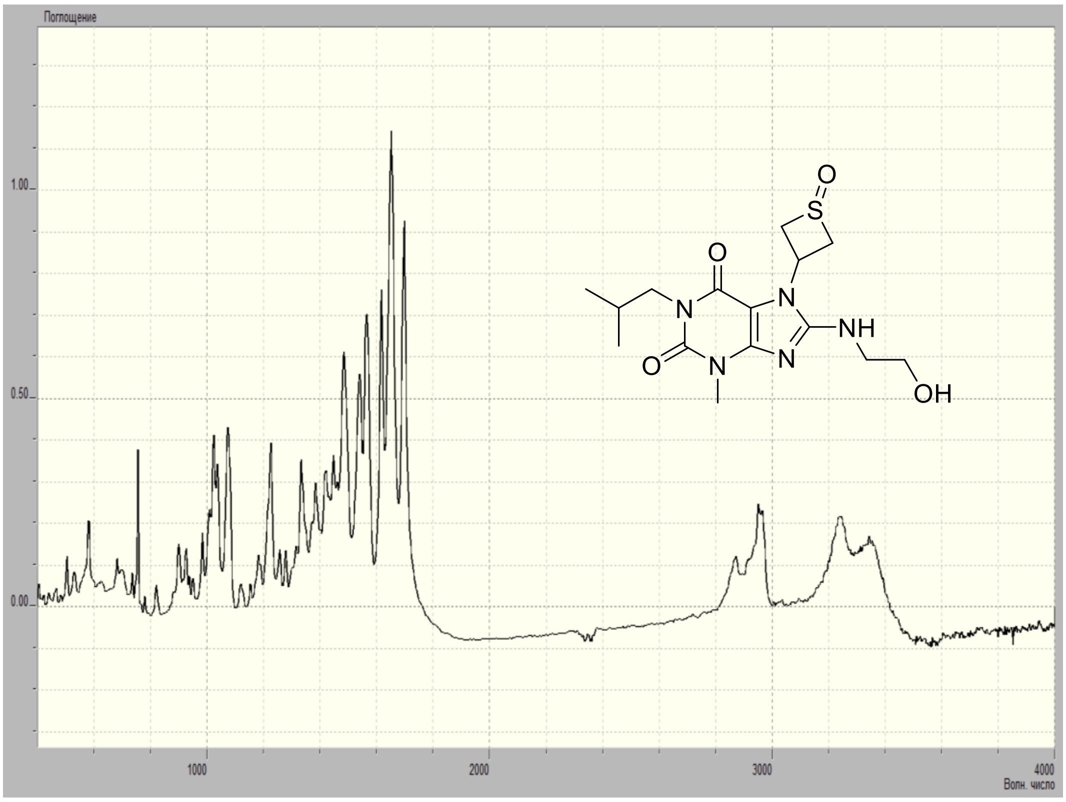

Figure S49: IR spectrum of **6j**

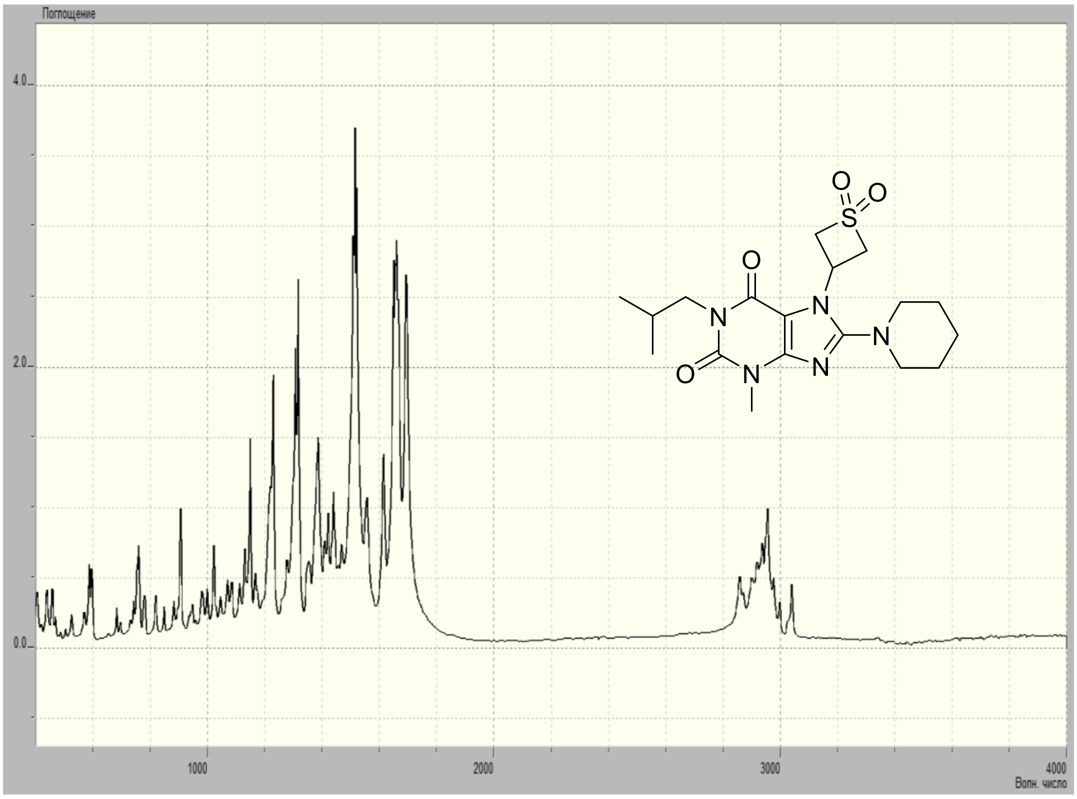

Figure S50: IR spectrum of **6k**

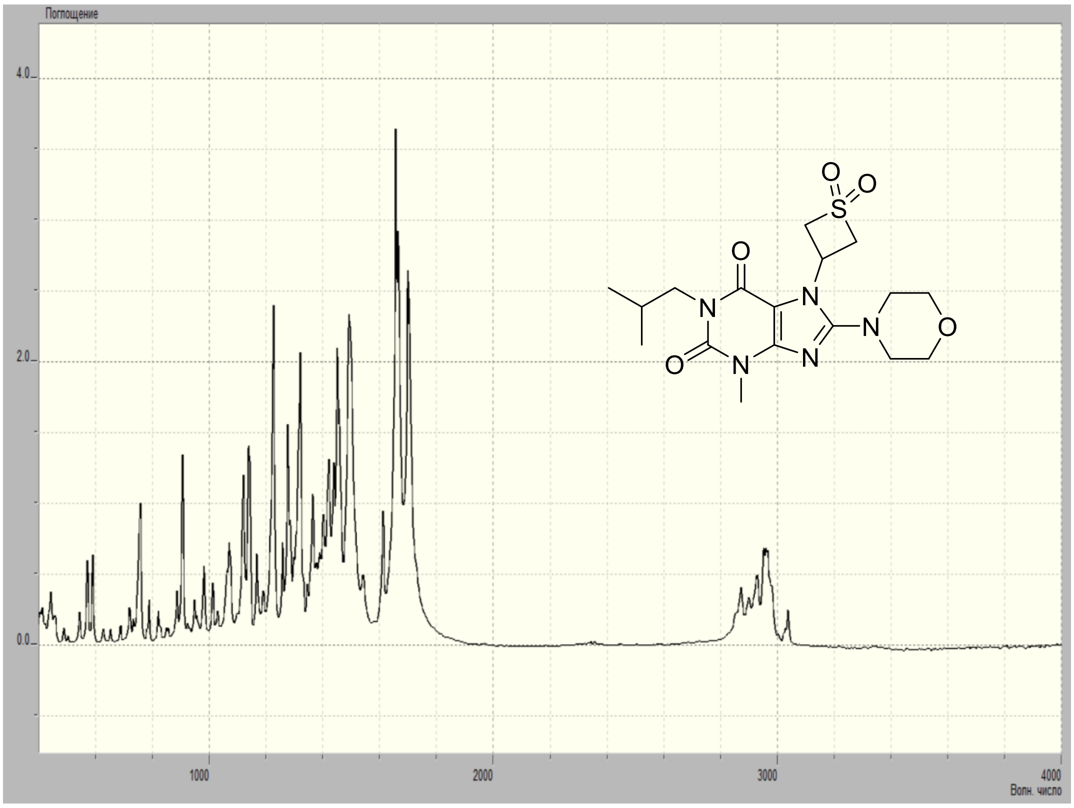

Figure S51: IR spectrum of **6l**

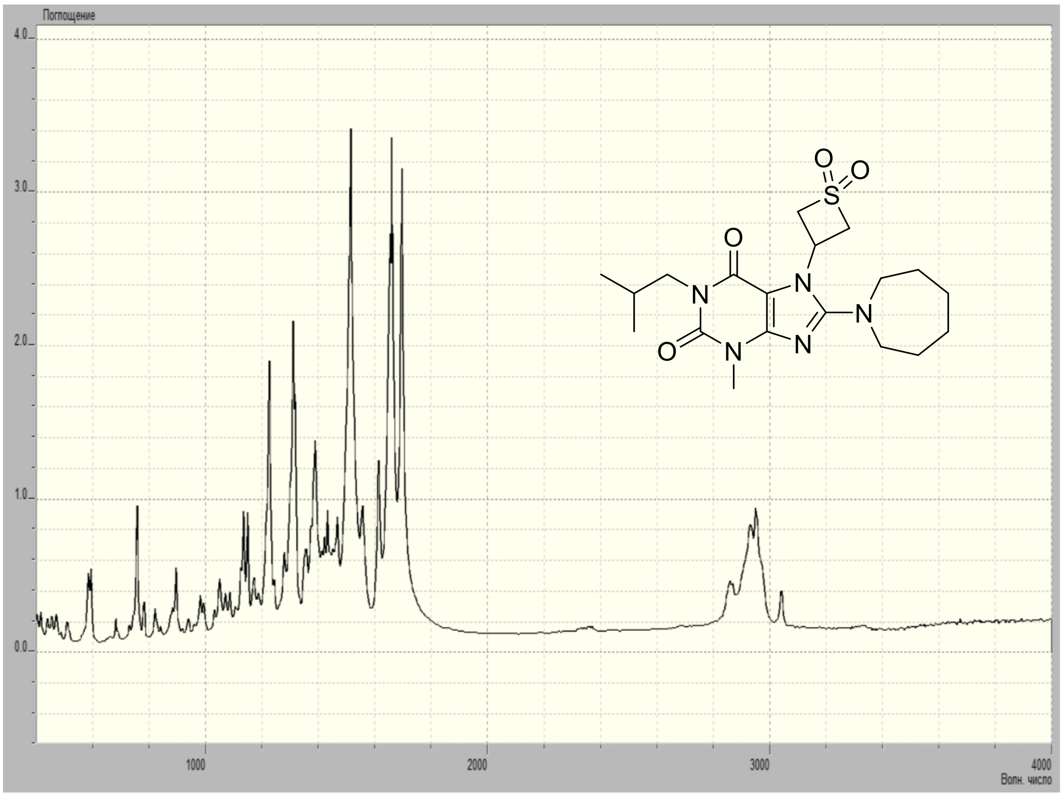

Figure S52: IR spectrum of **6m**

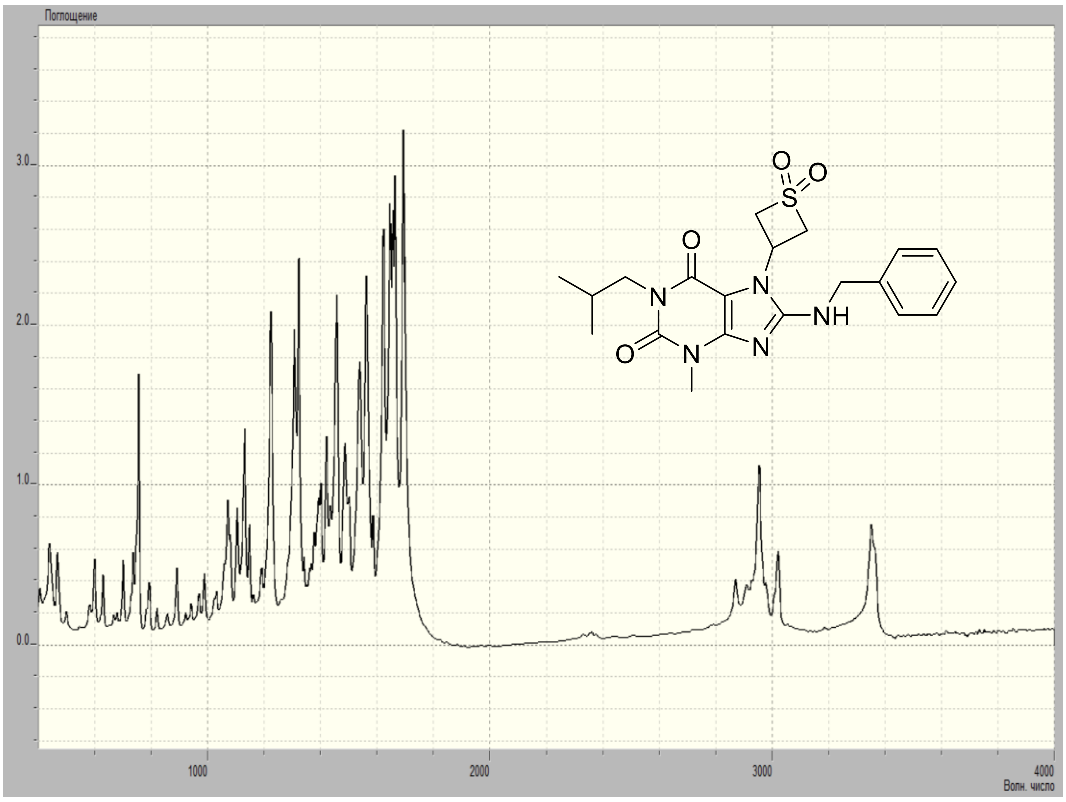

Figure S53: IR spectrum of **6n**

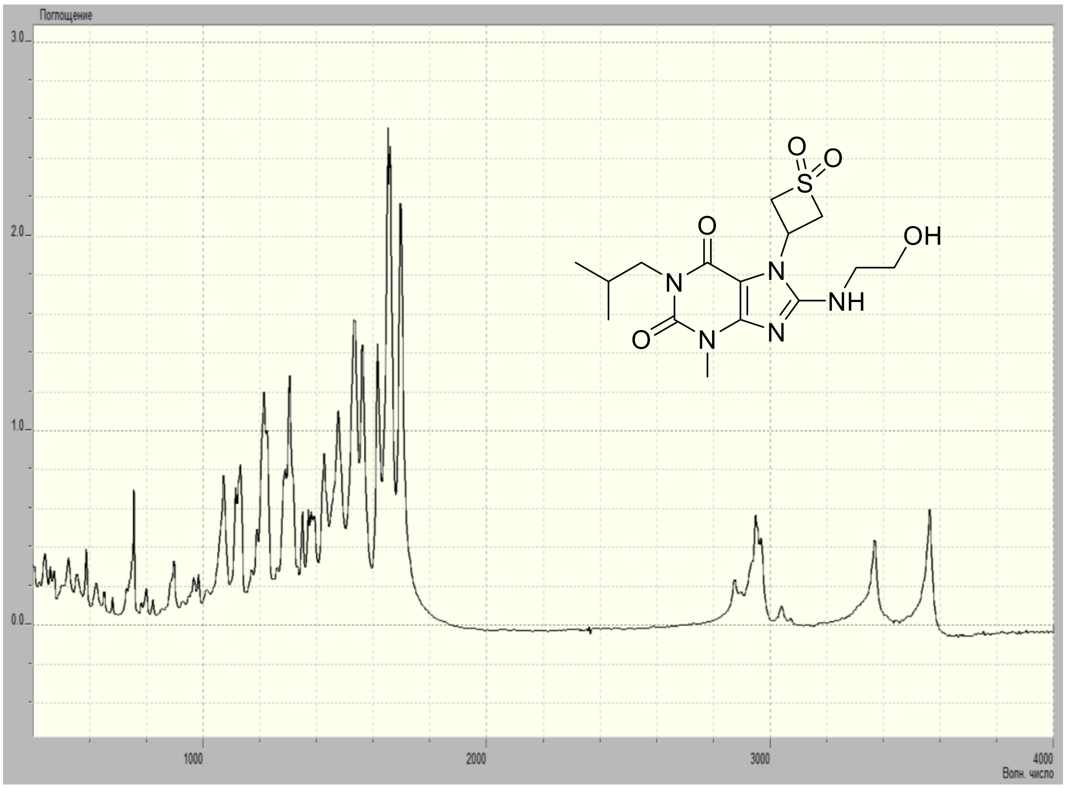

Figure S54: IR spectrum of **6o**
